# A lncRNA-SWI/SNF complex crosstalk controls transcriptional activation at specific promoter regions

**DOI:** 10.1038/s41467-020-14623-3

**Published:** 2020-02-18

**Authors:** Elena Grossi, Ivan Raimondi, Enrique Goñi, Jovanna González, Francesco P. Marchese, Vicente Chapaprieta, José I. Martín-Subero, Shuling Guo, Maite Huarte

**Affiliations:** 1 Department of Gene Therapy and Regulation of Gene Expression, Center for Applied Medical Research, University of Navarra, Pamplona, 31008 Spain; 2 Institute of Health Research of Navarra (IdiSNA), Pamplona, Spain; 3 Departament de Fonaments Clinics, Facultat de Medicina, Universitat de Barcelona, Barcelona, Spain; 4 Institut d’Investigacions Biomèdiques August Pi i Sunyer (IDIBAPS), Barcelona, Spain; 5 Institució Catalana de Recerca i Estudis Avançats (ICREA), Barcelona, Spain; 6 Centro de Investigación Biomédica en Red de Cáncer (CIBERONC), Madrid, Spain; 7 Department of Antisense Drug Discovery and Clinical Development, Ionis Pharmaceuticals, Carlsbad, CA USA

**Keywords:** Cancer, Epigenetics, Gene regulation, Chromatin, Non-coding RNAs

## Abstract

LncRNAs have been shown to be direct players in chromatin regulation, but little is known about their role at active genomic loci. We investigate the role of lncRNAs in gene activation by profiling the RNA interactome of SMARCB1-containing SWI/SNF complexes in proliferating and senescent conditions. The isolation of SMARCB1-associated transcripts, together with chromatin profiling, shows prevalent association to active regions where SMARCB1 differentially binds locally transcribed RNAs. We identify *SWINGN*, a lncRNA interacting with SMARCB1 exclusively in proliferating conditions, exerting a pro-oncogenic role in some tumor types. *SWINGN* is transcribed from an enhancer and modulates the activation of *GAS6* oncogene as part of a topologically organized region, as well as a larger network of pro-oncogenic genes by favoring SMARCB1 binding. Our results indicate that *SWINGN* influences the ability of the SWI/SNF complexes to drive epigenetic activation of specific promoters, suggesting a SWI/SNF-RNA cooperation to achieve optimal transcriptional activation.

## Abstract (web summary)

SWI/SNF complexes regulate chromatin architecture and gene expression. Here the authors report the RNA interactome of SMARCB1-containing SWI/SNF complexes, showing the function of SMARCB1-interacting long noncoding RNA *SWINGN* in transcriptional activation of *GAS6* and a set of SWI/SNF target genes.

## Introduction

A very significant portion of the genome can be transcribed upon different stimuli, giving rise to thousands of noncoding RNAs (ncRNAs). Among them, long noncoding RNAs (lncRNAs) refer to non-protein coding transcripts longer than 200 nucleotides, a broad definition that includes different types of RNAs. Despite not being translated to proteins lncRNAs are known to play an active part in multiple cellular processes through diverse mechanisms^1^. Numerous studies have addressed the function of lncRNAs in gene silencing^2–4^, however less is known about their role at active genomic regions, where many lncRNAs are transcribed. This is the case of enhancer RNAs (eRNAs), a particular class of lncRNAs whose production at enhancers has been directly related to enhancer activity^5,6^, although their specific role in this chromatin context is still debated. In addition, it has been proposed that some enhancer-containing loci harbor lncRNAs with a similar function to eRNAs^7^, stressing the principle that these ncRNA categories are not mutually exclusive. However, their function and relationship with locally bound chromatin factors remain poorly investigated.

SWI/SNF complexes are multimeric ATP-dependent chromatin remodelers that are critical in maintaining chromatin architecture and gene expression^8–10^. They are targeted to regulatory regions, in particular promoters, enhancers and super-enhancers, playing a widespread role in enhancer activation^11,12^. Out of the variety of cellular processes in which SWI/SNF complexes participate, oncogene-induced senescence (OIS) raised particular interest due to the major chromatin reorganization accompanying this process^13–15^. The concomitant gene expression changes aimed at activating this tumor suppressor program are often driven by the cooperative action of different epigenetic complexes, including SWI/SNF^8–10^. Consistently, genes encoding for components of SWI/SNF are mutated in more than 20% of human cancers, being among the most prominent tumor suppressors in humans^16^. The clearest demonstration of SWI/SNF tumor suppressor role are the malignant rhabdoid tumors (MRTs), specifically driven by the biallelic inactivation of the gene encoding for SMARCB1 SWI/SNF core subunit^17^. These tumors present the lowest mutational burden of any human tumor sequenced to date, suggesting that tumorigenesis in MRTs is induced by the epigenetic misregulation consequence of *SMARCB1* loss^18–21^.

A number of recent studies have suggested an intriguing connection between SWI/SNF complexes and lncRNAs^22–26^. Although these studies raise the exciting possibility that distinct lncRNAs could modulate different facets of SWI/SNF activity, the extent and functional outcome of the interaction between lncRNAs and SWI/SNF still remains unknown. Here we investigate the relationship between the SWI/SNF and lncRNAs transcribed in *cis*, revealing a functional interdependence between the complexes and the lncRNA *SWINGN* in promoter-enhancer regulation with consequences in cell transformation.

## Results

### SMARCB1 specifically binds to distinct transcripts

In order to increase our understanding of the role of lncRNAs at active chromatin regions, we set out to investigate the interaction between lncRNAs and the SWI/SNF complexes. We hypothesized that relevant interactions should be dynamic through processes that implicate strong chromatin changes. For this reason, we initially used as experimental model a controlled cellular process in which the SWI/SNF complexes exert a major role, i.e., the induction of cellular senescence^8,10^. We mimicked cellular senescence in vitro by infecting human BJ fibroblasts with an oncogenic form of *hRAS*, which, once induced by 4-Hydroxytamoxifen (4OHT) administration, can promote senescence induction. The correct onset of oncogene-induced senescence (OIS) was verified by the expected arrest in cell proliferation and induction of inflammatory factors and tumor suppressor proteins (Supplementary Fig. 1A–C).

**Fig. 1:**
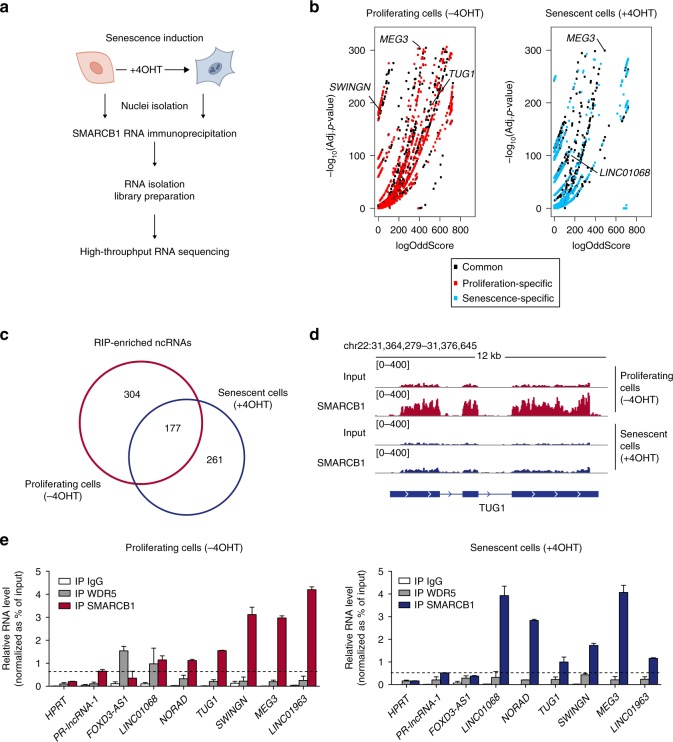
SMARCB1 RIP-seq reveals differential interactions with RNA upon senescence induction. **a** Schematic workflow of SMARCB1 RIP followed by high-throughput sequencing performed on proliferating BJ ER:RAS cells or induced to senescence after 6 days of OIS; *n* = 3 biological replicates. **b** Volcano plot representing enriched SMARCB1-interacting RNA molecules in proliferating (−4OHT; left panel) or senescent (+4OHT; right panel) conditions, as calculated by ripseeker package (Materials and methods). On *x*-axes, logOddScore values correspond to fold induction value of IP/Input while adjusted *p*-value (Adj. *p*-value) based on posterior probability is plotted on *y*-axes. Transcripts enriched in both conditions are represented as black dots while proliferation or senescence-specific RNAs are labeled in red or blue, respectively. **c** Venn diagram showing the overlap between ncRNAs (which include lincRNA, antisense, miscRNA, scaRNA, and miRNA) enriched in SMARCB1 IP in proliferating (*n* = 481, red-colored) and senescent conditions (*n* = 438, blue-colored). Significantly enriched ncRNAs were selected based on the following filters: Adj. *p*-value ≤ 1e^−10^, logOddScore > 1. **d** RIP-seq signal of Input and SMARCB1 IP at *TUG1* genomic locus in proliferating (red color, −4OHT) and senescent (blue color, +4OHT) BJ cells. **e** Validation of a set of lncRNAs by SMARCB1 RIP followed by RT-qPCR. RNA enrichment in SMARCB1 IP was calculated as percentage of input, using WDR5 and IgG IPs as control. *SWINGN* was amplified with primer set#2. *HPRT* and *p53-regulated lncRNA-1* (*PR-lncRNA-1*) are negative controls. *n* = 3 (*n* = 2 for WDR5 RIP) biological replicates shown as mean ± SD. Source data of this Figure are provided as Source data file.

To identify the RNAs associated to the SWI/SNF complexes, we focused on SMARCB1, a core subunit common to all SWI/SNF complexes. We immunoprecipitated SMARCB1 from nuclear extracts of proliferating (−4OHT) and senescent (+4OHT) BJ fibroblasts and retrieved the interacting RNAs by native RNA immunoprecipitation (RIP) assay (Fig. 1a and Supplementary Fig. 1D). SMARCB1 retrieving was effective in isolating the SWI/SNF complexes since it recovered other core subunits, such as SMARCC1 (Supplementary Fig. 1E) as well as readily detectable amounts of RNA. We then performed high-throughput sequencing of the total (Poly A^+^ and Poly A^−^) RNA molecules interacting with SMARCB1, which identified hundreds of enriched RNAs compared to the correspondent Input (Fig. 1b and Supplementary Data 1–2). Among the SMARCB1-associated noncoding RNAs, 177 were commonly bound by SMARCB1 in both conditions, while 304 were specifically enriched in proliferating cells and 261 in senescent cells (Adj. *p*-value ≤ 1e^−10^; logOddScore > 1) (Fig. 1c, d and Supplementary Fig. 1F). Of note, we also found mRNAs commonly or specifically interacting with SMARCB1 in proliferating or senescent conditions (Supplementary Fig. 1G).

To investigate whether the observed differential binding was due to a change in expression upon senescence induction, we compared the enrichment of SMARCB1-interacting RNAs with their differential expression (Supplementary Fig. 1H). The correlation between the differential binding to SMARCB1 and the change in gene expression upon OIS was low (*R* = 0.22), suggesting that the enrichment in RNA–SMARCB1 interactions could not be explained by the differential availability of the RNA molecules.

We then focused our attention on the ncRNAs bound to SMARCB1. We selected a subset of both annotated and uncharacterized lncRNAs, confirming their specific enrichment by RIP-qPCR in proliferating and/or senescence conditions (Fig. 1d, e and Supplementary Fig. 1F). In contrast, the immunoprecipitation of WDR5, a regulatory subunit of the MLL activator complex known to bind RNA, did not retrieve the lncRNAs isolated by SMARCB1 RIP with the same efficiency, although it pulled down its previously reported interactor *FOXD3-AS1*^27^ in proliferating cells (Fig. 1e). To further examine the nature of the observed interactions, we performed SMARCB1 immunoprecipitation coupled with UV irradiation (CLIP). Under these astringent conditions the association between SMARCB1 and some of the identified lncRNAs, including *LINC00565*, from now on called *SWINGN* (*SWI/SNF Interacting GAS6 enhancer Noncoding RNA)*, was maintained, indicating a direct binding between them and SWI/SNF (Supplementary Fig. 1I). On the other hand, CLIP of SUZ12, a core subunit of the Polycomb Repressor Complex 2 (PRC2), failed to enrich for the same SMARCB1 lncRNAs, while it efficiently pulled down lncRNAs previously reported as PRC2 binders^3,4,28,29^ (Supplementary Fig. 1J).

These results indicate that the SWI/SNF complexes specifically bind distinct lncRNAs in proliferative and senescent cells, which lead us to hypothesize a potential functional relevance of the interaction between this remodeling complex and a particular set of lncRNAs.

### SMARCB1 preferentially localizes to active chromatin

**Fig. 2:**
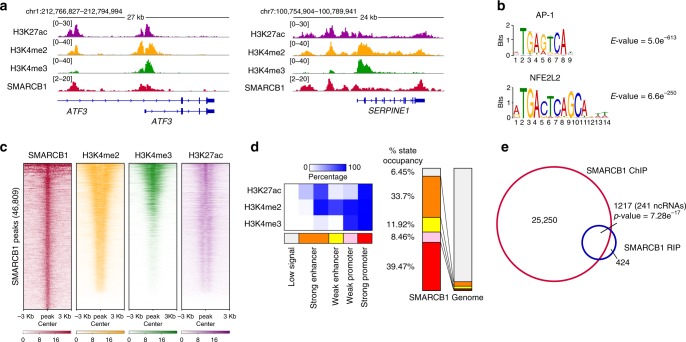
Profiling of SMARCB1 genomic binding map highlights preferential binding at active, transcribed chromatin regions. **a** Genomic snapshots of SMARCB1, H3K27ac, H3K4me2 and H3K4me3 ChIP-seq data at *ATF3* and *SERPINE1* loci in BJ proliferating cells. **b** Top enriched consensus motifs, as defined by MEME-ChIP motif analysis for the peaks in SMARCB1 ChIP-seq experiment in BJ cells. MEME-ChIP *E*-value estimates the expected number of motifs with similar features that one would find in a similarly sized set of random sequences. **c** Heat maps of SMARCB1, H3K4me2, H3K4me3, and H3K27ac occupancy at SMARCB1 peaks identified by SMARCB1 ChIP-seq in BJ cells. Heat maps are ranked by SMARCB1 occupancy. **d** Left, emissions of the chromatin state model generated by ChromHMM using histone marks ChIP-seq data in BJ cells, representing the percentage of regions assigned to a particular chromatin state (columns) containing a specific histone mark (rows). Right, distribution of the different states in the SMARCB1 binding map compared to the distribution in BJ total genome. **e** Venn diagram representing the overlap between genes enriched in SMARCB1 RIP in proliferating conditions (*n* = 1641, including 481 ncRNAs, 980 mRNAs, and 180 pseudogenes) and genes presenting a SMARCB1 ChIP peak (26467). Significance (upper cumulative *p*-value) has been calculated by hypergeometric test.

To investigate whether the interaction between lncRNAs and SMARCB1 takes place in *cis*, that is, at DNA loci bound by the chromatin complexes, we mapped SMARCB1 binding to the chromatin by ChIP-seq, which had only been previously established in some cancer cell lines^22,30^. In parallel, we demarcated the regulatory regions of the genome by generating ChIP-seq data for the histone marks H3K4me2 and H3K4me3, since their ratio allows distinguishing between enhancer and promoter regions^31^, as well as H3K27ac (enriched at active chromatin). All these studies were performed in proliferating fibroblasts (Fig. 2a), the experimental condition in which we observed a higher number of RNA species interacting with SMARCB1.

SMARCB1 ChIP-seq identified about 47 × 10^3^ peaks (FDR < 0.01), a number similar to that already found for other components of the SWI/SNF complexes^32^. Motif analysis of SMARCB1 sites revealed a selective enrichment for known motifs such as the one recognized by AP-1 (*E*-value 5.0 e^−613^, Fig. 2b and Supplementary Fig. 2A), a sequence-specific transcription factor already described as involved in SWI/SNF complex recruitment^33^ and in enhancer regulation upon senescence induction^34^. These data suggest that SMARCB1 ChIP-seq is mapping SWI/SNF binding loci.

We found that SMARCB1 is enriched at genomic loci marked as promoters (high H3K4me3/H3K4me2 ratio) or enhancers (low H3K4me3/H3K4me2 ratio) (Fig. 2c), presenting a large overlap with H3K27ac signal. Consistently, the chromatin state model generated with ChromHMM^35^ showed that SMARCB1 binding peaks present higher percentage of occupancy in regions corresponding to active promoters and strong enhancers (Fig. 2d and Supplementary Fig. 2B).

Since SMARCB1 is present at actively transcribed chromatin regions, we wondered whether the gene loci bound by the SWI/SNF complexes harbored SMARCB1-interacting RNAs identified by RIP approach (Fig. 1c). Indeed, by cross-comparing ChIP and RIP-seq enriched genes, we observed 1217 transcripts interacting with SMARCB1 in *cis*, including 241 ncRNAs (Fig. 2e and Supplementary Data 3). These represent 74% of SMARCB1-interacting transcripts, a number higher than expected by chance (Supplementary Fig. 2C). Our findings demonstrate that SMARCB1 is able to bind active promoter and enhancer regions and to interact with coding and noncoding RNAs transcribed at some of these loci.

### *SWINGN* enhancer locus controls *GAS6* expression

**Fig. 3:**
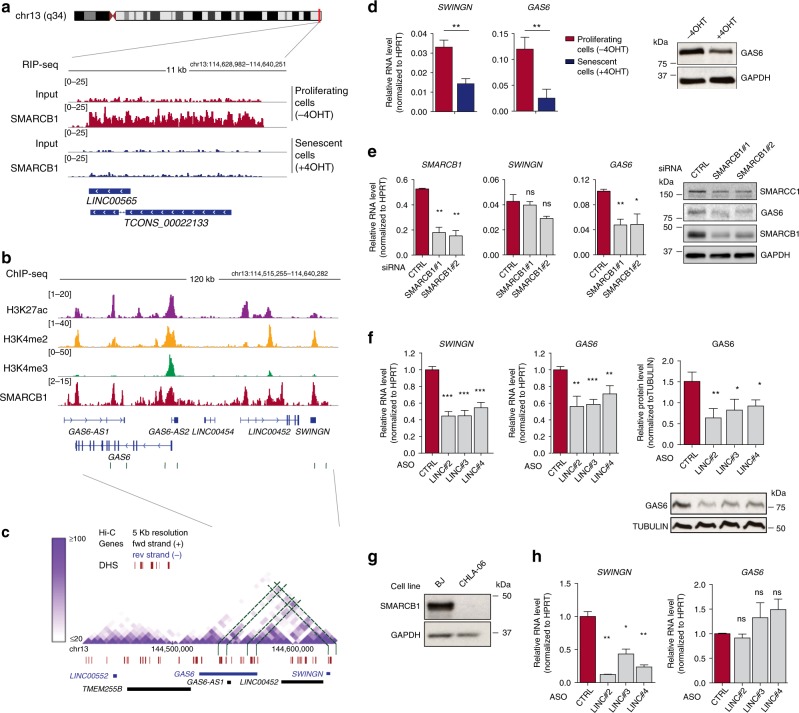
*SWINGN* lncRNA controls the expression of its neighbor gene through SMARCB1 as part of a chromatin domain. **a** Genomic snapshot of SMARCB1 and Input RIP-seq signal at *SWINGN* locus in proliferating (−4OHT; red-colored) and senescent (+4OHT; blue-colored) BJ cells. **b** SMARCB1 and histone marks ChIP-seq signal at *SWINGN*/*GAS6* locus in proliferating BJ fibroblasts. Gray boxes indicate SMARCB1 peaks. **c** High-resolution Hi-C analysis of proliferating IMR90 fibroblasts at *SWINGN*/*GAS6* locus^37^. Green lines highlight regions of stronger chromatin interaction. Red segments indicate DNAse Hypersensitivity Sites (DHS). Gray box highlights the region depicted in **b**. Data are downloaded from http://promoter.bx.psu.edu. **d** RT-qPCR (left) and western blot analyses (right) of proliferating (−4OHT) and senescent (+4OHT) BJ fibroblasts. Graph shows mean ± SD of three independent experiments (left). Western blot picture refers to a representative experiment (*n* = 2). **e** RT-qPCR (left) and western blot analyses (right) of BJ proliferating cells depleted or not of SMARCB1 by siRNA double transfection. Graph shows mean ± SD of independent experiments. Western blot picture refers to a representative experiment (*n* = 2). **f** (Left) RT-qPCR analysis of BJ fibroblasts depleted of *SWINGN* using three different ASOs. Graphs show mean ± SEM of normalized values from *n* = 3. RNA levels were normalized to ASO_CTRL values. (Right) Western blot analysis of BJ cells depleted of *SWINGN*. Mean ± SD of three independent experiments. Western blot image of a representative experiment. **g** Western blot analysis of BJ fibroblasts and CHLA-06 AT/RT cells. GAPDH was used as loading control. **h** RT-qPCR analysis of CHLA-06 cells depleted of *SWINGN* using three different ASOs. Graphs show mean ± SD of two independent experiments. RNA levels were normalized to ASO_CTRL values. Source data underlying panels **d**–**h** are provided as a Source Data file. Significance was determined by two-tailed Student’s *t*-test and summarized as follows: not significant (ns); * < 0.05; ** < 0.01; *** < 0.001.

The observation that the majority of SMARCB1-associated lncRNAs are transcribed from regions of active chromatin bound by SMARCB1 itself, prompted us to investigate with more detail the regulation of one of these loci. In particular, we focused our attention on the uncharacterized lncRNA, *SWINGN*, being (i) one of the lncRNAs most significantly interacting with SMARCB1 (Fig. 1e and Supplementary Data 1), (ii) associated to the protein preferentially in proliferating but not in senescence conditions (Fig. 3a), (iii) able to specifically interact with purified SMARCB1 in vitro (Supplementary Fig. 3A, B), and (iv) transcribed from a genomic locus bound by SMARCB1 (Fig. 3b).

*SWINGN* is an intergenic lncRNA located in the long arm of chromosome 13 presenting two annotated isoforms, a shorter one of 2.3 Kb known as *LINC00565* and a longer one of 7.5 Kb annotated as *TCONS_00022133*, according to GENCODE v31 and the human lincRNA body map catalogs, respectively (Fig. 3a). *SWINGN* locus presents typical enhancer features in BJ fibroblasts (Fig. 3b) and in different cell lines of ENCODE catalog^36^ (Supplementary Fig. 3C), along with a binding peak for SMARCB1, suggesting a potential role as an enhancer RNA (eRNA). In line with this observation, the analysis of raw strand-specific sequencing data revealed a certain level of bidirectional transcription, although the transcripts generated from the minus strand showed greater expression and were the forms preferentially bound by SMARCB1 (Supplementary Fig. 3D, E). In addition, droplet digital PCR estimated *SWINGN* to be present with ~8 copies per cell (Supplementary Fig. 3F). Selective reverse-transcription of total or poly-adenylated RNA showed that *SWINGN* lacks a poly-A tail (Supplementary Fig. 3G), though it showed longer half-life than short-lived polyadenylated transcripts such as *cMYC* (*t*/2 = 4.6 h, Supplementary Fig. 3H). Moreover, RNA fractionation confirmed *SWINGN* nuclear enrichment (Supplementary Fig. 3I). Together our data indicate that *SWINGN* is a lncRNA transcribed from an enhancer locus, presenting some of the characteristics previously described for eRNAs.

Since transcriptional enhancers typically regulate genes that are proximally located, we investigated the regulatory interactions involving *SWINGN* locus. The analysis of public high-resolution Hi-C data of human proliferating fibroblasts^37^ showed that *SWINGN* is located in a topologically-associated domain also containing the protein-coding gene Growth Arrest Specific 6 (*GAS6*) (Fig. 3c). Both *SWINGN* and *GAS6* showed a strong reduction in their expression levels upon senescence (Fig. 3d and Supplementary Fig. 4A), mainly due to transcriptional inhibition, as shown by Global Run-On sequencing (GRO-seq) data in proliferating and senescent fibroblasts^34^ (Supplementary Fig. 4B). This transcriptional reduction is accompanied by a concomitant decrease of H3K27ac mark at *GAS6* promoter (Supplementary Fig. 4C), confirming that transcriptional mechanisms are primarily involved in controlling *GAS6* expression. Interestingly, Hi-C data showed the strongest contacts between *SWINGN* gene and two positions corresponding to the transcription start site (TSS) and the gene body of *GAS6* (Fig. 3c). The contact between *SWINGN* and *GAS6* TSS was confirmed with more resolution by 3C technique (Supplementary Fig. 4D, E), suggesting that *SWINGN* locus is an enhancer of *GAS6*. In addition, SMARCB1 is bound to *SWINGN* as well as to *GAS6* promoter and enhancer regions corresponding exactly to the two DNA loci where the strongest chromatin interactions occur (Fig. 3b, c). We therefore hypothesized that the regulation of the protein-coding gene *GAS6* is orchestrated by the SWI/SNF complexes. To experimentally test this notion, we depleted SMARCB1 or SMARCC1 in BJ fibroblasts and found *GAS6* RNA and protein levels strongly affected, indicating that SWI/SNF is required for *GAS6* expression (Fig. 3e and Supplementary Fig. 4F, G).

Taken together, these data show that the SWI/SNF complexes control the transcriptional activation of the proto-oncogene *GAS6* as part of a topologically organized region that is in physical interaction with the enhancer-like *SWINGN* locus.

### *SWINGN* controls *GAS6* expression in a SWI/SNF dependent manner

It has been proposed that cis-acting noncoding transcripts may be required for optimal activity of enhancers^5^, so we wondered whether *SWINGN* transcript would also play a role in this regulation. To test this idea, we depleted the lncRNA in BJ and IMR90 proliferating fibroblasts using three independent ASOs (Fig. 3f and Supplementary Fig. 5A, B). *SWINGN* reduction resulted in a consistent decrease in *GAS6* expression at both RNA and protein levels (Fig. 3f), indicating that *GAS6* is regulated not only by SMARCB1, but also by the RNA product of *SWINGN* locus. This regulation was specific for *GAS6* within this chromatin domain since did not affect other neighbor genes although it did alter the expression of *GAS6-AS2*, a lncRNA sharing *GAS6* promoter (Supplementary Fig. 5A).

To further confirm the functional co-dependency between SMARCB1 and *SWINGN* transcript, we used as control a highly aggressive type of pediatric cancer driven by the biallelic deletion of *SMARCB1* gene^17^. In these tumors, SMARCB1 loss impairs the chromatin affinity of the deficient SWI/SNF complexes, preventing the activation of specific enhancer regions implicated in differentiation^38^. In particular, we focused on atypical teratoid/rhabdoid tumors (AT/RT), an incurable cancer of the central nervous system with loss of *SMARCB1* in ~100% of the cases^39^. We selected the CHLA-06 cell type where SMARCB1 expression is absent (Fig. 3g) and the levels of both *GAS6* and *SWINGN* are higher compared to other AT/RT cells (Supplementary Fig. 5C). Notably, *GAS6* mRNA expression remained unchanged upon *SWINGN* downregulation in these cells. On the other hand, while the enforced expression of SMARCB1 resulted in an increase of *GAS6* levels, this induction was not significantly affected by *SWINGN* depletion (Supplementary Fig. 5D, E). Since it is known that SMARCB1 reintroduction is not sufficient to globally reprogram chromatin contacts in rhabdoid tumor cells^32^, these results suggest that *SWINGN* and SWI/SNF cooperate to regulate the expression of *GAS6* when cell-specific chromatin conformations allow establishing the correct regulatory interactions (Fig. 3h).

### *SWINGN*-mediated control of *GAS6* has implications in cancer

**Fig. 4:**
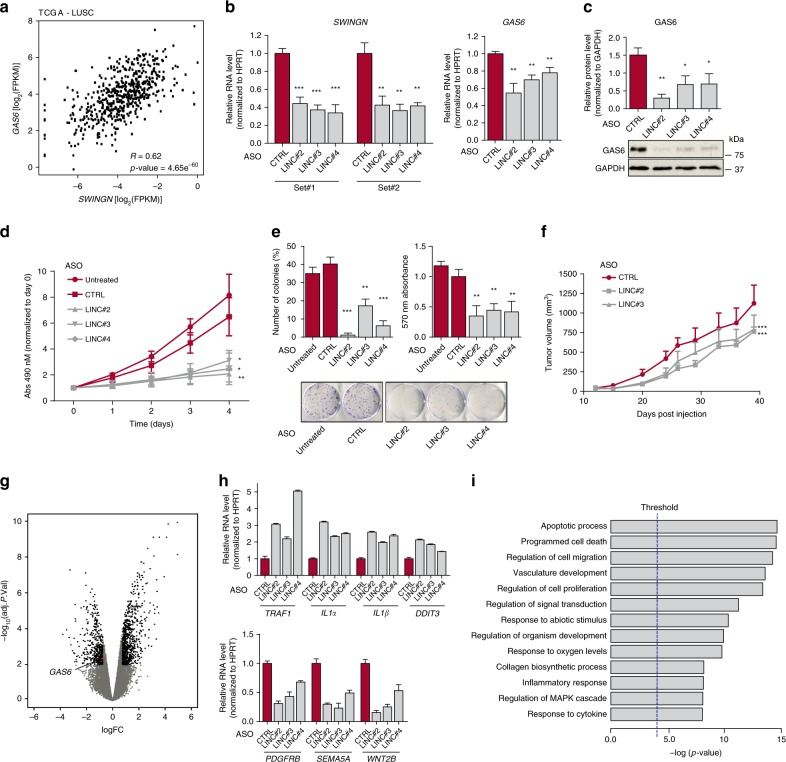
*SWINGN*-mediated GAS6 regulation is relevant in cancer progression. **a** Correlation plot showing *SWINGN* and *GAS6* expression (as FPKM+ pseudocount values) in LUSC samples (*n* = 552) from TCGA database (gdc-portal.nci.nih.gov). Correlation *p*-value is calculated using a t-distribution. **b**, **c** RT-qPCR (**b**) and Western blot (**c**) analysis of H226 lung squamous carcinoma cells depleted of *SWINGN*. RNA levels were normalized to ASO_CTRL and detected with two different primer sets. Graphs show mean ± SEM of normalized values (*n* = 3); image is from a representative experiment. **d** MTS proliferation assay of control and *SWINGN*-depleted H226 cells. Absorbance values were normalized to day 0. Graph shows mean ± SEM (*n* = 3). *T*-test *p*-value is calculated with data points at day4: * < 0.05; ** < 0.01. **e** Clonogenicity assay of control and *SWINGN*-depleted H226 cells. Number of colonies (left) and cell density by absorbance (right) are shown. Graph shows mean ± SD of two independent experiments, picture refers to a representative experiment. **f** Analysis of tumors generated by subcutaneous injection of control or *SWINGN*-depleted H226 cells in BALB/cA-Rag2−/−γc−/− mice. Graph shows tumor size mean ± SEM (*n* = 7); statistical significance was calculated using one-way ANOVA and Bonferroni’s Multiple Comparison Test compared to ASO_CTRL. **g** Volcano plot of RNA-seq analysis of H226 cells depleted of *SWINGN* using ASO_LINC#3 or treated with control ASO. Black dots indicate genes with a significant change comparing the two conditions (Adj. *p*-value < 0.01; logFC ± 0.5). Adj. *p*-value is calculated by quasi-likelihood (QL) *F*-test with Benjamini-Hochberg correction. **h** RT-qPCR validation of a selection of the most affected genes obtained in the RNA-seq analysis in H226 cells. Upper and lower panels show upregulated and downregulated gene candidates, respectively. RNA levels were normalized to ASO_CTRL values. **i** Gene Ontology (GO) analysis illustrating the biological processes enriched for the 1641 differentially expressed genes identified in RNA-seq data of control and *SWINGN*-depleted H226 cells. Threshold: *p*-value < 10^−4^. *p*-value derived by random sampling of the whole genome combined with Fisher’s inverse chi-square method. Source data for panels **b**–**h** are provided as a Source Data file. Student’s *t*-test *p*-values in **b**–**e** summarized as follows: * < 0.05; ** < 0.01; *** < 0.001.

The regulatory interaction between *SWINGN* and *GAS6* prompted us to investigate the biological consequences of altering this association. In fact, GAS6 is a secreted protein that binds to receptor tyrosine kinases of the TAM family to activate downstream pathways and promote cell growth and survival^38^. GAS6 function is consistent with the observed decrease of its expression upon OIS (Fig. 3d) and is also in line with a recent report showing that GAS6 overexpression delays senescence onset^40^. Consistently, the knockdown of *SWINGN* in BJ fibroblasts impairs their proliferation and increases apoptosis levels (Supplementary Fig. 6A, B). To get insight into *SWINGN* impact on *GAS6* regulation in cancer we analyzed the correlation between *SWINGN* and *GAS6* expression across samples of different tumor types of The Cancer Genome Atlas (TCGA). Their expression presented a positive correlation in several of the tumor types analyzed, such as lung squamous carcinomas (LUSC) or breast invasive carcinoma (BRCA), while there was no correlation in colon adenocarcinoma (COAD) (Fig. 4a and Supplementary Fig. 6C).

To elucidate whether the regulation of *GAS6* by *SWINGN* is relevant in tumorigenesis, we selected H226 LUSC and HCT116 COAD cell lines, having respectively high and low expression of both *SWINGN* and *GAS6* (Supplementary Fig 6D). Similarly to that observed in normal fibroblasts, in H226 lung cell line SMARCB1 immunoprecipitation retrieved *SWINGN* (Supplementary Fig. 6E), and, *SWINGN* depletion decreased *GAS6* expression (Fig. 4b, c). On the other hand, such dependency was not observed in HCT116 colon cells, which didn’t show a significant decrease of *GAS6* mRNA even though ASO treatment was effective in reducing *SWINGN* levels (Supplementary Fig. 6F), confirming that the regulation of *GAS6* by *SWINGN* is cell type dependent. As consequence of *SWINGN* knockdown, H226 cell proliferation and colony formation capacity were affected concomitantly with an increase in the percentage of apoptotic cells (Fig. 4d, e and Supplementary Fig. 6G). Moreover, H226 cells depleted of *SWINGN* presented a reduced tumor growth rate when injected into immunodeficient mice (Fig. 4f and Supplementary Fig. 6H). On the other hand, *SWINGN* depletion in HCT116 cells did not affect cell growth (Supplementary Fig. 6I–J), consistent with the notion that the effect observed in cell proliferation is specific and dependent on the regulation of *GAS6* by *SWINGN*. To better characterize the effect of *SWINGN* we performed gene expression analysis by RNA-seq on H226 cells upon *SWINGN* inhibition, revealing 1644 differentially expressed genes, whose change was validated in H226 and BJ cells (Fig. 4g, h, Supplementary Fig. 6K and Supplementary Data 4). Gene Ontology analysis on the genes affected showed terms related to apoptosis, cell migration and inflammation (Fig. 4i), in line with the cellular phenotype and the change in expression detected in tumors, indicating the role of *SWINGN* as a pro-oncogenic lncRNA.

### *SWINGN* regulates SWI/SNF complexes at additional gene loci

Our data demonstrate that *SWINGN* regulates the oncogene *GAS6*. However, we observed that the inhibition of *GAS6* did not completely phenocopy the loss of *SWINGN* (Supplementary Fig. 7A–C), and *GAS6* overexpression only partially recovered the effect of *SWINGN* knockdown (Supplementary Fig. 7D). Interestingly, *SWINGN* inhibition affects a large set of genes (Fig. 4g). While many of these gene expression changes might be secondary to *GAS6* downregulation, we speculated that others are caused by *SWINGN* through a SWI/SNF-dependent mechanism.

**Fig. 5:**
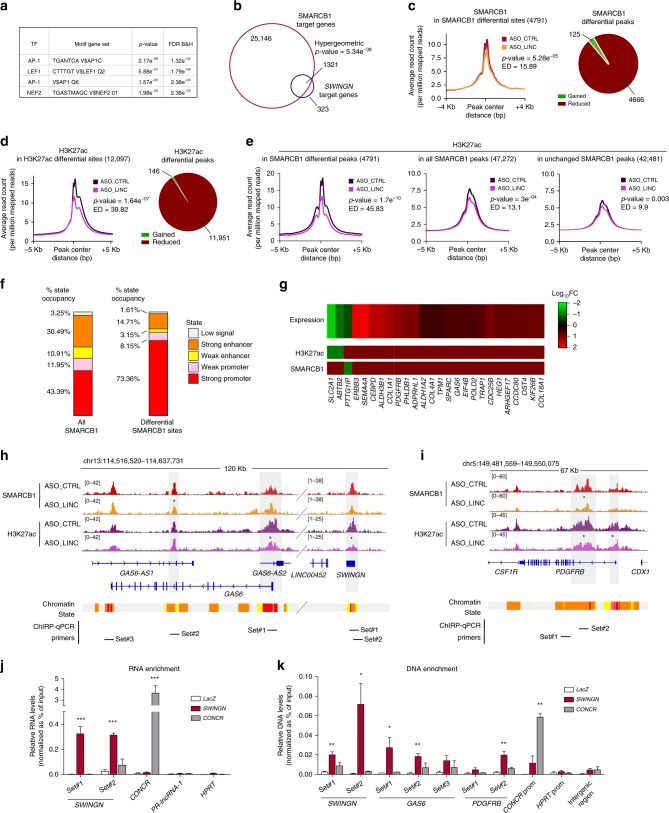
*SWINGN* regulates gene activation at additional loci by controlling SWI/SNF activity. **a** Analysis of transcription factor binding by Gene Set Enrichment Analysis across the Molecular Signatures Database for genes differentially expressed upon *SWINGN* knockdown. FDR is represented as Benjamini-Hochberg corrected. **b** Overlap between genes presenting a SMARCB1 ChIP peak and genes differentially expressed upon *SWINGN* depletion. Significance (upper cumulative *p*-value) has been calculated by hypergeometric test. **c**, **d** Metagene plot showing SMARCB1 (**c**) or H3K27ac (**d**) differential occupancy in control (ASO_CTRL) and *SWINGN* knockdown (ASO_LINC) (left) and pie chart illustrating the composition of the peaks (right). **e** Metagene plot showing H3K27ac differential occupancy in control (ASO_CTRL) and *SWINGN* knockdown (ASO_LINC) in regions with differential binding of SMARCB1 (left), all regions bound by SMARCB1 (center), and regions with unaffected SMARCB1 binding upon *SWINGN* knockdown (right). **c**–**e** Significance has been calculated by *t*-test (represented as *p*-value) while difference between conditions has been measured by Euclidean distance (ED). **f** Distribution of chromatin states at all SMARCB1 peaks (left) and in the regions differentially bound by SMARCB1 upon *SWINGN* knockdown (right). **g** Heat map of selected genes differentially expressed upon *SWINGN* knockdown (RNA-seq) with a SMARCB1 or H3K27ac binding peak changing concordantly upon *SWINGN* depletion. **h**, **i** Genomic snapshot of SMARCB1 and H3K27ac ChIP-seq signals in control (ASO_CTRL) and *SWINGN* knockdown (ASO_LINC) conditions at *GAS6/SWINGN* (**h**) and *PDGFRB* (**i**) loci. Asterisks point out significantly changing peaks. The location of the primers used for ChIRP is indicated below. **j** RNA enrichment in ChIRP experiments with control (LacZ), *CONCR* and *SWINGN* probes determined by RT-qPCR and calculated as percentage of Input for the indicated transcripts. Graph shows mean ± SD of *n* = 5 (*SWINGN*) or *n* = 2 (*CONCR*). **k** DNA enrichment in ChIRP experiments with control (LacZ), *CONCR* and *SWINGN* probes determined by qPCR and calculated as percentage of Input with the indicated primer sets. Graph shows mean ± SEM of n = 4 (*SWING*) or n = 2 (*CONCR*). Source data underlying **j**–**k** panels are provided as a Source Data file. Student’s *t*-test *p*-values are summarized as follows: * < 0.05; ** < 0.01; *** < 0.001.

In support of this hypothesis, the analysis of the promoters of the genes regulated by *SWINGN*^41,42^ showed the most significant enrichment in transcription factor binding motifs linked to the SWI/SNF complexes such as AP-1 and NFE2 (Fig. 5a). In addition, a highly significant number of these genes have a SMARCB1 ChIP-seq peak (*p*-value = 5.43e^−38^, Fig. 5b), confirming that genes regulated by *SWINGN* share their regulation with SWI/SNF.

We excluded that *SWINGN* had an effect on SWI/SNF stability, since we did not observe alterations of the protein levels of different core components upon *SWINGN* knockdown (Supplementary Fig. 7E). We then speculated that *SWINGN* might influence SMARCB1 binding and/or activity at these regulated loci. To address this, we investigated the effect of *SWINGN* depletion on SMARCB1 occupancy in BJ fibroblasts. *SWINGN* inhibition did not cause a strong global effect on SMARCB1 chromatin binding (*p*-value = 0.03) (Supplementary Fig. 8A). However, a subset of SMARCB1 peaks was significantly affected (4791 peaks, *p*-value = 5.28e^−05^), with SMARCB1 signal reduced in 95% of these regions (Fig. 5c and Supplementary Fig. 8C). In parallel, we performed H3K27ac ChIP-seq under the same experimental conditions. The global analysis of H3K27ac occupancy only showed a slight effect upon *SWINGN* knockdown (*p*-value = 0.01) (Supplementary Fig. 8B). In contrast, a stronger effect was observed when focusing on the differential H3K27ac peaks (12,097 peaks, *p*-value = 1.64e^−07^), resulting from a drop of H3K27ac in 99% of these regions (Fig. 5d and Supplementary Fig. 8D).

To assess the relationship between the decrease in SMARCB1 binding and the H3K27ac reduction caused by *SWINGN* depletion, we analyzed H2K27ac signal at the 4791 SMARCB1 differentially bound sites upon *SWINGN* knockdown (Fig. 5c). A significant decrease in H3K27ac occupancy was observed at these sites (*p*-value = 1.7e^−10^) (Fig. 5e). In contrast, the differences were partially reduced when considering all SMARCB1 regions and almost abolished when the SMARCB1 differential peaks were subtracted from the whole set of SMARCB1 regions (Fig. 5e). These data demonstrate that the changes in H3K27ac are mainly associated with the decreased binding of SMARCB1 at specific loci caused by *SWINGN* knockdown.

Next, since it has been shown that the ablation of SMARCB1 can decrease acetylation at enhancers^11^, we analyzed H3K27ac changes upon *SWINGN* knockdown separately at distal enhancer sites and proximal promoter regions. H3K27ac occupancy was mostly altered at regions identified as active promoters (Fig. 5f and Supplementary Fig. 8E, F), suggesting that *SWINGN* depletion affects preferentially SMARCB1 function in driving promoter acetylation.

Given the effect of *SWINGN* at specific promoters, we explored the connection between the observed chromatin changes and the alterations in gene expression induced by *SWINGN* downregulation. Since we had described the role of *SWINGN* in the SWI/SNF-mediated activation of *GAS6*, we focused on genes whose expression and chromatin status were similarly and concordantly affected. Following these premises, we found 85 genes presenting concordantly altered expression, SMARCB1 and H3K27ac binding upon *SWINGN* knockdown (Fig. 5g and Supplementary Data 5). Of note, 276 genes did not show a change in gene expression concordant with SMARCB1 altered binding when *SWINGN* was depleted. We speculate that this may be due to the accumulation of opposed transcriptional and posttranscriptional mechanisms that result in an increase of mRNA steady state levels that do not reflect their epigenetic status.

On the other hand, we predicted that the 85 concordantly regulated genes include the primary *SWINGN*-SMARCB1 targets. As expected, *GAS6* was one of them. SMARCB1 signal was significantly reduced at *GAS6* enhancer region, accompanied by a decreased H3K27ac occupancy at both *GAS6* and *SWINGN* promoter sites (Fig. 5h), and corresponding with the downregulation of *GAS6* mRNA levels. Besides *GAS6*, other genes of the same chromosomal region (13q34) appeared in the gene set co-regulated by *SWINGN* and SMARCB1 (Supplementary Fig. 8G). Intriguingly, the set of affected genes also included genes localized in different chromosomes already known to be dependent on SMARCB1 status, such as *Platelet Derived Growth Factor Receptor Beta* (*PDGFRB*). This gene, recently found altered in AT/RT and elected as a novel therapeutic target for these tumor types^21^, showed similar chromatin changes upon *SWINGN* knockdown, corroborating the hypothesis of a regulation orchestrated by SMARCB1 and *SWINGN* (Fig. 5i).

Since our data show that *SWINGN* and SMARCB1 physically interact and co-regulate several genes, we predicted that the lncRNA should be present at the co-regulated genomic loci. By applying Chromatin Isolation by RNA Purification (ChIRP) technique we found that the probes that specifically pulled down *SWINGN* (Fig. 5j) not only showed enrichment at its own genomic locus as expected, but also at additional positions where SMARCB1 binds in a *SWINGN*-dependent manner, including *GAS6* and *PDGFRB* regions (Fig. 5k). In contrast, no significant enrichment was detected at *HPRT* promoter, bound by SMARCB1 but not regulated by *SWINGN*, or at a non-expressed intergenic locus (Fig. 5k). On the other hand, ChIRP of *CONCR* lncRNA, which is expressed at similar levels (Supplementary Fig. 8H), did not show a similar enrichment pattern (Fig. 5j, k).

These data are in accordance with the direct role of *SWINGN* in promoting SMARCB1 binding at specific loci to promote their activation.

### *SWINGN* activates a pro-oncogenic gene network

**Fig. 6:**
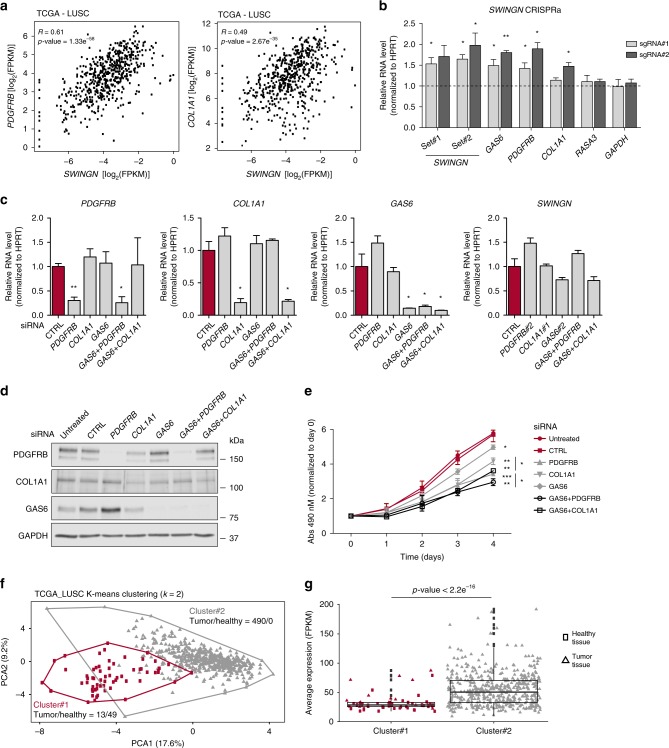
*SWINGN* controls a pro-oncogenic hub predictive of cancer status. **a** Correlation plot showing *SWINGN* and *PDGFRB* or *COL1A1* expression levels (as FPKM+ pseudocount values) in LUSC samples (*n* = 552) from TCGA database. Correlation *p*-values are calculated using a *t*-distribution. **b** RT-qPCR analysis of H226 cells stably expressing dCAS9-VP64 fusion protein and infected with lentiviral vectors carrying two independent sgRNAs targeting *SWINGN* promoter or an empty vector. Two different primer sets were used to detect *SWINGN* transcript. Graph shows mean ± SD of normalized values from two independent experiments. **c** RT-qPCR analysis of H226 cells treated with siRNAs depleting *PDGFRB* (siRNA#2) or *COL1A1* (siRNA#1) alone or in combination with *GAS6* knockdown (siRNA#2). Graphs show mean ± SD of three independent experiments. RNA levels were normalized to CTRL siRNA conditions. **d** Western blot analysis of H226 cells depleted of *PDGFRB* (siRNA#2) or *COL1A1* (siRNA#1) alone or in combination with *GAS6* (siRNA#2). Picture refers to a representative experiment out of two performed. **e** MTS proliferation assay of H226 cells treated with siRNAs depleting *PDGFRB* or *COL1A1* alone or in combination with *GAS6* knockdown at the indicated time points. Absorbance values were normalized to day 0. Graph shows mean ± SEM of three independent experiments. Significance has been calculated comparing each condition to CTRL siRNA sample. Conditions of *PDGFRB* and *COL1A1* knockdown in combination with *GAS6* were also compared to single knockdown samples. **f** K-means clustering of 552 LUSC_TCGA samples according to the expression of *SWINGN* gene signature (27 genes). Clusters are represented as red-labeled cluster#1 (comprising 13 tumor samples and 49 adjacent healthy tissues) and gray-colored cluster #2 (including 490 cancer tissues). **g** Box plot illustrating average expression of *SWINGN* gene signature in each LUSC_TCGA sample grouped according to cluster division. Squared dots represent healthy tissues while triangular points refer to tumor status. Statistics was calculated by Student’s *t*-test. Source data underlying **b**–**e** panels are provided as a Source Data file. Student’s *t*-test *p*-values are summarized as follows: * < 0.05; ** < 0.01; *** < 0.001.

We hypothesized that the subset of genes co-regulated by *SWINGN* and SMARCB1 might contribute to the observed role of the lncRNA in promoting the proliferation of cancer cells. Interestingly, we observed a positive correlation between *SWINGN* and several of its predicted targets across LUSC TCGA data set, including *PDGFRB* as well as *Collagen Type I Alpha 1 Chain* (*COL1A1*), another gene with known oncogenic function (Fig. 6a and Supplementary Fig. 9A)^43–45^. The transcriptional activation of these genes is highly dependent on *SWINGN* expression, as we demonstrated by upregulating its levels by CRISPR activation (CRISPRa) in H226 lung cancer cells. The induction of *SWINGN* resulted in a concomitant upregulation of not only *GAS6*, but also *PDGFRB* and *COL1A1* mRNAs without affecting the expression of control genes (Fig. 6b). In line with the oncogenic role of these genes, we observed a decrease in proliferation of H226 cells when either *PDGFRB* or *COL1A1* were inhibited, additive to the effect achieved with *GAS6* downregulation alone (Fig. 6c–e and Supplementary Fig. 9B–D), supporting the existence of a *SWINGN-*dependent oncogenic hub in this cancer type.

Finally, to explore the potential clinical relevance of the SMARCB1 and *SWINGN-*activated gene network, we selected the 27 top genes out of the 85 co-regulated by *SWINGN* and SMARCB1, and computed their expression across the LUSC cohort of TCGA, which includes 503 tumors and 49 samples of adjacent healthy tissue. Principal component analysis (PCA) and k-means algorithm based on the expression of this signature separated the samples into two clusters (Fig. 6f). Notably, cluster#1 contained all of the healthy tissues plus only 13 tumors, while cluster#2 included 490 tumors but not healthy tissue samples (Fig. 6f), suggesting a relationship between the transformed status and the expression of the SMARCB1- *SWINGN- *signature. In agreement with the *SWINGN* pro-proliferative role associated with gene activation, the average expression of the signature was elevated in the tumor samples conforming cluster#2 as compared to cluster#1 samples, mostly composed of normal tissue samples (Fig. 6g).

Thus, as a whole, our data indicate that *SWINGN* is necessary to drive SMARCB1 and the SWI/SNF complexes on a particular set of genes to promote their transcriptional activation, and the coordinated regulation of this gene set by *SWINGN* uncovers a gene expression network that contributes to the transformed phenotype of cancer cells.

## Discussion

The nature of the molecular signals driving the chromatin changes at certain genes while excluding others is still debated, in particular in cellular contexts where potent chromatin changes reshape gene expression, as in the case of oncogene-induced senescence. In this scenario, lncRNAs have raised great interest due to their specificity in expression and diversification of functions, including their capacity to interact with chromatin remodeling complexes to fine-tune their activities in a wide range of cellular contexts.

Our study takes an unbiased approach to isolate RNA molecules interacting with SMARCB1, a core component of the multimeric SWI/SNF complexes. While different studies reported the RNA binding capacity of SMARCA4, the main ATPase of SWI/SNF complexes^26^, it is possible that other proteins of SWI/SNF complex, including SMARCB1, can directly bind RNA through non-canonical RNA-binding domains. Interestingly, analyses of SMARCB1 protein sequence and structure point to this potential; predictor algorithms as FFPred3^46^ assign RNA binding function terms to SMARCB1, in line with the presence of a low complexity region (LCR), a type of domain often related with RNA binding capacity of proteins. We found that the association between SMARCB1 and some of the identified RNA interactors is maintained under UV-crosslinking conditions in vivo, while in vitro, purified SMARCB1 binds to *SWINGN* with more affinity than other unrelated RNAs, supporting the hypothesis that SMARCB1 has the capacity to establish direct interactions with some RNA partners. The biological specificity of the in vivo interaction is likely the combination of an intrinsic biochemical affinity as well as the physical proximity of the RNA and SWI/SNF in the nucleus. Importantly, the identification of lncRNAs differentially bound to SWI/SNF in the dynamic context of senescence induction, suggests the functional relevance for at least some of these interactions.

The RNA-binding capacity of SWI/SNF complexes suggests that RNA-SWI/SNF interactions probably are a prevalent phenomenon, RNAs transcribed from *cis*-regulatory elements having the potential of establishing a mechanism of SWI/SNF-dependent gene regulation. Emerging studies indicate that the interactions between RNA and SWI/SNF can have opposite roles^22,26,47^. While *Xist* antagonizes the ATPase activity of the SWI/SNF core subunit SMARCA4^26^, *7SK* small nuclear RNA directs the SWI/SNF complexes to enhancer elements^47^. We speculate that distinct RNA structures or SWI/SNF associated factors as well as specific chromatin and transcriptional contexts might be key to the different outcomes. Although it is difficult to find a unifying mechanism, our study provides additional evidence of the functional crosstalk between RNA and SWI/SNF complexes.

*SWINGN*, which associates to the chromatin complexes in proliferating but not in senescent cells, represents a relevant example of a SMARCB1-interacting lncRNA. Our detailed study of *SWINGN* locus in human fibroblasts reveals the existence of a regulatory hub formed by the enhancer element harboring *SWINGN*, the SWI/SNF complexes and the lncRNA itself in a genomic region topologically-associated to *GAS6* gene. The presence of SMARCB1, ensuring the formation of intact SWI/SNF complexes, as well as an appropriate chromatin conformation allowing the physical proximity between *SWINGN* and the regulated loci, are necessary for this regulatory mechanism, as suggested by the absence of *SWINGN*-SMARCB1 crosstalk in other cell lines such as AT/RT or HCT116.

Intriguingly, besides GAS6 regulation, *SWINGN* affects SMARCB1 binding and transcriptional activation at additional distant gene loci, with a more prominent effect at proximal sites, where it is accompanied by a concomitant change in H3K27ac. This finding led us to propose that this lncRNA can influence the ability of the SWI/SNF complexes to regulate promoter activation, possibly by establishing contacts with multiple loci. This hypothesis is supported by the analysis of the genes subject to SMARCB1-*SWINGN* regulation, which not only include *GAS6*, but additional targets belonging to the same chromosomal band, suggesting that *SWINGN* enhancer locus extends its control to longer distances, as already demonstrated for other lncRNA-producing enhancers^48,49^. Intriguingly, some of the most relevant SMARCB1-*SWINGN* targets, such as *PDGFRB* and *COL1A1*, are located in different chromosomes. The detection of *SWINGN* enrichment at *PDGFRB* by ChIRP supports the notion that SMARCB1 and *SWINGN-*containing complexes participate in the regulation of these loci. We propose that a precise spatial chromatin organization around *SWINGN* enhancer region could bring together multiple distant genomic loci, allowing their co-regulation by a small number of *SWINGN* molecules. Some of our conclusions are supported by gene expression and ChIP-seq analyses upon *SWINGN* depletion, but applying ligation-free methods to capture DNA interactions at unforeseen distance or even between different chromosomes would greatly contribute to shedding light on the long-distance regulatory function of this lncRNA^50,51^.

The unveiled role of *SWINGN* in the establishment of its regulatory network provides an epigenetic signature for tumor progression linked to SMARCB1 alterations. While the global tumor-suppressor activity of SWI/SNF is well established, we have uncovered a subjacent oncogenic set of SMARCB1-*SWINGN* targets, including *GAS6, PDGFRB*, and *COL1A1*. In tumors such as lung squamous cancers, where the *SWINGN*-SWI/SNF axis is active and the regulatory connection is maintained, *SWINGN* may represent an interesting therapeutic target.

In conclusion, our study reveals that the SWI/SNF complexes can specifically bind lncRNAs such as *SWINGN*, which can influence their ability to drive activation of specific promoters. Furthermore, they suggest the possibility of a general mechanism in which SWI/SNF complexes cooperate with RNA to achieve transcriptional activation.

## Methods

### Cell culture, retroviral-lentiviral infection, and treatments

The following human cell lines were used for this study: BJ hTERT foreskin fibroblasts (kindly provided by Dr. J. Gil laboratory), IMR90 lung fibroblasts and HEK-293T (ATCC), H226 lung carcinoma cells, HCT116 colon adenocarcinoma and CHLA-06 rhabdoid tumor cells.

BJ hTERT, IMR90 and HEK-293T cell lines were cultured in DMEM medium (GIBCO), supplemented with 10% fetal bovine serum (GIBCO) and 1× penicillin/streptomycin (Lonza). H226 cells were maintained in RPMI-1640 medium (GIBCO), supplemented as described above. CHLA-06 were cultured in DMEM:F12 medium, complemented with 1× B-27, 20 µg/ml EGF and 20 µg/ml FGF (all GIBCO).

Cells were maintained at 37 °C in the presence of 5% CO_2_ and tested for mycoplasma contamination regularly, using the MycoAlert Mycoplasma Detection Kit (Lonza).

To generate OIS cell systems, viruses were first produced in HEK-293T cells transfected with 12 μg of either pLNC-ER:RAS or pLNCX (Empty) vectors (gift from Dr. Gil and described in the ref. ^52^, 6 μg of gag-pol plasmid and 3 μg of pVSVG vector.

Transfection reaction was carried out in opti-MEM medium (GIBCO) using Lipofectamine 2000 (Invitrogen), following manufacturer’s instructions. Forty-eight hours after transfection, filtered supernatant supplemented with 4 μg/ml polybrene (Santa Cruz), was used to transduce low passage BJ hTERT fibroblasts. Cells were selected with Neomycin-G418 (Sigma) at a final concentration of 400 μg/ml for at least one week.

For senescence induction studies, BJ hTERT ER:RAS fibroblasts were treated with 200 nM of 4-hydroxy-tamoxifen (4OHT) for six days replacing medium at day 3, unless specified otherwise in the text. Only fibroblasts cell lines with less than 20–25 passages were used for experiments.

For clustered regularly interspaced short palindromic repeat (CRISPR) activation studies, H226 cells were first engineered to stably express a nucleolytically inactive Cas9 protein (dCas9) fused to VP64 transcriptional activator (Addgene Plasmid #61425). Lentiviral production in HEK-293T cells and H226 infection were performed as previously described^53^. To induce *SWINGN* expression different single-guide RNAs (sgRNAs) were designed in the genomic region +1/+200 bp upstream the TSS of *SWINGN* long isoform. sgRNAs were then cloned into lentiviral plasmids expressing blue fluorescent protein (CRISPseq-BFP-backbone, Addgene Plasmid #85707) as described in the ref. ^54^. Three days after infection cells expressing high BFP levels were collected and RNA levels were analyzed by RT-qPCR.

Stable AT/RT cell lines with inducible re-expression of SMARCB1 were established as previously reported^38^. Briefly, CHLA-06 cells were transduced with pInducer-21-SMARCB1 or empty pInducer-21 lentiviral vectors, kindly provided by Charles Roberts’s lab. Seventy-two hours after transduction, GFP-positive cells were sorted and treated or not with doxycycline (1 μg/ml) for 48 h to induce SMARCB1 re-expression.

For exogenous GAS6 treatment, recombinant human GAS6 (rGas6; B&D system) was added to cell medium of IMR90 or H226 cells to a final concentration of 250 ng/ml and incubation was prolonged for 48 h.

For Actinomycin D (ActD) treatment, BJ hTERT ER:RAS cells were grown for different time points with a final concentration of 10 µg/ml of ActD solution (Sigma).

### RNAi and antisense silencing studies

For siRNA studies, cells were transfected once with a final concentration of 40 nM siRNA using Lipofectamine 2000 in opti-MEM medium, unless differently stated in the text. Scrambled siRNA (siRNA CTRL in the text) was used as transfection control. For antisense oligonucleotides (ASOs) approach, 50 nM of ASOs were transfected with Lipofectamine 3000 (Invitrogen) in optiMEM medium, according to standard procedures. CTRL ASO served as transfection control.

All siRNAs employed in this study were designed using BLOCK-iT™ RNAi Designer webpage (https://rnaidesigner.thermofisher.com/rnaiexpress/) and purchased from Sigma.

All *SWINGN*-targeting and CTRL ASOs were designed and synthesized by Ionis Pharmaceuticals. ASOs shown in this study were selected from a larger panel of oligonucleotides based on high levels of on-target inhibition and low levels of off-target and toxicity effect.

All siRNAs used in this study are listed in Supplementary Data 6.

### Nuclear/cytoplasm fractionation

3 × 10^6^ BJ cells were collected by trypsinization, divided into two tubes and spun down. Cell pellets were re-suspended in 500 μl of Lysis Buffer (10 mM Tris-HCl [pH 7.5], 140 mM NaCl, 1.5 mM MgCl2, 0.05% NP-40 [Sigma], supplemented with 1× cOmplete Protease Inhibitor Cocktail [Roche] and 20 U/ml RNAsin Ribonuclease Inhibitors [Promega]) and incubated on ice for 10 min. While the first cell pellet (representing total cell lysate) was kept for RNA extraction, the second one was slowly transferred on top of 500 μl of Lysis Buffer supplemented with 50% sucrose previously placed at the bottom of a clean tube. After centrifugation (13,000 rpm for 10 min at 4 °C), supernatant representing the cytoplasmic fraction, was carefully recovered using an insulin syringe without disturbing the pellet, representing the nuclear fraction. Nuclear pellet was re-suspended in 450 μl of Lysis Buffer and TRI reagent was added to all the fractions for RNA isolation, as described above.

### Cell proliferation and apoptosis assays

For proliferation assay, 1000 cells/well were plated in 96-well plates and cell proliferation was measured over 3 or 4 days with a CellTiter96 Aqueous Non-Radioactive Cell Proliferation Assay (MTS) Kit (Promega), following manufacturer’s advice. Four hundred and ninety nanomolar absorbance was measuared by spectrophotometry using the SPECTROstar Nano 96-well plate reader (BMG Labtech).

For clonogenicity assay, 500–1000 cells were plated in 6-well plates and grown for 8–10 days in normal medium. Cells were fixed with 0.5% glutaraldehyde for 15 min and stained with 0.1% Crystal Violet solution (Sigma) for 30 min.

To assess colony formation capacity, plates were air-dried and colonies were counted manually. To quantify cell density, cells were incubated with 500 μl of 10% acetic acid (Sigma) and collected in ELISA plates. Absorbance was measured by spectrophotometry at 570 nm in a SPECTROstar Nano equipment.

For BrdU incorporation assay, cells were labeled with 50 μM BrdU solution (BD Pharmingen) for 16–18 h. In case of senescent cells, OIS was previously induced for 5 days before starting BrdU assay. Then, cells were harvested by trypsinization and BrdU staining was performed using BrdU Flow Kits (BD Pharmingen), according to manufacturer’s instructions. Amount of BrdU incorporation was measured by flow cytometry using a FACSCalibur Cell Analyzer (BD Bioscience).

To measure apoptosis, cells were quickly detached using Accutase solution (Lonza) and kept on ice. Apoptosis was assayed by Annexin V and 7-AAD staining using the Apoptosis Detection Kit I (BD Biosciences) and FACSCalibur flow cytometer, following manufacturer’s recommendations.

Flow cytometry data were recorded by BD CellQuest program and analyzed using the FlowJo software.

### Xenograft model

1.5 × 10^6^ H226 cells transfected with *SWINGN*-specific or CTRL ASOs were re-suspended in 100 µl of complete medium and mixed with Matrigel Matrix (Corning) in a ratio of 1:1. The resultant mix was injected subcutaneously in the flank of 6–7-weeks-old female BALB/cA-Rag2^−/−^γc^−/−^ immunodeficient mice (*n* = 7 per condition). Tumor size was measured over 39 days at the indicated times in a blinded fashion using an electronic precision caliper. The tumor volume (*V*) was calculated using the formula: *V* = *π*/6 × width^2^ × length.

### Protein extraction and immunoblot analysis

Cells were lysed for 15 min in rotation at 4 °C using RIPA buffer (150 mM NaCl, 25 mM Tris HCl [pH 7.5], 2 mM EDTA, 0.1% sodium deoxycholate [Na-DOC], 0.1% SDS, 1% Triton X-100) supplemented with 1× cOmplete Protease Inhibitor Cocktail [Roche]. Lysed cells were centrifuged at max speed for 10 min at 4 °C and the insoluble pellet was discarded. Protein concentration was estimated by Pierce BCA Protein Assay Kit, using BSA curve as reference and according to manufacturer’s instructions. Proteins were separated on denaturing SDS-PAGE gels and transferred to a nitrocellulose membrane [Biorad] following standard procedures. Membranes were blocked using skim milk or BSA (VWR) and probed first for primary and then for HRP-conjugated secondary antibody. Western Lightening ECL-Plus (Perkin Elmer) was employed for chemiluminescence detection of proteins.

The list of antibodies used in this study can be found in Supplementary Data 6. Uncropped scans of the most important blots are supplied in the Source Data file.

### RNA extraction, RT-qPCR analysis, and ddPCR analysis

Total RNA was isolated using TRI reagent (Sigma), treated with DNase I (Invitrogen) and reverse-transcribed using the High-Capacity cDNA Reverse Transcription Kit (Applied Biosystem) with random hexamer primers following manufacturer’s instructions. For reverse-transcription of only poly-A (+) RNAs, DNAse I-treated total RNA was converted into cDNA using the SuperScript II Reverse Transcriptase Kit (Invitrogen) with 2.5 µM of Oligo(dT)^20^. The obtained cDNA was analyzed by quantitative PCR using iTaq Universal SYBR Green supermix (Bio-Rad) in a ViiA™ 7 Real-Time PCR System machine (Thermo-Fisher). All reactions were performed in triplicate or quadruplicate and *HPRT1* (Hypoxanthine Phosphoribosyl transferase 1) RNA levels were used for normalization, unless specified otherwise in Figure legend.

To evaluate the absolute number of *SWINGN* RNA molecules per cell, total RNA was isolated from 0.5 × 10^6^ BJ or H226 cells accurately counted using a Countess Automated Cell Counter (Thermo Fisher). RNA extraction and cDNA generation was performed as described above using 1 µg of RNA. Droplet digital PCR (ddPCR) analysis was performed using the QX200™ Droplet Digital PCR System (Bio-Rad) with the 20 μl reaction mixtures containing 0.9 μM primers, 0.25 μM probes, 1× ddPCR Supermix for Probes No dUTP (Bio-Rad) and 30 ng of cDNA as final concentration. PCR amplification settings were optimized using different temperatures and final assay was performed with the following cycling conditions: 10 min at 95 °C; 40 cycles of 30 sec at 94 °C and 60 s at 56 °C; and 1 cycle of 10 min at 98 °C with a 2 °C/s ramp rate. Positive droplet populations were separated from negative droplets and quantified automatically with QuantaSoft droplet reader software (Bio-Rad) as copies/μl. Probes targeting long or short *SWINGN* isoforms were designed in the regions amplified by set#1 or set#2 primer sets, respectively (details in Supplementary Fig. 3). Probes carrying a 5′-FAM (Fluorescein) fluorophore, a 3′-IBFQ (Iowa Black Quencer) and an internal ZEN quenchers, were synthesized by IDT.

All qPCR primer and ddPCR probe sequences are provided in Supplementary Data 6.

### RNA sequencing and data analysis

For RNA-seq of BJ cells depleted or not of *SWINGN*, total RNA was isolated and DNAse I-treated using Maxwell Simply RNA kit (Promega). ASO_LINC#3 was used to knockdown *SWINGN* expression. One microgram of quality-verified RNA was used for library preparation and sequenced on Illumina Nextseq 500 (75 bp single-end mode, 10 × 10^6^ reads/sample). Sequencing data were aligned to the genome assembly hg19 using STAR^55^ with default parameters. Differential expression analysis was carried out by using edgeR^56^ in R/Bioconductor and significant genes were selected applying the following filters: adjusted *p*-value < 0.01; |log_2_FC| > 0.5.

### RNA immunoprecipitation and sequencing (RIP-seq)

Native RNA immunoprecipitation was performed as previously described^2^ with minor modifications. Briefly, 1.5 × 10^7^ BJ fibroblasts were treated to induce senescence as indicated in the text, harvested and lysed. Nuclear extract were isolated and homogenized by dounce tissue grinder (pestle B), pre-cleared using Dynabeads Protein A or G beads and incubated with specific antibodies (specified in Supplementary Data 6) overnight. 40 µl of Protein A or G beads were used to recover antibody-protein complexes, which were then split into two fractions (for protein and RNA detection). To isolate proteins, beads were mixed in 2× Protein Loading Buffer supplemented with DTT, heated at 95°C for 10 min and loaded in SDS-PAGE gel for western blot analysis. To retrieve RNA, beads were re-suspended in 1 ml of TRI reagent and RNA extraction was performed as stated above.

For RIP-qPCR, equal volumes of Input and Immunoprecipitated RNA were treated with DNAse I and reverse-transcribed. qPCR results were normalized and represented as percentage of Input.

For UV-RIP assay, BJ fibroblasts were previously UV crosslinked at 1500 × 100 µJ/cm^2^ in a UVC500-Hoefer UV crosslinker

For RIP-seq analysis, SMARCB1 native RIP experiment was performed in biological triplicate. RNA quality was verified by Experion RNA analysis kit (Bio-Rad) and quantified by Qubit4 fluorometer (Invitrogen). Three hundred nanogram of Input and SMARCB1-IP RNA were used for library preparation while IgG-IP did not recover sufficient RNA for library preparation. RNA was then treated with DNAse I and ribosomal RNA was eliminated by Ribo-Zero rRNA Removal Kit (Illumina). RIP-seq libraries were prepared using the TruSeq Stranded Total RNA library prep Illumina kit to preserve the original RNA strand information by introducing a dUTP incorporation step in place of dTT during Second Strand Synthesis. RNA sequencing was performed on Illumina HiSeq at 33 × 10^6^ reads per sample, 50 bp single-end sequencing mode.

Sequencing data were aligned to the genome assembly hg19 using STAR with default parameters. RIP-seq analysis was carried out with RIPseeker package^57^ (version 1.18.0). RIPseeker’s main function (*ripSeek*) was applied to identify significantly enriched peaks comparing SMARCB1-IP and Input combined replicates, first performing probabilistic inference of RIP regions (*mainSeek*) then tested for significance by HMM (*seekRIP*). Correct bin size was determined automatically (based on Shimazaki cost function) and results from plus and minus strands were merged. Standard settings were modified to select transcripts with cut off values for significance (*Adj. p*-value ≤ 1 × e^−10^) and fold change (logOddScore > 1). To annotate enriched RIP-seq peaks, *useMart* and *getAnnotation* from biomaRt and ChIPpeakAnno Bioconductor packages were used to retrieve up-to-date Ensembl annotations. Then, *annotatePeakInBatch* command from ChIPpeakAnno was employed to efficiently annotate the predicted regions based on the Ensembl annotation while *getBM* command from biomaRt was used to assign gene biotype to each transcript. 1641 and 1511 enriched transcripts were identified in proliferating and senescent conditions, respectively. Biotype feature was employed to select protein coding vs noncoding RNAs, which include lincRNA, antisense, miscRNA, scaRNA, and miRNA categories. Transcripts labeled as pseudogenes were excluded from following RIP analyses but considered when comparing SMARCB1 ChIP and RIP data. For data representation purpose, *ripSeek* was run with no cut off filters and all RIP-seq peaks detected were visualized as a volcano plot (Fig. 1b).

### In vitro RNA pulldown assay

To analyze SMARCB1 capacity of RNA binding, 30 pmol of recombinant SMARCB1 C-terminal MYC/DDK-tagged protein (Origene - TP317885) were attached to 40 μl anti-FLAG M2 Magnetic Beads (Sigma) in Binding Buffer (150 mM NaCl; 50 mM Tris HCl [pH 7.5]; 1 mM EDTA; 05% NP-40; 10% glycerol and supplemented with protease inhibitors) for 2 h at 4 °C. Total RNA was extracted from BJ fibroblasts as described above, denatured by heating at 70 °C for 10 min and then cool down slowly to 4 °C to allow proper folding. One microgram of renatured RNA was added to beads-conjugated SMARCB1 protein at RT for 3 h along with RNAse inhibitors. RNA-protein complexes were washed four times with the same incubation buffer with a concentration of 300 mM NaCl. RNAs were eluted and extracted with TRI reagent and analyzed by RT-qPCR as previously described. An aliquot of starting RNA material was processed in parallel and used as input RNA for normalization. The position of the different primer sets spanning *SWINGN* sequence is illustrated in Supplementary Fig. 3B.

### Chromatin immunoprecipitation (ChIP)

H3K27ac ChIP qPCR was performed as previously described^29^.

SMARCB1 and histone marks ChIP was performed as described^58^ with some modifications. Briefly, 4 × 10^7^ BJ cells were double-crosslinked in 2 mM disuccinimidyl glutarate (DSG - Pierce) for 30 min and then 1% methanol-free formaldehyde was aggregated for 10 min at RT. Crosslink reaction was quenched with 0.125 M glycine and cells were centrifuged and washed with PBS. The cell pellet was then re-suspended in Lysis Buffer (10 mM Tris-HCl [pH 8], 85 mM KCl, 0.5% NP-40, supplemented with proteases inhibitors) and rotated for 10 min at 4 °C. Nuclear fraction was isolated by centrifugation and dissolved in RIPA-I Buffer (10 mM Tris-HCl [pH8], 1 mM EDTA [pH 8], 140 mM NaCl, 0.2% SDS, 0.1% sodium Na-DOC, supplemented with proteases inhibitors). Chromatin was sheared by sonication in a Bioruptor device for 45 cycles (30″ON-30″OFF), one volume of RIPA-II (10 mM Tris-HCl [pH 8], 1 mM EDTA [pH 8], 140 mM NaCl, 2% Triton X-100, 0.1% sodium Na-DOC) was added and insoluble chromatin was discarded after centrifugation at max speed. Cleared nuclear extract was incubated overnight at 4 °C with primary antibodies (listed in Supplementary Data 6), after saving 1% nuclear lysate as Input. The following day, 50 µl of Dynabeads Protein G beads where washed and mixed to antibody-hybridized nuclear extract for 1 h at 4 °C. Beads were extensively washed with the following wash buffers: RIPA-500 (10 mM Tris-HCl [pH 8], 1 mM EDTA [pH 8], 500 mM NaCl, 1% Triton X-100, 0.1% SDS); WB-I (20 mM Tris-HCl [pH 7.4], 2 mM EDTA [pH8], 150 mM NaCl, 0.1% SDS, 1% Triton X-100); WB-II (10 mM Tris-HCl [pH 8], 1 mM EDTA [pH 8], 250 mM LiCl, 1% Triton X-100, 0.7% Na-DOC); TET (10 mM Tris-HCl [pH 8], 1 mM EDTA [pH 8], 0.2% Tween-20). Beads were eventually eluted in Direct Elution Buffer (10 mM Tris-HCl [pH 8], 5 mM EDTA [pH 8], 250 mM NaCl, 0.4% SDS) and treated with RNAse A (Ambion) for 30′ at 37 °C together with the Input sample, previously adjusted in salt and SDS concentration. IP and Input samples were then decrosslinked by Proteinase K (NEB) incubation for 1 h at 37 °C and 4 h at 65 °C. DNA was isolated with SPRI beads (Ampure XP beads - Beckman Coulter), according to manufacturer’s instructions and quantified by Qubit. Correct chromatin fragmentation was evaluated by running decrosslinked Input samples on agarose gel.

For ChIP-seq analysis of cells depleted of *SWINGN*, BJ fibroblasts were transfected with CTRL or LINC#3 ASOs and cells were collected 36 h after transfection.

### ChIP sequencing (ChIP-seq) and data analysis

ChIP-seq libraries were prepared with at least 5 ng of DNA, following the protocol described in the ref. ^58^ including a final 0.65× SPRI beads clean-up for fragment size selection. Pooled ChIP-seq library concentration was measure by Qubit and mean DNA fragment size was assessed with a 4200 TapeStation Automated Electrophoresis System (Agilent Technologies). Multiplexed libraries were sequenced on an Illumina Nextseq500 platform in a 75 bp pair-end mode, with a depth of at least 20 × 10^6^ reads/sample. CTRL and *SWINGN*-knockdown ChIP-seq experiment was performed in biological duplicate.

ChIP-seq fastq files were aligned to the human reference genome (hg19), BAM files were sorted and PCR replicates were removed using bowtie2 (parameters:–no-discordant–no-mixed -X 1000–very-sensitive), PICARD and samtools (parameters: -q 15 -bh -F 1028)^59^. BedGraph and BigWig files were generated using bedtools^60^ and bedGraphToBigWig tools. ChIP-seq peaks were determined by performing MACS2 (version 2.1.0)^61^ peak calling with the following parameters: –bw 350 -q 0.01 without Input option. Peaks located in ENCODE blacklisted regions were excluded. ChIPpeakAnno package^62^ was used for gene assignment of MACS2 peaks in R/Bioconductor. For differential binding analysis of CTRL and *SWINGN*-knockdown ChIP-seq data, SMARCB1 and H3K27ac BAMs from two different replicates were merged and MACS2 was used to identify enriched peaks with the following cut offs: for H3K27ac ChIP, FDR < 0.001; for SMARCB1 ChIP, FDR < 0.0001. To identify more robust peaks, we only selected SMARCB1 peaks with fold change values (as in MACS2 output) > 2.

Metagene read densities and heat maps were generated using deepTools^63^ computeMatrix and selecting the center of MACS2 peaks as reference point or Gencode TSS annotation. Average read density was calculated by normalizing total read counts to the number of mapped reads to give reads per million mapped reads. Regions were sorted in descending order based on the mean value of the ChIP-seq signal per region and visualized using deepTools plotHeatmap. SMARCB1 and H3K27ac signals (as.bigwig) derived from merged BAM files of the two biological duplicates. Statistical significance was determined by unpaired Student’s *t*-test while Euclidian Distance was applied to compare difference between different samples.

SMARCB1 motif enrichment was assessed by using Multiple EM for Motif Elicitation (MEME) web platform, in particular MEME-ChIP tool (Version 5.0.2)^64^.

Chromatin state modeling was performed using the chromatin Hidden Markov Model (chromHMM) software as previously described^35,65^.

### Chromatin conformation capture (3C) analysis

Quantitative Chromosome Conformation Capture (3C-qPCR) assay was performed following a previously published protocol^66^ with minor modifications. Digestion efficiency was measured by qPCR quantification through multiple restrictions sites in undigested and digested samples, by using PCR primers that amplified across HindIII restriction sites. To analyze ligation products forward anchor primer was used in combination with forward primers designed across HindIII cutting sites over *SWINGN*/*GAS6* locus. Interaction frequencies were normalized to *HPRT* or intergenic region controls. Primer position is depicted in Supplementary Fig. 4E and their sequence can be found in Supplementary Data 6.

### ChIRP assay

ChIRP assay has been performed in BJ fibroblasts by using crosslinked nuclear extract from 9 × 10^7^ cells, as previously described^67^. Briefly, BJ cells were crosslinked in 1% formaldehyde and then lysed using Lysis Buffer (50 mM Tris HCl [pH 7], 10 mM EDTA, 1% SDS). Cell extract was sonicated in a Bioruptor device for 15 cycles (30″ON/45″OFF) and centrifuged at max speed to eliminate insoluble chromatin. One percent of cleared extract was saved as input while the remaining material was diluted with Hybridization Buffer (15% formamide, 500 mM NaCl, 1 mM EDTA, 0.5% SDS, supplemented with protease and RNAse inhibitors), incubated with specific probes and rotated O/N at RT. The following day 400 µl of Streptavidin magnetic beads (Dynabeads MyOne Streptavidin C1 - Thermo Fisher) were added to each pulldown condition and incubated 4 h at RT in rotation. Beads were then washed five times with Wash Buffer (2× saline-sodium citrate [SSC], 0.5% SDS) while elution was carried out using PK buffer (100 mM NaCl, 1 mM EDTA, 0.5% SDS, 10 mM Tris HCl [pH 7] or [pH 8] for RNA or DNA, respectively). 10% of ChIRP samples was used to analyze RNA enrichment by RT-qPCR after standard RNA extraction while the remaining material was employed to amplify selected DNA regions by qPCR. A pool of three different biotinylated oligonucleotides was used to pull down *SWINGN* RNA while two probes targeting *CONCR* lncRNA and two recognizing *LacZ* gene were used as independent controls. A total amount of 300 pm of probe was used in each reaction (100 pm of each *SWINGN* probe or 150 pm of CONCR and LacZ biotinylated oligos). All biotinylated probes were purchased from IDT and listed in Supplementary Data 6.

### Gene ontology and pathway analysis

To identify enriched molecular pathways associated with differences in gene expression, Ingenuity Pathway Analysis (IPA) (http://www.ingenuity.com/) was performed using filtered differentially expressed genes as indicated in the text. This analysis was implemented with Gene Ontology (GO) functional enrichment analysis, which was carried out through ToppFun web application, part of the ToppGene portal^68^. Representative significantly-enriched categories were selected with a Benjamini-Hochberg corrected false discovery rate (FDR B&H) threshold of 0.05.

### Expression analysis in tumor samples

Transcriptomic data (aligned with hg38 genome assembly) were obtained from the TCGA Data Portal (https://tcga-data.nci.nih.gov). *SWINGN* expression levels were evaluated in those cancer types presenting at least five normal and five tumor samples. Log_2_FPKM values were used for differential expression analysis, which was carried out with LIMMA^69^.

For correlation studies, log_2_FPKM gene expression values in TCGA data of different cancer types were used. The distribution of TCGA RNA-seq data for the genes of interest was evaluated by Shapiro-Wilk normality test and resulted not normal. Therefore, Spearman correlation was used to compare expression data. For logarithmic representation, a pseudocount was added representing the lowest FPKM expression value for each dataset.

To analyze *SWINGN* oncogenic signature, filtering was applied to select genes with affected SMARCB1 and H3K27ac binding upon lncRNA depletion, as well as highly changing in gene expression (|log_2_FC| > 1). The expression of this gene subset was evaluated in lung squamous carcinoma (LUSC) TCGA dataset to generate an expression matrix across the 552 LUSC samples (49 normal and 503 tumors). Principal component analysis followed by k-means algorithm was used to identify the different clusters. Silhouette function was used to determine the optimal number of clusters before applying the k-means method. Average gene expression for each sample was calculated separately for each cluster and represented as box plots.

All the analyses were performed in R/Bioconductor and statistical significance was determined by unpaired Student’s *t*-test.

### Statistical analysis

Experimental data were represented as mean ± standard deviation of at least three biological replicates (unless specified otherwise in Figure legends) and significance was determined by two-tailed unpaired Student’s *t*-test using GraphPad software. Significant *p*-values were summarized as follows: not significant (ns); *p*-value < 0.05 (*); *p*-value < 0.01 (**); *p*-value < 0.001 (***).

### Reporting summary

Further information on research design is available in the Nature Research Reporting Summary linked to this article.

## Supplementary information

Supplementary Information

Peer Review

Reporting Summary

Description of Additional Supplementary Files

Supplementary Data 1

Supplementary Data 2

Supplementary Data 3

Supplementary Data 4

Supplementary Data 5

Supplementary Data 6

## Data Availability

A reporting summary for this Article is available as a Supplementary Information file. RIP-seq, ChIP-seq and RNA-seq data reported that support the findings of this study have been deposited in Gene Expression Omnibus (GEO) repository under the accession number GSE128327. The raw data (including uncropped western blot images) underlying Figs. 1E, 3D–H, 4B–H, 5J, K, 6B–E and Supplementary Figs. 1A–E, I, J, 3H, 4A, C, G, 5A, B, D, 6A, E, I, 7A, B, E, 9B–D are provided as a source data file. All the other data supporting the findings of this study are available from the corresponding author upon reasonable request.

## Source Data

Source Data
