# Supplementary material for: A lncRNA-SWI/SNF complex crosstalk controls transcriptional activation at specific promoter regions
Source: Nat Commun. 2020 Feb 18;11:936. doi: 10.1038/s41467-020-14623-3 (PMC7028943; doi:10.1038/s41467-020-14623-3)
Supplement: Supplementary file 1 — Supplementary Information [file 41467_2020_14623_MOESM1_ESM.pdf]

A lncRNA-SWI/SNF complex crosstalk controls transcriptional activation at specific promoter regions

Grossi et al.

Supplementary Information

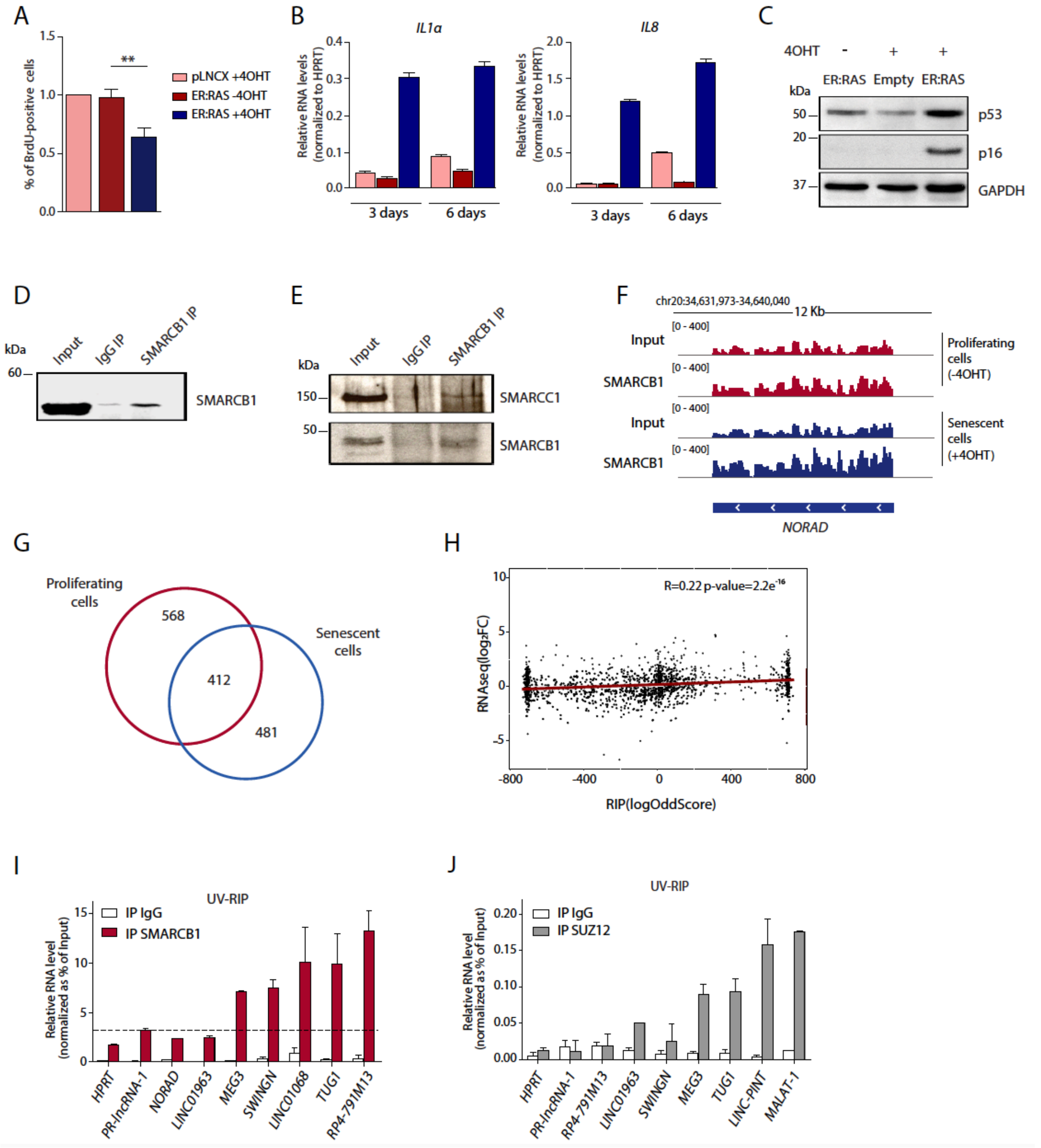

Supplementary Figure 1, related to Figure 1.

SMARCB1 interacts with specific RNAs upon senescence induction

(A) BrdU assay measuring growth arrest of BJ ER:Ras fibroblasts upon 4OHT treatment compared to untreated ER:Ras and BJ infected with pLNCX control vector.

(B) RT-qPCR analysis of *IL1α* and *IL8* mRNA levels after 3 or 6 days of 4OHT administration in BJ ER:Ras and pLNCX cells.

(C) Western blot showing p53 and p16 levels after 3 or 6 days of 4OHT administration in BJ ER:Ras and pLNCX cells.

(D) Western blot of Input and SMARCB1 IP extracts of RIP experiment (proliferating BJ cells)

(E) Co-immunoprecipitation experiment of SMARCB1 in BJ proliferating cells

(F) Example tracks of Input and SMARCB1 IP at NORAD genomic locus in proliferating (red color, -4OHT) and senescent (blue color, +4OHT) BJ cells.

(G) Venn diagram showing the overlap between protein-coding genes significantly enriched in SMARCB1 IP in proliferating (red-colored) and senescent conditions (blue-colored). Significantly enriched protein coding genes were selected based on the following filters: *Adj. p-value*$\leq 1e^{-10}$; logOddScore>1. *P-value* refers to posterior probability value.

(H) Correlation plot showing the logOddScore values (RIP on x axis, positive or negative for enrichment in +4OHT or -4OHT conditions, respectively) and logFC (log fold change) expression levels obtained from differential expression analysis (RNAseq) of proliferating/senescent input samples for each gene in RIP-seq analysis. Correlation *p-value* is calculated using a t-distribution.

(I) SMARCB1 UV-RIP assay followed by RT-qPCR of a set of known and uncharacterized lncRNA candidates. RNA enrichment in SMARCB1 IP was calculated as percentage of input, using IgG IP as immunoprecipitation control. *SWINGN* candidate was amplified with primer set#2 (details in Suppl. Figure 3B). *HPRT* and *p53-regulated lncRNA-1* (*PR-lncRNA-1*) RNAs were amplified as further negative controls. n=2 biological replicates; a representative experiment is shown as mean ± SD.

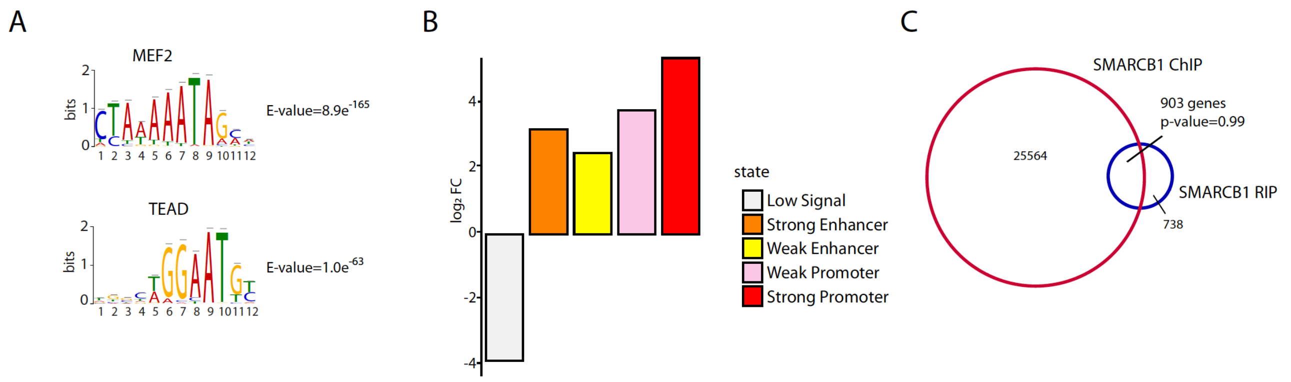

Supplementary Figure 2, related to Figure 2.

SMARCB1 preferentially binds at active, transcribed genomic regions.

(A) Third and fourth most enriched consensus motifs, as defined by MEME-ChIP motif analysis for the enriched peaks (46809) found in SMARCB1 ChIP-seq experiment in BJ cells.

(B) Distribution of the different chromatin states generated by ChromHMM for the SMARCB1 binding map represented as fold change in respect to the distribution in BJ total genome.

(C) Venn diagram representing the overlap between genes presenting a SMARCB1 ChIP peak (26467) and a random selection of genes detected in RIP input samples (1641). Significance (upper cumulative *p-value*) has been calculated by hypergeometric test.

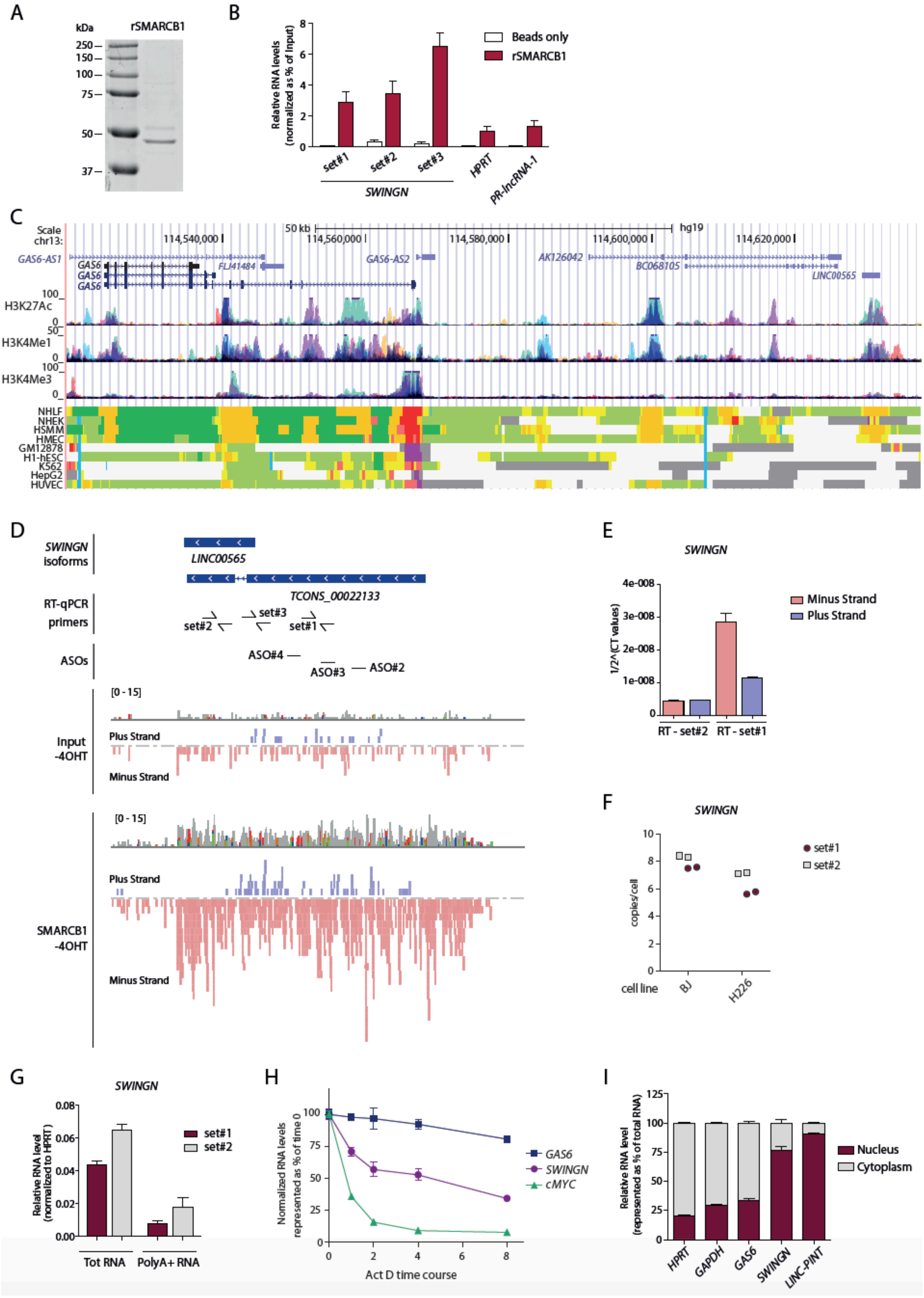
A
kDa
rSMARCB1
250
150
100
75
50
37
B
Relative RNA levels
(normalized as % of Input)
Beads only
rSMARCB1
set#1
set#2
set#3
SWINGN
HPRT
PR-lncRNA-1
C
Scale
chr13:
50 kb
hg19
114,540,000
114,560,000
114,580,000
114,600,000
114,620,000
GAS6-AS1
GAS6
FLJ41484
GAS6-AS2
AK126042
BC068105
LINC00565
H3K27Ac
H3K4Me1
H3K4Me3
NHLF
NHEK
HSMM
HMEC
GM12878
H1-hESC
K562
HepG2
HUVEC
D
SWINGN
isoforms
LINC00565
TCONS_00022133
RT-qPCR
primers
set#1
set#2
set#3
ASOs
ASO#4
ASO#3
ASO#2
[0 - 15]
Input
-4OHT
Plus Strand
Minus Strand
SMARCB1
-4OHT
E
SWINGN
1/2^(CT values)
4e-008
3e-008
2e-008
1e-008
Minus Strand
Plus Strand
RT - set#2
RT - set#1
F
SWINGN
copies/cell
set#1
set#2
cell line
BJ
H226
G
SWINGN
Relative RNA level
(normalized to HPRT)
0.08
0.06
0.04
0.02
0.00
set#1
set#2
Tot RNA
PolyA+ RNA
H
Normalized RNA levels
represented as % of time 0
GAS6
SWINGN
cMYC
Act D time course
I
Relative RNA level
(represented as % of total RNA)
Nucleus
Cytoplasm
HPRT
GAPDH
GAS6
SWINGN
LINC-PINT

Supplementary Figure 3, related to Figure 3.

***SWINGN* is a SWI/SNF-interacting lncRNA**

(A) Chromatin landscape at *SWINGN*/*GAS6* locus based on public data available through UCSC portal. Upper tracks show H3K27ac, H3K4me1, H3K4me3 public ChIP-seq data generated by ENCODE consortium for seven different cell lines. Lower track shows chromatin state segmentation by HMM (Hidden Markov Model) by ENCODE/Broad Institute consortium for each of nine different cell lines.

(B) Analysis of strand-specific sequencing reads of SMARCB1 and Input RIP-seq raw data at *SWINGN* locus in BJ proliferating cells, with the two sets of RT-qPCR primers and ASOs positions depicted in the top panel.

(C) qPCR analysis of minus- (pink) and plus- (purple) specific cDNA of BJ cells generated by strand-specific RT, using fwd#1/2 and rev#1/2 primers, respectively; strand-specific cDNA was then amplified by qPCR using set#1 primer pair. Graph shows mean ± SD of experimental replicates.

(D) Quantification of the number of molecules per cell of *SWINGN* by droplet digital PCR in BJ and H226 cells. Different primer sets have been used to amplify *SWINGN* transcripts.

(E) qPCR analysis of cDNA generated from total RNA (random primers-based RT) or polyA+ RNA (dT-based RT) of proliferating BJ cells. Both primer sets were tested and normalized to HPRT values.

(F) Actinomycin D assay analyzing *SWINGN* half-life along a time course (x axis) in BJ cells. *GAS6* and *cMYC* mRNAs were used as long and short half-life controls, respectively. RNA levels were represented as percentage of time 0. Similar results were obtained in IMR90 cells (data not shown). Graph shows a representative experiment (n=2).

(G) RT-qPCR analysis of nuclear/cytoplasm RNA fractionation of BJ proliferating cells. *HPRT*, *GAPDH* and *GAS6* mRNAs were analyzed as cytoplasmic-enriched controls while *LINC-PINT* as nuclear-enriched RNA species. Graph shows a representative experiment (n=2).

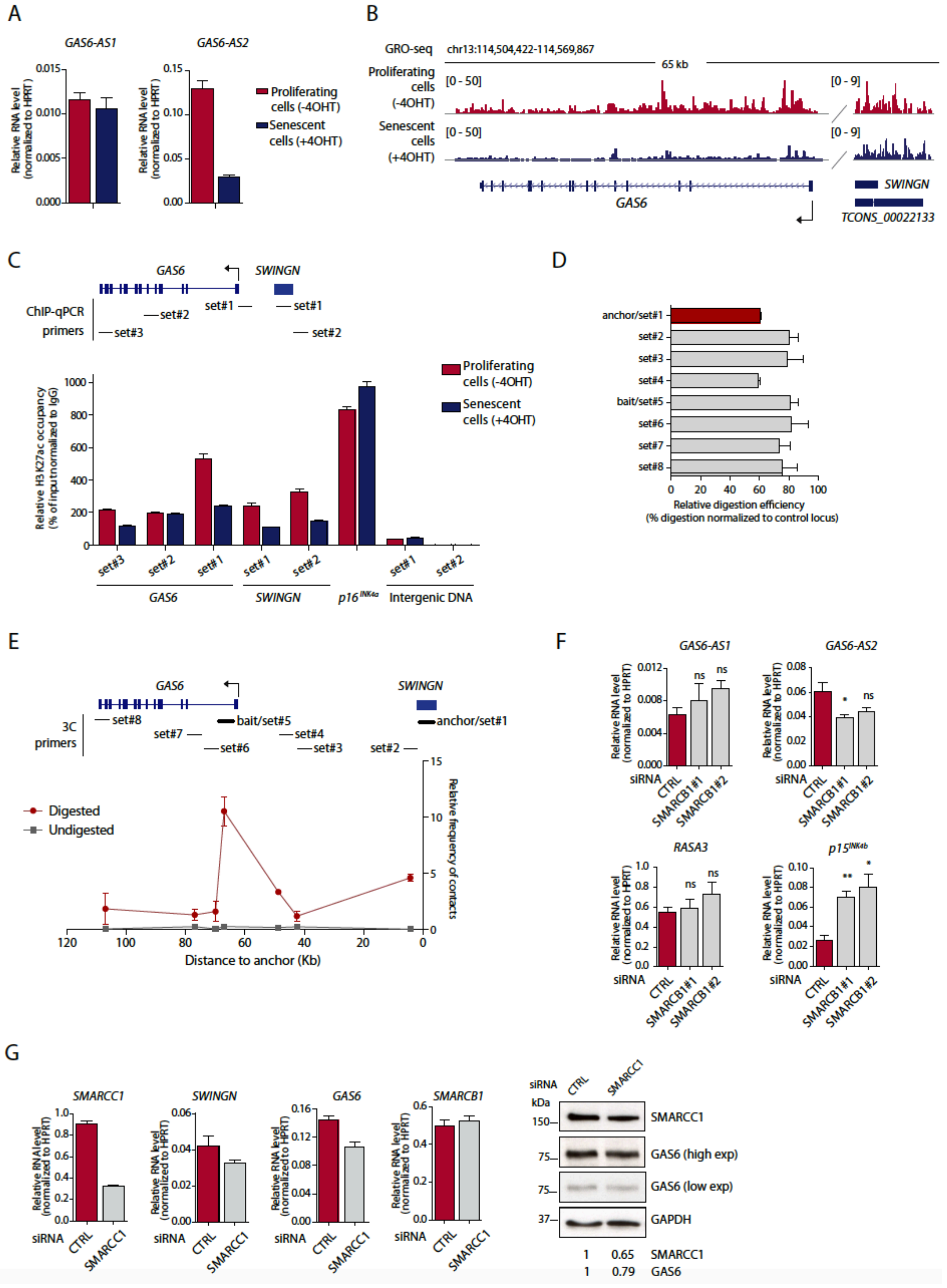
A
GAS6-AS1
GAS6-AS2
Relative RNA level (normalized to HPRT)
Proliferating cells (-4OHT)
Senescent cells (+4OHT)
B
GRO-seq
chr13:114,504,422-114,569,867
65 kb
Proliferating cells (-4OHT)
Senescent cells (+4OHT)
[0 - 50]
[0 - 9]
SWINGN
GAS6
TCONS_00022133
C
GAS6
SWINGN
ChIP-qPCR primers
set#1
set#2
set#3
Relative H3K27ac occupancy (% of input normalized to IgG)
Proliferating cells (-4OHT)
Senescent cells (+4OHT)
p16INK4a
Intergenic DNA
D
anchor/set#1
bait/set#5
Relative digestion efficiency
(% digestion normalized to control locus)
E
3C primers
set#8
set#7
set#6
set#4
set#3
set#2
Digested
Undigested
Distance to anchor (Kb)
Relative frequency of contacts
F
RASA3
p15INK4b
siRNA
CTRL
SMARCB1#1
SMARCB1#2
ns
G
SMARCC1
SWINGN
GAS6
SMARCB1
kDa
SMARCC1
GAS6 (high exp)
GAS6 (low exp)
GAPDH
1 0.65 SMARCC1
1 0.79 GAS6

Supplementary Figure 4, related to Figure 3.

***SWINGN* is transcribed from a *GAS6* enhancer locus**.

(A) RT-qPCR analyses of proliferating (-4OHT) and senescent (+4OHT) BJ fibroblasts.

(B) Example tracks of GRO-seq data (generated by R. Agami's lab) at *GAS6* locus comparing proliferating (red color, -4OHT) and senescent (blue color, +4OHT) TIG3 human lung fibroblasts.

(C) ChIP-qPCR analysis of H3K27ac occupancy at *SWINGN*/*GAS6* locus in proliferating (-4OHT) or senescent (+4OHT) BJ cells. Primer positions are represented in the scheme above. *p16* promoter and intergenic DNA regions were used as positive and negative controls, respectively. H3K27ac IP signal was represented as percentage of input and normalized on IgG IP signal. Graph shows mean ± SD of experimental replicates.

(D) Evaluation of digestion efficiency in BJ fibroblasts for the different primer sets spanning *SWINGN*/*GAS6* locus used for 3C analysis.

(E) (Upper panel) Schematic representation of primer set position. (Bottom panel) 3C assay followed by qPCR in BJ proliferating cells. Signal from HindIII-digested DNA was compared to undigested control. Data represent the average of three independent replicates

(F) RT-qPCR analyses of BJ proliferating cells depleted or not of SMARCB1 by siRNA double transfection.

(G) (Left) RT-qPCR analysis of BJ fibroblasts siRNA-depleted of *SMARCC1*. Graphs show mean ± SD of two independent experiments. (Right) Western blot analysis of BJ cells depleted of *SMARCC1*. Western blot image of a representative image of two independent experiments.

Significance was determined by two-tailed Student's t-test and summarized as follows: not significant (ns); * <0.05; ** <0.01; *** <0.001.

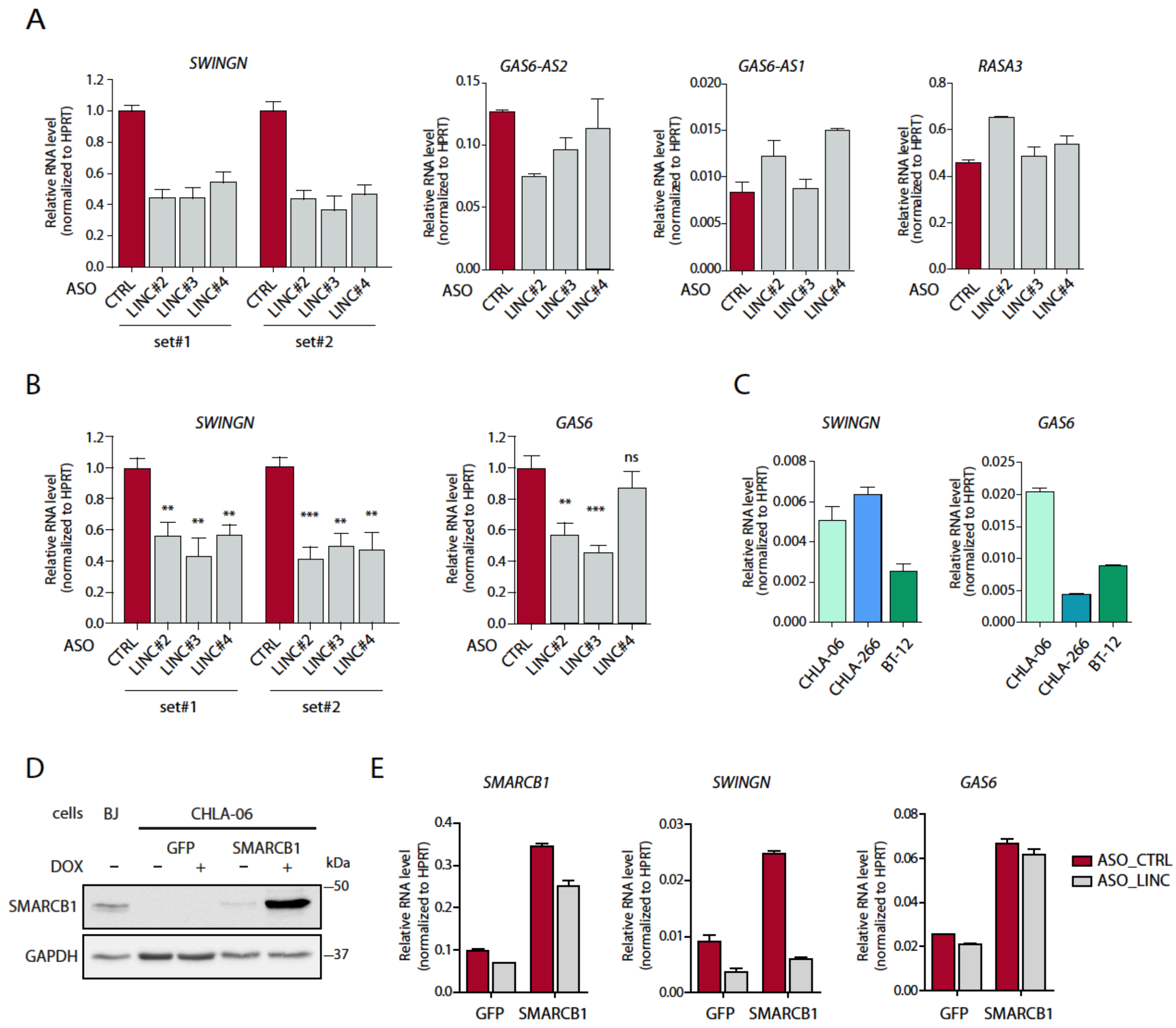

Supplementary Figure 5, related to Figure 3.

***SWINGN* controls the expression of *GAS6* gene through SMARCB1**

(A) RT-qPCR analysis of proliferating BJ fibroblasts depleted of *SWINGN* using three different ASOs. Two different primer sets (depicted in Suppl. Figure 3B) were used to detect *SWINGN* transcript. Graphs show mean ± SD of three independent experiments. For *SWINGN* expression RNA levels were normalized to ASO_CTRL values.

(B) RT-qPCR analysis of proliferating IMR90 fibroblasts depleted of *SWINGN* using three different ASOs. Two different primer sets (depicted in Suppl. Figure 3B) were used to detect *SWINGN* transcript. Graphs show mean ± SD of three independent experiments. RNA levels were normalized to ASO_CTRL values.

(C) RT-qPCR analysis of three different AT/RT cell lines. Primer set#1 was used to detect *SWINGN* transcript.

(D) Western blot analysis of BJ fibroblasts and CHLA-06 cells stably transduced with an empty or a SMARCB1 vector and treated +/- doxycycline (DOX) treatment.

(E) RT-qPCR analysis of empty or SMARCB1 CHLA-06 cells treated with doxycycline in control (ASO_CTRL) and *SWINGN* knockdown (ASO_LINC) conditions. *SWINGN* depletion was achieved using ASO LINC#3. Graph shows mean ± SD of experimental replicates.

Significance was determined by two-tailed Student's t-test and summarized as follows: not significant (ns); * <0.05; ** <0.01; *** <0.001.

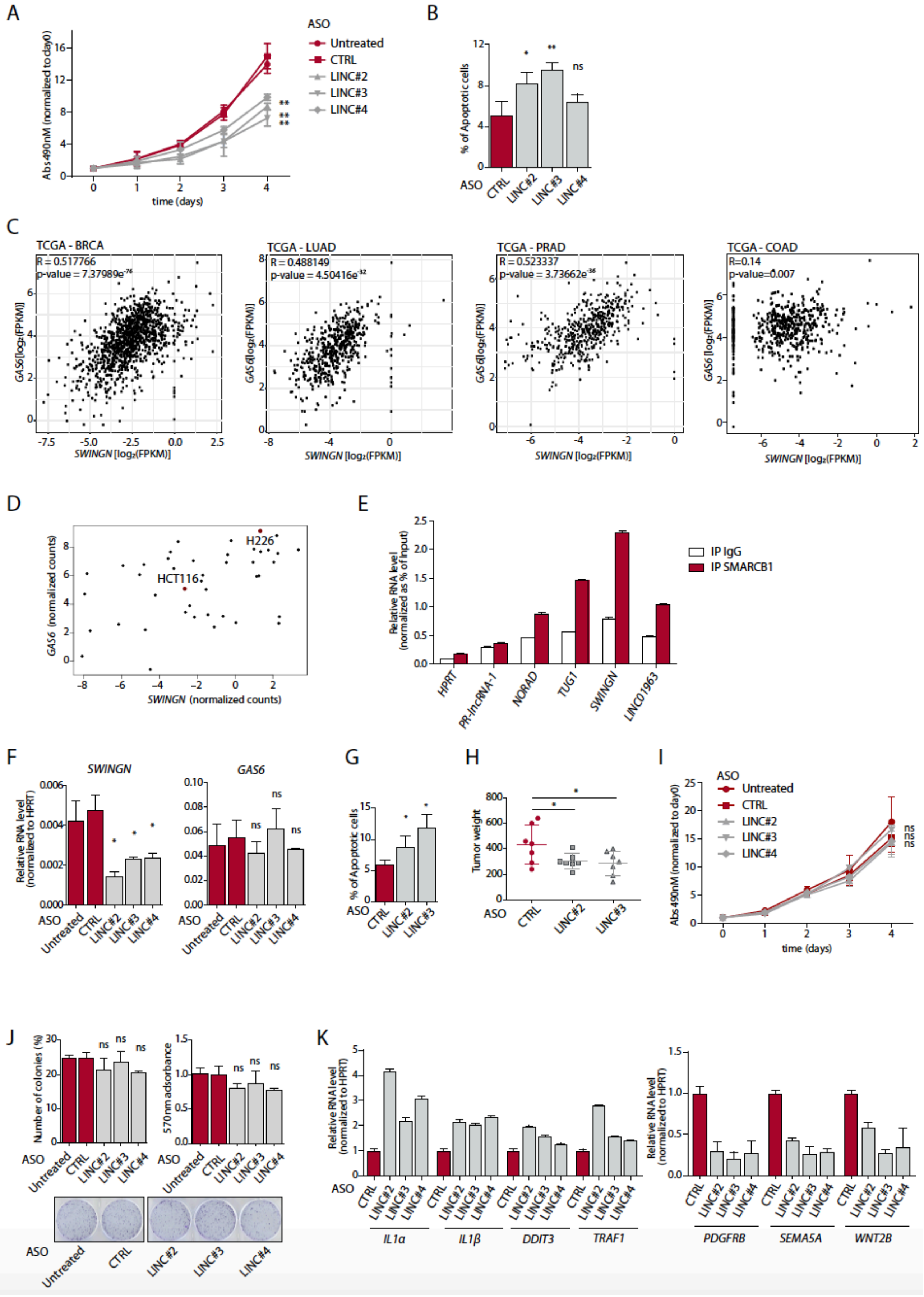

Supplementary Figure 6, related to Figure 4.

***SWINGN*-mediated G*AS6* regulation is relevant in cancer progression**

(A) MTS proliferation assay of control and *SWINGN*-depleted BJ cells. Absorbance values were normalized to day 0. Graph shows mean ± SEM of three independent experiments.

(B) Apoptosis assay measured by annexin V staining of control and *SWINGN*-depleted IMR90 cells. Graph shows mean ± SD of two independent experiments.

(C) Correlation plots showing *SWINGN* and *GAS6* expression levels (FPKM + pseudocount values) in breast carcinoma (BRCA; n=837), lung adenocarcinoma (LUAD; n=488), prostate adenocarcinoma (PRAD; n=374) and colon adenocarcinoma (COAD; n=157) from the TCGA database. Correlation *p-values* are calculated using a t-distribution.

(D) *SWINGN* and *GAS6* expression levels (normalized counts) across different cell lines of the Cancer Cell Line Encyclopedia (CCLE).

(E) SMARCB1 native RIP followed by RT-qPCR of lncRNA candidates in H226 cells. RNA enrichment was calculated as percentage of input, using IgG IP as immunoprecipitation control. *SWINGN* was amplified with primer set#2. *HPRT* and *PR-lncRNA-1* RNAs are negative controls.

(F) RT-qPCR analysis of HCT116 colon adenocarcinoma cells depleted of *SWINGN*.

(G) Apoptosis assay measured by annexin V staining in H226 cells. Graph shows mean ± SD of two independent experiments.

(H) Tumor weight at the day of sacrifice (day 39) of xenograft models, generated by subcutaneous injection of control or *SWINGN*-depleted H226 cells in BALB/cA-Rag2−/−γc−/− mice (n=7 per group).

(I) MTS assay of control and *SWINGN*-depleted HCT116 cells at the indicated time points and normalized to day 0. Graph shows mean ± SEM of two independent experiments.

(J) Clonogenicity assay of control and *SWINGN*-depleted HCT116 cells. Experiment has been analyzed by counting number of colonies (left panel) and the total cell density (absorbance, right panel). Graph shows mean ± SD of two independent experiments, picture refers to a representative experiment.

(K) RT-qPCR validation in BJ cells of a selection of the most affected genes in the RNA-seq analysis performed in H226 cells by using three different ASOs. RNA levels were normalized to ASO_CTRL values.

Significance was determined by two-tailed Student's t-test and summarized as follows: not significant (ns); * <0.05; ** <0.01; *** <0.001.

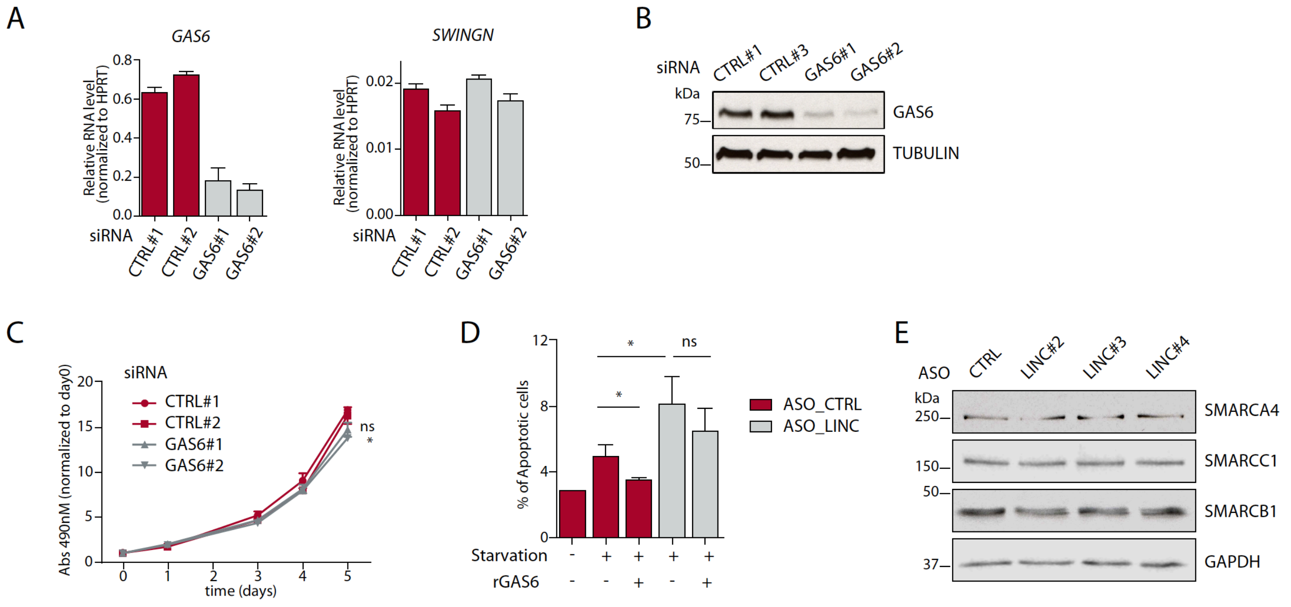

Supplementary Figure 7, related to Figure 5.

***GAS6* regulation does not account for full *SWINGN* function.**

(A) RT-qPCR analysis of BJ fibroblasts depleted of *GAS6* using two different siRNAs. Graphs show mean ± SD of three independent experiments.

(B) Western blot analysis of BJ cells depleted of *GAS6* using two different siRNAs. Picture refers to a representative experiment (n=2).

(C) MTS proliferation assay of control and *GAS6*-depleted BJ cells at the indicated time points. Absorbance values were normalized to day 0. Graph shows mean ± SEM of two independent experiments.

(D) Apoptosis assay measured by annexin V staining of control and *SWINGN*-depleted IMR90 cells. Cells underwent serum starvation and/or recombinant GAS6 (rGAS6) administration, as indicated. Graph shows mean ± SEM of two independent experiments.

(E) Western blot analysis of BJ cells depleted of *SWINGN* using three independent ASOs.

Significance was determined by two-tailed Student’s t-test and summarized as follows: not significant (ns); * <0.05.

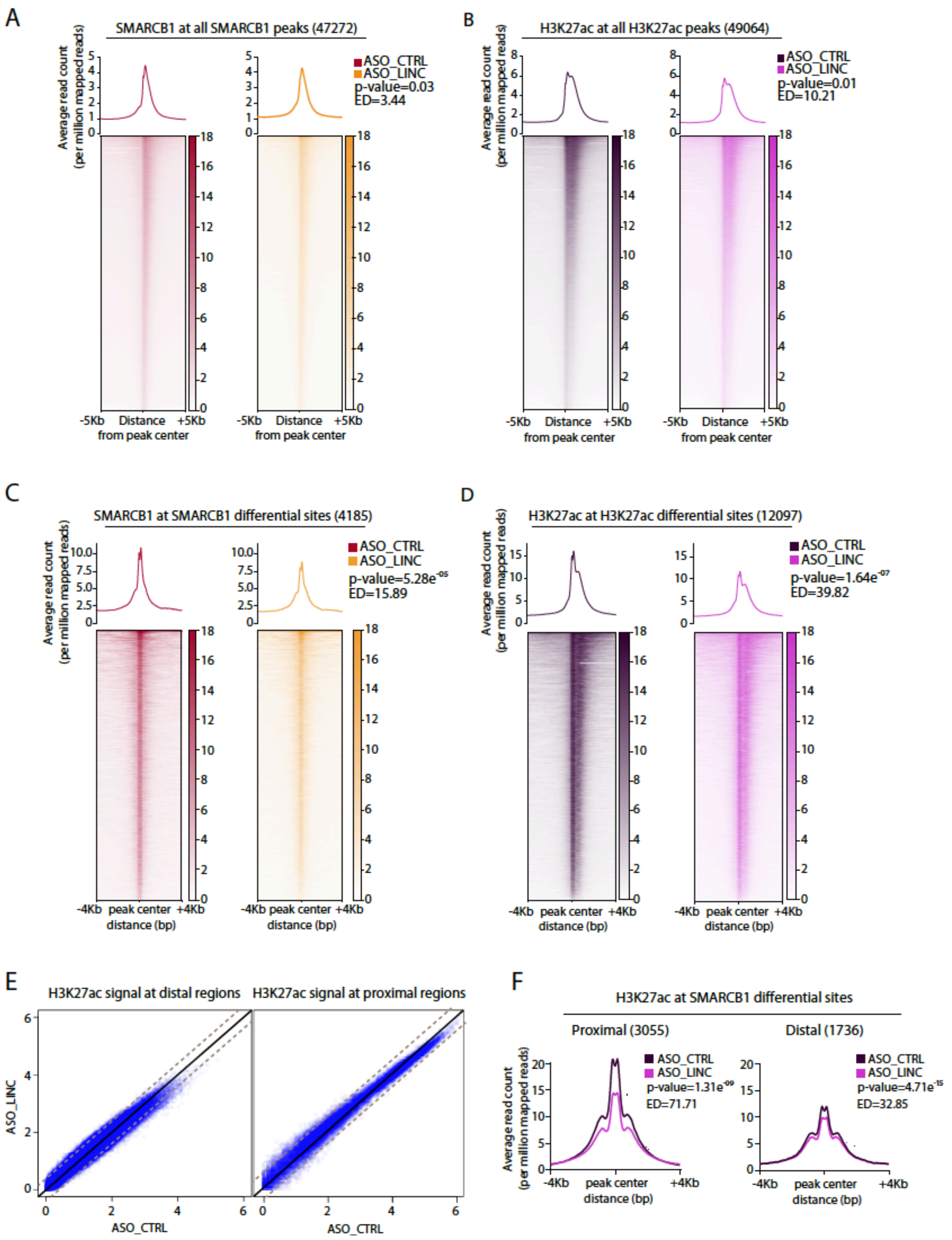

G

Cytoband	p-value	FDR B&H	Bonferroni	Genes from Input	Genes from Annotation
13q34	$3.55e^{-04}$	$2.38e^{-02}$	$2.38e^{-02}$	*COL4A1* *GAS6* *ADPRHL1*	60

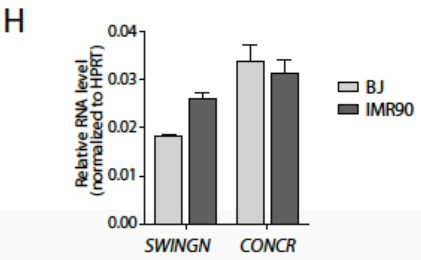

Supplementary Figure 8, related to Figure 5.

***SWINGN* regulates gene activation at additional loci by controlling SWI/SNF activity.**

(A) Heat maps and metagene plots of SMARCB1 occupancy on total SMARCB1 ChIP-seq peaks (47272) comparing ASO_CTRL (darker colored) and ASO_LINC (lighter colored) conditions. In (A), (B), (C), (D) and (F) panels significance has been calculated by t-test (represented as *p-value*) while difference between conditions has been measured by Euclidean Distance (ED).

(B) Heat maps and metagene plots of H3K27ac occupancy on total H3K27ac ChIP-seq peaks in ASO_CTRL (darker colored) and ASO_LINC (lighter colored) conditions.

(C) Side-by-side heat maps and metagene plots corresponding to Figure 5C.

(D) Side-by-side heat maps and metagene plots corresponding to Figure 5D.

(E) H3K27ac signal at total H3K27ac sites split by proximal-promoter (≤2Kb from TSS; right) and distal-enhancer (≥2Kb from TSS; left) regions comparing ASO_CTRL and ASO_LINC conditions.

(F) Metagene plots of H3K27ac signal at differential SMARCB1 regions (4791) identified as proximal-promoter (≤2Kb from TSS; right) and distal-enhancer (≥2Kb from TSS; left) sites comparing ASO_CTRL (darker colored) and ASO_LINC (lighter colored) conditions.

(G) Analysis of cytoband enrichment by applying Gene Set Enrichment Analysis to the gene list of 87 genes differentially expressed upon *SWINGN* knockdown in H226 cells and provided of a SMARCB1 or H3K27ac binding peak changing concordantly upon lncRNA depletion. Cutoff: FDR<0.01. FDR is represented as Benjamini-Hochberg or Bonferroni corrected.

(H) Expression levels of *SWINGN* and *CONCR* lncRNAs in BJ and IMR90 fibroblasts.

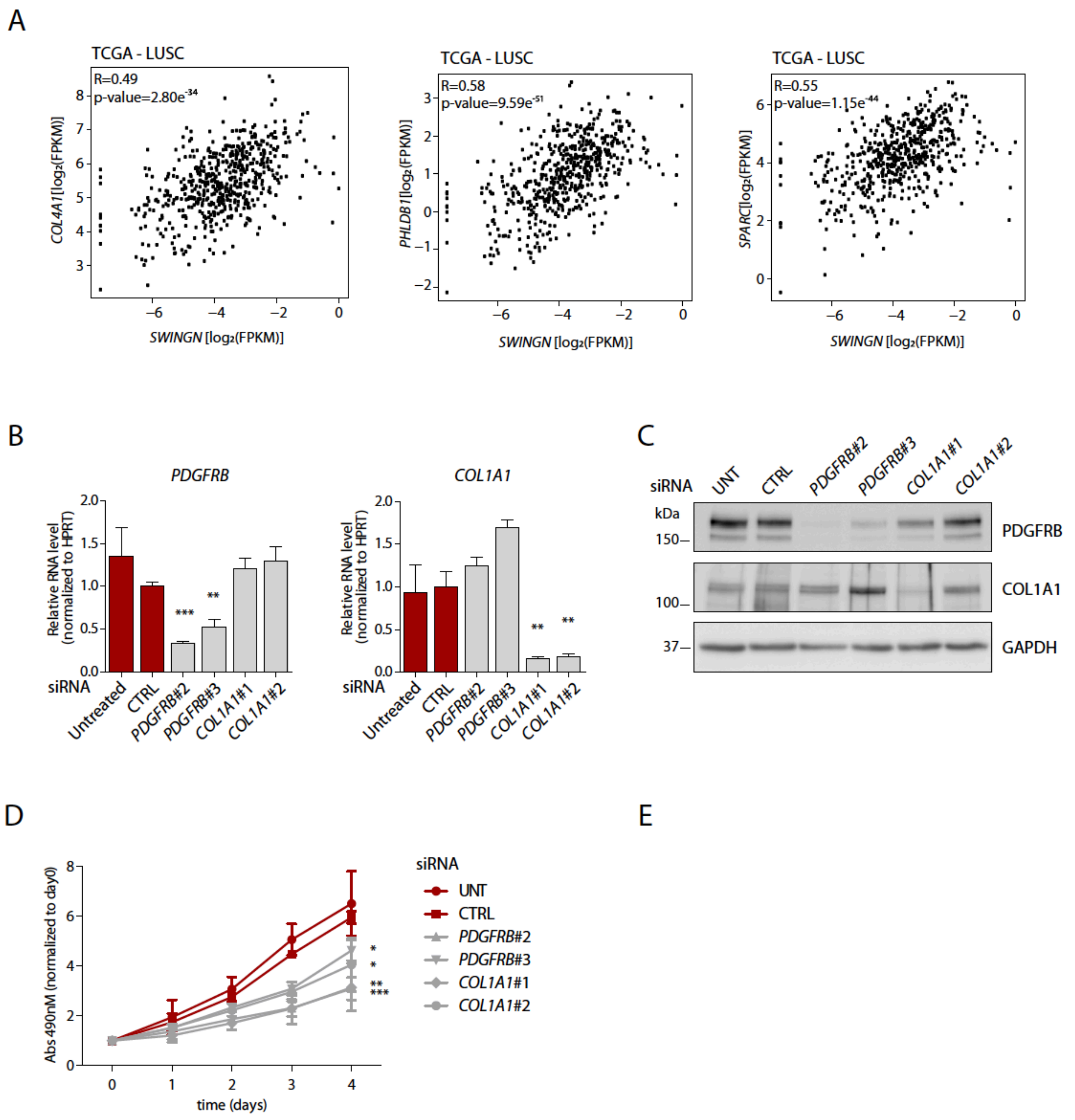

Supplementary Figure 9, related to Figure 6.

***SWINGN* controls a pro-oncogenic hub predictive of cancer status.**

(A) Scatter plots showing the correlation between *SWINGN* expression and the levels of other genes (*COL4A1, PHLDB1*, *SPARC)* belonging to the list in Figure 5F in lung squamous carcinoma dataset (LUSC; n=220) from the TCGA database. Values are expressed in FPKM + pseudocount. Correlation *p-values* are calculated using a t-distribution.

(B) RT-qPCR analysis of H226 cells depleted of *PDGFRB* or *COL1A1* using two different siRNAs for each target. Graphs show mean ± SD of three independent experiments. RNA levels were normalized to CTRL siRNA conditions.

(C) Western blot analysis of H226 cells depleted of *PDGFRB* or *COL1A1* using two different siRNAs for each target.

(D) MTS proliferation assay of H226 cells depleted of *PDGFRB* or *COL1A1* using two different siRNAs for each target at the indicated time points. Absorbance values were normalized to day 0. Graph shows mean ± SEM of three independent experiments. Significance has been calculated comparing each condition to CTRL siRNA sample.

Significance was determined by two-tailed Student's t-test and summarized as follows: not significant (ns); * <0.05; ** <0.01; *** <0.001.
