# Supplementary material for: A lncRNA-SWI/SNF complex crosstalk controls transcriptional activation at specific promoter regions
Source: Nat Commun. 2020 Feb 18;11:936. doi: 10.1038/s41467-020-14623-3 (PMC7028943; doi:10.1038/s41467-020-14623-3)
Supplement: Supplementary file 3 — Reporting Summary [file 41467_2020_14623_MOESM3_ESM.pdf]

nature research

Corresponding author(s): Maite Huarte

Last updated by author(s): Nov 8, 2019

Reporting Summary

Nature Research wishes to improve the reproducibility of the work that we publish. This form provides structure for consistency and transparency in reporting. For further information on Nature Research policies, see Authors & Referees and the Editorial Policy Checklist.

Statistics

For all statistical analyses, confirm that the following items are present in the figure legend, table legend, main text, or Methods section.

n/a	Confirmed	
☐	☒	The exact sample size (*n*) for each experimental group/condition, given as a discrete number and unit of measurement
☐	☒	A statement on whether measurements were taken from distinct samples or whether the same sample was measured repeatedly
☐	☒	The statistical test(s) used AND whether they are one- or two-sided<br>*Only common tests should be described solely by name; describe more complex techniques in the Methods section.*
☒	☐	A description of all covariates tested
☒	☐	A description of any assumptions or corrections, such as tests of normality and adjustment for multiple comparisons
☒	☐	A full description of the statistical parameters including central tendency (e.g. means) or other basic estimates (e.g. regression coefficient) AND variation (e.g. standard deviation) or associated estimates of uncertainty (e.g. confidence intervals)
☐	☒	For null hypothesis testing, the test statistic (e.g. *F*, *t*, *r*) with confidence intervals, effect sizes, degrees of freedom and *P* value noted<br>*Give P values as exact values whenever suitable.*
☒	☐	For Bayesian analysis, information on the choice of priors and Markov chain Monte Carlo settings
☒	☐	For hierarchical and complex designs, identification of the appropriate level for tests and full reporting of outcomes
☐	☒	Estimates of effect sizes (e.g. Cohen's *d*, Pearson's *r*), indicating how they were calculated

Our web collection on statistics for biologists contains articles on many of the points above.

Software and code

Policy information about availability of computer code

Data collection	Cancer transcriptomic data were obtained from the TCGA Data Portal (https://tcga-data.nci.nih.gov).
Data analysis	RNA sequencing data were aligned to the genome assembly hg19 using STAR. Differential expression analysis was carried out by using edgeR in R/Bioconductor.<br>Molecular pathways associated with difference in gene expression were identified by using Ingenuity Pathway Analysis (IPA) (http://www.ingenuity.com/).<br>RIP sequencing data were aligned to the genome assembly hg19 using STAR. RIP-seq analysis was carried out with RIPseeker package (version 1.18.0).<br>ChIP-seq fastq files were aligned to the human reference genome (hg19), BAM files were sorted and PCR replicates were removed using bowtie2, PICARD and samtools. BedGraph and BigWig files were generated using bedtools and bedGraphToBigWig tools.<br>ChIP-seq peaks were determined by performing MACS2 (version 2.1.0) peak calling.<br>ChIPpeakAnno package was used for gene assignment of MACS2 peaks in R/Bioconductor.<br>Metagene read densities and heat maps were generated using deepTools computeMatrix.<br>Motif enrichment was assessed by using MEME-ChIP tool (Version 5.0.2).

For manuscripts utilizing custom algorithms or software that are central to the research but not yet described in published literature, software must be made available to editors/reviewers. We strongly encourage code deposition in a community repository (e.g. GitHub). See the Nature Research guidelines for submitting code & software for further information.

Data

Policy information about availability of data

All manuscripts must include a data availability statement. This statement should provide the following information, where applicable:

- Accession codes, unique identifiers, or web links for publicly available datasets
- A list of figures that have associated raw data
- A description of any restrictions on data availability

All the high-throughput sequencing data (RNA-seq, RIP-seq and ChIP-seq) supporting the findings of this study have been deposited in GEO repository under the accession code GSE128139.

Field-specific reporting

Please select the one below that is the best fit for your research. If you are not sure, read the appropriate sections before making your selection.

☒ Life sciences ☐ Behavioural & social sciences ☐ Ecological, evolutionary & environmental sciences

For a reference copy of the document with all sections, see nature.com/documents/nr-reporting-summary-flat.pdf

Life sciences study design

All studies must disclose on these points even when the disclosure is negative.

Sample size	All the experiments have been performed in biological triplicate unless specified otherwise in figure legend. In xenograft studies n=7 per condition.
Data exclusions	Outliers (identified by GraphPad) were removed when necessary.
Replication	We were able to replicate the findings in this study.
Randomization	In xenograft experiments, mice were randomly assigned to control or treated groups.
Blinding	Investigators were blinded during tumor measurements.

Reporting for specific materials, systems and methods

We require information from authors about some types of materials, experimental systems and methods used in many studies. Here, indicate whether each material, system or method listed is relevant to your study. If you are not sure if a list item applies to your research, read the appropriate section before selecting a response.

Materials & experimental systems

n/a	Involved in the study	
☐	☒	Antibodies
☐	☒	Eukaryotic cell lines
☒	☐	Palaeontology
☐	☒	Animals and other organisms
☒	☐	Human research participants
☒	☐	Clinical data

Methods

n/a	Involved in the study	
☐	☒	ChIP-seq
☐	☒	Flow cytometry
☒	☐	MRI-based neuroimaging

Antibodies

Antibodies used

GAPDH ab9484 Abcam
HRP-conjugated GAPDH (14C10) 3683 CST
αTUBULIN T6199 Sigma
p53 (DO-1) sc-126 Santa Cruz
p16 554079 BD Pharmingen
SMARCB1/SNF5 ab58209 Abcam
SMARCC1/BAF155 (H-76) sc-10756 Santa Cruz
SMARCC1/BAF155 (DXD7) sc-32763 Santa Cruz
GAS6 (A-9) sc-376087 Santa Cruz
HRP-conjugated mouse IgG 7076S CST
HRP-conjugated rabbit IgG 7074S CST
Normal Mouse IgG sc-2025 Santa Cruz
Normal Rabbit IgG 2729S CST

	SMARCB1/SNF5 ab12167 Abcam<br>H3K27ac ab4729 Abcam<br>H3K4me2 (Y47) ab32356 Abcam<br>H3K4me3 07-473 Millipore
Validation	Rabbit polyclonal SMARCB1/SNF5 ChIP Grade antibody (ab12167) was used for immunoprecipitation purpose (RIP-ChIP), in line with manufacturer's guarantee (https://www.abcam.com/snf5smarcb1-antibody-chip-grade-ab12167.html). Mouse monoclonal SMARCB1/SNF5 antibody (ab58209) was used for western blot (now discontinued).

Eukaryotic cell lines

Policy information about cell lines

Cell line source(s)	ATCC
Authentication	None of the cell lines were authenticated.
Mycoplasma contamination	All cell lines were tested routinely for mycoplasma contamitation and resulted negative.
Commonly misidentified lines (See ICLAC register)	No misidentified cell lines were used in this study.

Animals and other organisms

Policy information about studies involving animals; ARRIVE guidelines recommended for reporting animal research

Laboratory animals	6-7 weeks old female BALB/cA-Rag2−/−γc−/− immunodeficient mice.
Wild animals	The study did not involve wild animals.
Field-collected samples	The study did not involve samples collected from the field.
Ethics oversight	The ethic protocol was submitted to the public spanish organization "Instituto de Salud Publica y Laboral de Navarra". The protocol was approved with the following code: 027-14, E55-16(027-14E2).

Note that full information on the approval of the study protocol must also be provided in the manuscript.

ChIP-seq

Data deposition

☒ Confirm that both raw and final processed data have been deposited in a public database such as GEO.

☒ Confirm that you have deposited or provided access to graph files (e.g. BED files) for the called peaks.

Data access links<br>*May remain private before publication.*	The following token (kdofmcaibnefhet) has been created to allow reviewer access to GEO SuperSeries GSE128327, which contains the GSE131711 subseries collecting ChIP-seq data.
Files in database submission	GSM3814488 H3K27ac_ChIP-seq<br>GSM3814489 H3K4me2_ChIP-seq<br>GSM3814490 H3K4me3_ChIP-seq<br>GSM3814491 SMARCB1_ChIP-seq<br>GSM3814492 SMARCB1_ASO_CTRL_repl1_ChIP-seq<br>GSM3814493 SMARCB1_ASO_CTRL_repl2_ChIP-seq<br>GSM3814494 SMARCB1_ASO_LINC_repl1_ChIP-seq<br>GSM3814495 SMARCB1_ASO_LINC_repl2_ChIP-seq<br>GSM3814496 H3K27ac_ASO_CTRL_repl1_ChIP-seq<br>GSM3814497 H3K27ac_ASO_CTRL_repl2_ChIP-seq<br>GSM3814498 H3K27ac_ASO_LINC_repl1_ChIP-seq<br>GSM3814499 H3K27ac_ASO_LINC_repl2_ChIP-seq
Genome browser session (e.g. UCSC)	*Provide a link to an anonymized genome browser session for "Initial submission" and "Revised version" documents only, to enable peer review. Write "no longer applicable" for "Final submission" documents.*

Methodology

Replicates	SMARCB1, SMARCB1_ASO_CTRL, SMARCB1_ASO_LINC, H3K27ac_ASO_CTRL, H3K27ac_ASO_LINC ChIP: 2 replicates
Sequencing depth	Multiplexed libraries were sequenced in a 75bp pair-end mode, with a depth of at least 20×106 reads/sample.
Antibodies	SMARCB1/SNF5 ab12167 Abcam

Antibodies	H3K27ac ab4729 Abcam<br>H3K4me2 (Y47) ab32356 Abcam<br>H3K4me3 07-473 Millipore
Peak calling parameters	For H3K27ac, H3K4me2, H3K4me3 and SMARCB1 ChIPs peak calling was performed with the following parameters: --bw 350 -q 0.01 without Input option<br>For H3K27ac_ASO_CTRL and H3K27ac_ASO_LINC ChIP, -q 0.001; for SMARCB1_ASO_CTRL, SMARCB1_ASO_LINC ChIP -q 0.0001.
Data quality	FastQC tool was used to assess sequencing quality.
Software	ChIP-seq fastq files were aligned to the human reference genome (hg19), BAM files were sorted and PCR replicates were removed using bowtie2, PICARD and samtools. BedGraph and BigWig files were generated using bedtools and bedGraphToBigWig tools.<br>ChIP-seq peaks were determined by performing MACS2 (version 2.1.0) peak calling.

Flow Cytometry

Plots

Confirm that:

- [ ] The axis labels state the marker and fluorochrome used (e.g. CD4-FITC).
- [ ] The axis scales are clearly visible. Include numbers along axes only for bottom left plot of group (a 'group' is an analysis of identical markers).
- [ ] All plots are contour plots with outliers or pseudocolor plots.
- [x] A numerical value for number of cells or percentage (with statistics) is provided.

Methodology

Sample preparation	For apoptosis assessment, IMR90 fibroblasts and H226 lung cancer cells were grown, treated and quickly detached using Accutase solution (Lonza). Apoptosis was assayed by Annexin V and 7-AAD staining using the Apoptosis Detection Kit I 791 (BD Biosciences).
Instrument	FACSCalibur flow cytometer
Software	Flow cytometry data were recorded by BD CellQuest program and analyzed using the FlowJo software.
Cell population abundance	Apoptotic cells represented 5-10% of total cell population.
Gating strategy	Gating parameters were set using different controls samples as untreated cells and cells incubated only with 7-AAD or Annexin V antibodies.

- [ ] Tick this box to confirm that a figure exemplifying the gating strategy is provided in the Supplementary Information.
