# Supplementary material for: A lncRNA-SWI/SNF complex crosstalk controls transcriptional activation at specific promoter regions
Source: Nat Commun. 2020 Feb 18;11:936. doi: 10.1038/s41467-020-14623-3 (PMC7028943; doi:10.1038/s41467-020-14623-3)
Supplement: Supplementary file 4 — Description of Additional Supplementary Files [file 41467_2020_14623_MOESM4_ESM.docx]

**Description of Additional Supplementary Files**

File name: Supplementary data 1

Description: List of genes enriched in SMARCB1 RIP-seq in proliferating conditions

File name: Supplementary data 2

Description: List of genes enriched in SMARCB1 RIP-seq in senescent conditions

File name: Supplementary data 3

Description: List of ncRNAs enriched in SMARCB1 RIP-seq in proliferating conditions presenting a SMARCB1 ChIPseq peak

File name: Supplementary data 4

Description: List of differentially expressed genes upon SWINGN knockdown in H226 lung squamous carcinoma cells

File name: Supplementary data 5

Description: List of genes differentially expressed upon SWINGN knockdown presenting a concordant change in SMARCB1 or H3K27ac binding

File name: Supplementary data 6

Description: List of antibodies, primers, siRNAs, sgRNAs, ChIRP and ddPCR probes sequences used in this study.
