# Supplementary material for: A lncRNA-SWI/SNF complex crosstalk controls transcriptional activation at specific promoter regions
Source: Nat Commun. 2020 Feb 18;11:936. doi: 10.1038/s41467-020-14623-3 (PMC7028943; doi:10.1038/s41467-020-14623-3)
Supplement: Supplementary file 2 — Peer Review [file 41467_2020_14623_MOESM2_ESM.pdf]

Reviewers' comments:

Reviewer #1 (Remarks to the Author):

review NCOMMS-19-15234
A lncRNA-SWI/SNF complex crosstalk controls transcriptional activation at specific promoter regions

Grossi E et al. use RIP to identify RNA interaction partners of the SWI/SNF subunit SMARCB1 in a model of cellular senescence. LINC00565 is among the RNAs identified to preferentially bind less to SMARCB1 in senescent conditions. LINC00565 is a not well described lncRNA which's biological functions are unknown. The authors explore a possible dual role of LINC00565 as both a regulatory RNA component of the SWI/SNF complex by binding to SMARCB1, and a local enhancer(-like) element for the neighboring GAS6 gene.
Knowledge about the effect of RNA on chromatin remodeling activity is limited, and the biological functions of LINC00565 have not been explored. Thus, the results here explore two interesting epigenetic regulatory circuits: (i) non-coding transcription units traditionally annotated as lncRNAs might in fact (also?) have a function as an enhancer and (ii) lncRNAs as components of chromatin remodeling.
However, the test case of GAS6 that the authors choose for both activities makes it hard to disentangle between the supposed in cis enhancer functions and in trans SWI/SNF remodeling. Adding to the complexity of the findings, SMARCB1 is described to bind at least one other lincRNAs, SChLAP1 https://www.nature.com/articles/ng.2771. Binding of SChLAP1 is described to antagonize SWI/SNF activity. Here, Grossi et al suggest a different mechanism by which LINC00565 binding of SMARCB1 would instead be a positive regulator of SWI/SNF dependent chromatin remodeling activity. The molecular basis for such opposing roles is not addressed on an experimental level and unfortunately also not discussed.
The manuscript describes an interesting phenomenon. However, it would highly benefit from a more detailed experimental analysis. The paper also suffers from technical errors in manuscript preparation that need to be addressed before publication is possible.

Major:
1) The manuscript lacks from proper descriptions of several experimental conditions in the results and methods section. E.g., the manuscript is inconsistent in the usage of ChIRP (text and methods) v. CHART (figure legends). While both methods are very similar, this needs to be cleared up. Additionally, some methods and material descriptions are missing (e.g. NORAD qPCR primers in Table S6). The controls for these assays need to be more comprehensive as false positives abound with this method.
2) The authors correlate the RNAs that interact with SMARCB1 (identified by RIP) to genomics regions bound by SMARC1B ChIP to make the case that SMARC1B binds these RNAs at the site of their transcription. Both from reading the manuscript text and the figures I was unable to reasonably identify what subset of RIP identified RNAs was used to make that point; and it is still unclear to me at this point. 2E seems to look at the total number of RNAs identified by RIP for proliferating condition is stated to be 1217 + 424 = 1641. In contrast, the number of ncRNA from 1C and mRNA from S1G under proliferating conditions adds up to 304 + 177 + 568 + 412 = 1461. This part of the analysis needs either clarification what all these different subsets are that make up the "total" in 2E, or much more explaining in the manuscript why the numbers are different.
3) The track for the two genome browser screenshots for SMARCB1 for the "-4OHT" of Fig 3A and the "proliferating" condition in 3B seems to depict the same experimental conditions, but the actual track is quite different (uniform SMARCB1 along the whole TCONS_00022133 in 3A, markedly enhanced binding peak at the LINC00565 part of TCONS_00022133 in 3B). Please explain.
4) The authors do a comprehensive analysis of LINC00565 biochemical properties. One missing piece of information is the estimated copy number per cell. Including this in the manuscript can make the discussion on the biological effects of the lncRNA more valuable.
5) It is unclear how SMARCB1 binds to LINC00565, especially as the protein does not have classical

RNA binding domains. While the use of CLIP seems to argue for a close proximity it does not exclude unspecific recruitment by other SWI/SNF components. If not addressed experimentally (e.g. by using recombinant expressed protein + lincRNA binding assays), this should at least receive a more extensive discussion. As every lncRNA can bind large number of proteins at low nM affinity, controls of specificity are quite necessary.
6) The HiC data shown in Fig. 3C seem to indicate that GAS6 and LINC00565 are present in the same TAD. However, the resolution is still not sufficient to draw conclusions for specific interactions. The source of the HiC data and how they were analyzed is not stated (is it ref. 34 as stated in the text or http://promoter.bx.psu.edu as stated in figure legend, and in the latter case what is the original source of the data?). Furthermore, a 4C type experiment could strengthen this aspect of the manuscript, if interaction between the GAS9 promoter and lncRNA/enhancer can be shown in one the cell models and conditions used here.
7) In the Volcano plot in Fig 1B, can the authors mark the ncRNA later chosen for individual analysis by qPCR (1E)?
8) The cross-referencing towards other figures in figure legends makes it unnecessarily confusing to follow the flow of information.

Reviewer #2 (Remarks to the Author):

The manuscript by Grossi et al. describes an interesting study of the function of a lncRNA (LINC00565) found in a screen for lncRNAs that bind the SWI/SNF complex. The authors use SMARCB1 subunit of the SWI/SNF complex to identify lncRNAs that bind the complex and select LINC00565 as a lncRNA that shows higher level of interaction with SMARCB1 in proliferative versus senescent cells. They show that the lncRNA originates from an enhancer region and can impact the SWI/SNF-mediated regulation of a number of genes with roles in cellular proliferation/survival. The authors use a variety of techniques including ChIP-seq, RNA-seq, ChIRP and RIP studies to tease apart the mode of function of LINC00565. While this is certainly a very interesting study, several important shortcomings must be addressed prior to publication, as detailed below.

Major points:

1) The authors need to clarify what method/kit was used to prepare the RNA-seq libraries used in this study. Without knowing the kit and the way the strand-specificity was preserved (e.g. dUTP method versus differential primer usage) it is impossible to judge the claims made in lines 181-182, since some level of “bleeding” is common with the differential primer usage methods.

2) Line 196-198: Fig. S3H only contains GRO-Seq data for GAS6, not for the lncRNAs, which should be included.
3) Unfortunately the knock down studies in Fig. 3E only show data for one siRNA. To ensure the observed results are not partly/completely resulting from off-target effects, a second siRNA/siRNA mix that does not have overlap with the first knock down construct(s) should be used in parallel. This is a critical control and must be performed.
4) Can the authors comment on why in Fig. S4B, using the ASO4 knock down construct leads to a reduction in the level of LINC00565 but not a change in the level of GAS6? The authors also should show the targeting site of each of the LINC00565-targeting ASOs on this lncRNA, for example in Fig. S3.
5) For the data in Fig. S5C and 4A, the statistics should be recalculated against the null hypothesis that randomly selected cellular RNAs/proteins may show such a correlation, since the correlations shown are rather weak. Especially for genes upregulated in cancer, the likelihood of observing such a correlation by chance is not negligible.
6) Line 265: The authors should describe their data in a precise manner. Here, at least for one of the three ASOs, there is a correlated reduction in GAS6 level. This needs to be indicated in the text to

allow the reader to accurately judge the level of correlation.
7) Line 272: it is not clear why the negative data for cell growth in HCT116 cells is not shown. Negative data are important and should be included in the supplementary results.
8) The ChIP-seq data showing the impact of knock down of LINC00565 on SMARCB1 binding and H3K27ac peaks is key to the scientific value of this study, however, the paragraph describing these results (starting at line 297) is extremely poorly written and the data as described are contradictory and thus, the entirety of these studies cannot be scientifically evaluated.
First, it is not indicated in the text that there is a global impact on H3K27ac peaks upon LINC00565 knock down (which is shown in Fig. S6E). This must be included.
Second, the second panel of Fig. S6F is missing, but is described in the legend to this figure. This data is not shown elsewhere but it’s very important to include and must be included.
Third, there is a claim that the positions that showed decreased binding to SMARCB1 also have reduced H3K27ac signal, but this reduction seems to be at the same level as the global reduction in H3K27ac signals. If the authors really believe that this is the case, then, they need to show whether the H3K27ac signal has been differentially altered between the 1) global H3K27ac peak loci, 2) at SMARCB1 sites that show altered binding to this protein, and 3) SMARCB1 sites that do not show binding changes to this protein as a result of the knock down.
Forth, either Fig. 5D is incorrectly described, or the figure is incorrect. Line 303: Since the total number of peaks that showed altered binding for SMARCB1 is 4185 according to line 300, the number shown for "positions that showed a decreased binding of SMARCB1 also suffered a concomitant and significant decrease of H3K27ac signal" cannot be more than 4000 loci, so the number shown in Fig. 5D is incorrect. The authors either need to redo the meta peak analysis in Fig. 5D for the doubly-reduced sites, or they should alter the text to indicate what the 9000+ loci correspond to.
Fifth: Line 300: it should be indicated what the total number of SMARCB2 and H3K27ac peaks has been in this study, in order for the reader to understand the magnitude of the change described in this line.
Sixth: The authors also need to include side by side metapeak graphs comparing the global versus the "altered subset" (e.g. the peaks described in line 300) peak level change as a result of lncRNA knockdown in order to allow the reader to judge the magnitude of the change described in this manuscript. Instead of including a single line, it may be a good idea to use a poly-line graph (one for each peak) to allow the variability in the magnitude of the change to become visible.
Seventh: Line 306: if the rationale behind these analyses is that reduced SMARCB1 may result in reduced acetylation, then this study must be limited to the subset that shows reduced SMARCB1 binding. Thus, both figure 5D and 5E must be redone.
Finally, and importantly, for all graphs shown related to these ChIP-seq studies, it should be clearly indicated exactly what subset is being studied in each graph.
9) Line 314: instead of cherry picking, the authors need to described the results of an unbiased comparison between the ChIP-seq and RNA-seq data: how many sites/genes did not show a change in one study but did change in the other? In what direction? How many showed changes in both sets of studies and in what directions? After this data is clearly described, the authors can proceed to pick their favorite subset for additional analysis.
10) The ChIRP studies in Fig. 5K are performed using lacZ as control. It is important to use a control which is expressed at a moderate to high level in the cells under study to determine the level of non-specific enrichment by the antisense pull down oligos. Similarly, for the Fig. 5L study, a locus that is expressed should be used to determine the level of enrichment of the captured chromatin loci.
11) Does knock down of PDGFRB at least partially recapitulate the impact of knock down of LINC00565? This is a rather simple study and should be performed, as the entire rationale for the studies detailed in the text after line 290 is the hypothesis that there must be other genes that mediate the impact of LINC00565 on proliferation. Even if knock down of PDGFRB regults in a small effect on proliferation, it will help validate the main point of this manuscript.

Minor points:

1) Line 119: This sentence should be re-written, since in its current form it implies that the differential

expression is playing the major role in defining the binding pattern.
2) Line 175: the authors should comment on the annotations of this locus using a more recent version of GENCODE, e.g. V29 or 30 instead of V19. lncRNA annotations have undergone dramatic changes since V19.
3) Line 270: The data in Fig 4F and S5H show a reduced tumor growth rate, rather than reduced capacity of forming tumors. This should be corrected in the text.
4) Line 278: It should be clarified in this sentence that the observed effect in BJ cells is reduced proliferation.
5) Are the GO terms listed in Fig. 4I positively or negatively enriched in response to the knock down of the lncRNA?
6) Line 288: the authors are referring to Fig. 4G, not 5G.
7) Line 302: The signals were reduced, not lost. This should be corrected in the text.

Reviewer #3 (Remarks to the Author):

Long noncoding RNAs (lncRNAs) have traditionally been linked with gene silencing. In this manuscript, Grossi et al., explore a novel association between lncRNAs and SMARCB1- a core member of the multimeric SWI/SNF chromatin remodeling complex to regulate transcriptional activation. To explore this association, the authors employ a dynamic model of oncogene induced senescence in BJ fibroblasts, wherein the SWI/SNF complex is thought to be partly responsible for chromatin re-organization associated with the induction of senescence. The authors initially perform RNA immunoprecipitation with SMARCB1 and identify a previously uncharacterized enhancer lncRNA, LINC00565, as one of several that preferentially interact with the SWI/SNF complex in proliferating compared to senescent BJ fibroblasts. The authors confirm that SMARCB1 is mainly bound at active promoter and enhancer sites and demonstrate that SMARCB1 is bound to LINC00565. Grossi et al., show that SMARCB1 bound LINC00565 can functionally regulate the oncogene GAS6 within it's co-located topologically associated domain (TAD), and further that this interaction is SMARCB1 dependent, at least in this cell context. Lastly, the authors demonstrate that LINC00565 promotes SMARCB1 occupancy at active proximal sites and can influence SMARCB1 regulated gene-expression programs.

Overall, the authors identify a novel enhancer lncRNA, LINC00565, and identify a functional link with the SWI/SNF complex in regulating transcriptional activation at promoters. In general, the framing of the manuscript and data presentation is fairly clear. There are weaknesses in the manuscript, principally that it is unclear whether the data robustly support the conclusions and stated models.

Major points
• The chromatin complex that is being studied, SWI/SNF, is a very large complex and a concern is the potential for false positive findings of RNA interactions/function. This has previously been an issue as Prenser et al reported interaction of SMARCB1, a paper that was refuted earlier this year (both in Nature Genetics). Consequently, rigor and validation are critical concepts. The methods by which the interaction RNAs are indentified and statistically validated are rather brief and should be expanded to enhance confidence that the interactions are specific.

• With respect to this, the authors claim that the absence SMARCB1 is required for the interactions of LINC00565 and GAS6 and show that the interactions are absent in SMARCB1 deficient AT/RT cells. Subsequently, the authors show that the interactions between LINC00565 and GAS6 are context-dependent. Consequently, it is not the clear extent to which the model is accurate. To rigorously test the role of SMARCB1 in mediating these interactions, the authors could perform a rescue experiment to re-express SMARCB1 in AT/RT cells and test whether the LINC00565 and GAS6 interactions are restored.

• In more detail, of the 1644 genes differentially regulated by LINC00565 knockdown, only 85 also showed LINC00565-dependent changes in SMARCB1 and chromatin status. Of those 85 genes, the researchers mention GAS6, PDGFRB, and COL1A1 as being part of "a LINC00565 and SWI/SNF-dependent oncogenic hub." (line 327). It is not clear how these three genes show that LINC00565 and SWI/SNF form an oncogenic hub. Showing data or discussing other work to support the importance of these genes +/- SMARCB1 and/or in other cited cancer contexts would have the potential to strengthen this conclusion.

• The work presented in Figure 2, the finding that SMARCB1 is present at both active enhancers and promoters is not novel. The cited papers show that SMARCB1 containing complexes BAF and PBAF have been shown to occupy active enhancers and promoters (Wang X et al., 2017, Nakyama et al., 2018).

Minor points

• There are several grammatical/spelling issues

• The sentence that starts on line 136 is six lines long and quite difficult to understand.

• In Figure 2C, it would be helpful to see the SMARCB1 ChIP-Seq heatmap along-with H3K4me2, H3K4me3 and H3K27ac.

• The authors should cite Weissmiller et al., as they also have recently published SMARCB1 ChIP-Seq datasets.

• The co-immunoprecipitation experiments performed in S1E are unconvincing and are of poor quality. They should either be removed from the figure panel or should be repeated for clearer results.

• The differences seen in the tumor volumes in Figure 4F upon knockdown of LINC00565 in immunodeficient mice are at best modest and don't add much to the manuscript.

We thank the reviewers for their insightful comments and we very much appreciate that they found the original paper interesting and potentially significant. We have been stimulated by the points raised to perform substantial further experimental work, and trust that the reviewers will now agree that this has improved the paper sufficiently to render it acceptable for publication in Nature Communications.

Following the reviewers' suggestions, we now include new data and analyses to further support the following observations;

1. The interaction between SWI/SNF and some lncRNAs, including *LINC00565*, is specific and likely mediated by SMARCB1 complex subunit.
2. A functional co-dependency exists between *LINC00565* and SWI/SNF, resulting in the activation of specific gene promoters in an RNA-dependent manner.
3. The loss of *LINC00565* impairs the binding of SWI/SNF to multiple gene loci linked to a reduction in H3K27ac.
4. Genes activated in a coordinated manner by *LINC00565* constitute a pro-oncogenic gene network that promotes the proliferation of the cancer cells.

In order to incorporate all this new supporting evidence we have included a new main figure, several new figure panels, three new supplementary figures, have expanded some sections of the results as well as the discussion.

Please note that **we have renamed *LINC00565* as *SWINGN* (*SWI/SNF Interacting GAS6 enhancer Noncoding RNA*).** However, for simplicity, in the responses to reviewers we will still refer to it as *LINC00565*.

Our responses to the individual points raised by the reviewers are presented below as direct response to each reviewer's questions.

Reviewer comments:

Reviewer #1: Summary

Knowledge about the effect of RNA on chromatin remodeling activity is limited, and the biological functions of LINC00565 have not been explored. Thus, the results here explore two interesting epigenetic regulatory circuits: (i) non-coding transcription units traditionally annotated as lncRNAs might in fact (also?) have a function as an enhancer and (ii) lncRNAs as components of chromatin remodeling.
However, the test case of GAS6 that the authors choose for both activities makes it hard to disentangle between the supposed in cis enhancer functions and in trans SWI/SNF remodeling. Adding to the complexity of the findings, SMARCB1 is described to bind at least one other lincRNAs, SChLAP1 https://www.nature.com/articles/ng.2771. Binding of SChLAP1 is described to antagonize SWI/SNF activity. Here, Grossi et al suggest a different mechanism by which LINC00565 binding of SMARCB1 would instead be a positive regulator of SWI/SNF dependent chromatin remodeling activity. The molecular basis for such opposing roles is not addressed on an experimental level and unfortunately also not discussed.
The manuscript describes an interesting phenomenon. However, it would highly benefit from a more detailed experimental analysis. The paper also suffers from technical errors in manuscript preparation that need to be addressed before publication is possible.

We thank the reviewer for highlighting the interest of our study. We agree with him/her that the effect of SMARCB1 interaction with RNA is a relevant and complex point that deserves more detailed examination. We now explore this subject with additional experimental data as well as a more comprehensive discussion of the issues and hypotheses detailed in the responses below.

We agree that it is not easy to disentangle the cis/enhancer and trans functions of *LINC00565,* which we believe is a common scenario for many lncRNAs. In order to address this issue we applied orthogonal complementary methodologies that are able to point out RNA-dependent effects and their consequences in *cis* and *trans* (ASO-mediated knockdowns, CRISPRa-mediated

LINC00565 perturbation combined with gene expression and chromatin analyses as well as detection of RNA direct binding to chromatin loci by ChIRP). Taking together the abundant data provided we can confidently conclude that *LINC00565* gene locus has the characteristics of a transcriptional enhancer, but RNA transcript produced from it has both *cis* and *trans* roles that involve SWI/SNF-dependent gene activation.

As the reviewer correctly points out, the prostate cancer-specific *SChLAP1* was initially reported to interact with SMARCB1 and antagonize SWI/SNF activity by preventing complex binding [1]. However, this finding was recently questioned by following analyses showing that *SChLAP1* modulation does not alter SMARCB1 chromatin occupancy [2].
Nevertheless, beyond the case of *SChLAP1*, emerging evidence suggest that the interactions between RNA and SWI/SNF can have opposite roles. While a recent publication showed that the lncRNA *Xist* antagonizes the ATPase activity of the SWI/SNF core subunit SMARCA4 (BRG1) [3], *7SK* small nuclear RNA has been shown to direct the SWI/SNF complexes to enhancer elements and control pervasive transcription at these regulatory loci [4]. We speculate that the specific chromatin and transcriptional context in which the interactions take place is key to the different outcomes of the interaction between ncRNAs and the SWI/SNF complexes, however these cannot be easily uncoupled. Moreover, multiple elements such as specific RNA structures or different SWI/SNF associated factors, which are known to modulate and define different facets of SWI/SNF complex activity, might influence. In this context, we can hypothesize that an RNA associating with a SW/SNF enzymatic subunit (such as SMARCA4) might have different effects on complex functionality compared to an interaction with a non-catalytic subunit, as SMARCB1, the subunit likely bound by *LINC00565.*

In summary, although it is difficult to find a unifying mechanism of the functional crosstalk between RNA and SWI/SNF complexes, our study provides additional evidence of their co- dependency by demonstrating that *LINC00565* mediates SWI/SNF binding and transcriptional activation at specific gene loci.

Major:
1) The manuscript lacks from proper descriptions of several experimental conditions in the results and methods section. E.g., the manuscript is inconsistent in the usage of ChIRP (text and methods) v. CHART (figure legends). While both methods are very similar, this needs to be cleared up.

We thank the reviewer for pointing out to these shortcomings. The reason for the inconsistency in the ChIRP/CHART description is due to the fact that we have used a modified protocol that implements elements from both ChIRP and CHART, which, as pointed out by the reviewer, are very similar. To keep consistency we have now corrected the figure relative to ChIRP experiment in the legend of Figure 5J-K, and have expanded the methods section relative to this assay. In addition, per reviewer's #2 suggestion, we have added further controls to ensure that *LINC00565* binding to the genomic regions of interest was specific to this lncRNA (**see response to Reviewer #2 below**).

Additionally, some methods and material descriptions are missing (e.g. NORAD qPCR primers in Table S6). The controls for these assays need to be more comprehensive as false positives abound with this method.

We revised material and methods section to improve the description of several experimental conditions. Now we also include information corresponding to the new data added to the revised version of the manuscript. NORAD qPCR primers along with primers used in 3C and ChIRP experiments are now shown in Supplementary Table 6.

2) The authors correlate the RNAs that interact with SMARCB1 (identified by RIP) to genomics regions bound by SMARC1B ChIP to make the case that SMARC1B binds these RNAs at the site of their transcription. Both from reading the manuscript text and the figures I was unable to reasonably identify what subset of RIP identified RNAs was used to make that point; and it is still unclear to me at this point. 2E seems to look at the total number of RNAs identified by RIP for

proliferating condition is stated to be 1217 + 424 = 1641. In contrast, the number of ncRNA from 1C and mRNA from S1G under proliferating conditions adds up to 304 + 177 + 568 + 412 = 1461. This part of the analysis needs either clarification what all these different subsets are that make up the "total" in 2E, or much more explaining in the manuscript why the numbers are different.

We apologize for not stating clearly enough the subset of SMARCB1-interacting RNAs that we took into account to compare with ChIP-seq data. The total number of RNAs identified by RIP in proliferating cells is indeed 1641. This number includes 481 ncRNAs (including lincRNA, antisense, miscRNA, scaRNA and miRNA categories), 980 mRNAs and 180 RNAs transcribed from pseudogenes that we excluded from further analyses. This filtering is now clearly described in materials and methods section as well as mentioned in Figure legend 2E.

3) The track for the two genome browser screenshots for SMARCB1 for the "-4OHT" of Fig 3A and the "proliferating" condition in 3B seems to depict the same experimental conditions, but the actual track is quite different (uniform SMARCB1 along the whole TCONS_00022133 in 3A, markedly enhanced binding peak at the LINC00565 part of TCONS_00022133 in 3B). Please explain.

While the genome browser track presented in Fig 3A corresponds to SMARCB1 *RIP-seq* analysis in proliferating cells, the screenshot in Fig 3B represents SMARCB1 *ChIP-seq* track in the same -4OHT proliferating condition. We understand that the way we presented the two panels might be misleading so we modified Figure 3A-B by clearly stating the nature of the experiment.

4) The authors do a comprehensive analysis of LINC00565 biochemical properties. One missing piece of information is the estimated copy number per cell. Including this in the manuscript can make the discussion on the biological effects of the lncRNA more valuable.

This data is included now in Supplementary Fig. 3F. To precisely measure *LINC00565* copy number per cell, we took advantage of droplet digital PCR (ddPCR) and Taqman probes designed to detect *LINC00565* long and short isoforms. We estimated *LINC00565* to be present with around 8 copies per cell in BJ fibroblasts.
We found that the depletion of *LINC00565* alters SMARCB1 binding at multiple loci with a consequent gene expression change. We envision that only a limited number of these loci are direct targets of *LINC00565*. These sites probably are in physical proximity within the nucleus, allowing their coordinated regulation by a relatively small number of RNA molecules. This has been suggested for other lncRNAs and chromatin complexes that can regulate multiple distant loci by contacting them simultaneously in the nucleus [5,6]. The perturbation in SWI/SNF binding at these primary genomic sites might lead to an indirect secondary effect on chromatin occupancy at additional loci. Alternatively, it cannot be excluded that there is certain level of stochasticity and some of the *LINC00565* targets may vary in different cell subpopulations, detecting our analysis the sum of them.
Consistently with our observations and proposed model, novel techniques such as HiChIRP, have been recently developed to detect the binding and effect in chromatin organization of lowly expressed lncRNAs [7]. For instance, it has been demonstrated that lincRNA-EPS (~10 copies/cell) is able to contact hundreds of sites in the genome thanks to its interaction with the SWI/SNF complexes and its depletion perturbs chromatin looping at hundreds of loci [7,8].
These novel studies point out that even lowly expressed lncRNAs can function on multiple distant genomic loci by taking advantage of particular 3D chromatin structure and epigenetic complexes that amplify lncRNA regulatory effects.

5) It is unclear how SMARCB1 binds to LINC00565, especially as the protein does not have classical RNA binding domains. While the use of CLIP seems to argue for a close proximity it does not exclude unspecific recruitment by other SWI/SNF components. If not addressed experimentally (e.g. by using recombinant expressed protein + lincRNA binding assays), this should at least receive a more extensive discussion. As every lncRNA can bind large number of proteins at low nM affinity, controls of specificity are quite necessary.

Given the multi-subunit composition of SWI/SNF, we cannot precisely point out which subunit (or subunits) mediates the interaction with LINC00565 *in vivo*. However, we present additional evidence that suggest that SMARCB1 could be binding directly to *LINC00565*:

(i) Analyses of the protein sequence and structure predict RNA-binding activity for SMARCB1: FFPred3 (feature-based function prediction of all Gene Ontology domains [9]) predicts *RNA binding function* for SMARCB1 (probability=0.566, SVM reliability=HIGH). Moreover, SMARCB1 protein has a low complexity region (LCR), which, in the 3D protein model, together with coils from other domains, conforms two surfaces where the RNA-binding might occur (Fig. R1). Of note, MEME tool identified in this LCR a protein motif that, when analyzed by FIMO (Find Individual Motif Occurrences) algorithm, resulted enriched in a set of proteins with RNA binding as the most enriched GO Molecular Function term (GO:0003723, p-val=1.19E-04) (Fig R1).
(ii) Consistently with this prediction, the purified SMARCB1 protein is able to interact with RNA *in vitro* but presents a more efficient association with *LINC00565* than with other RNAs such as *HPRT* or *PR-lncRNA-1* (Figure R2, added as Supplementary Fig. 3A-B).
(iii) In addition, while the interaction between SMARCB1 and *LINC00565* in cells is detected under UV crosslinking conditions, the lncRNA does not bind the SUZ12 component of PRC2 chromatin repressive complex under the same experimental conditions (Figure R3, added as Supplementary Figs. 1I-J).

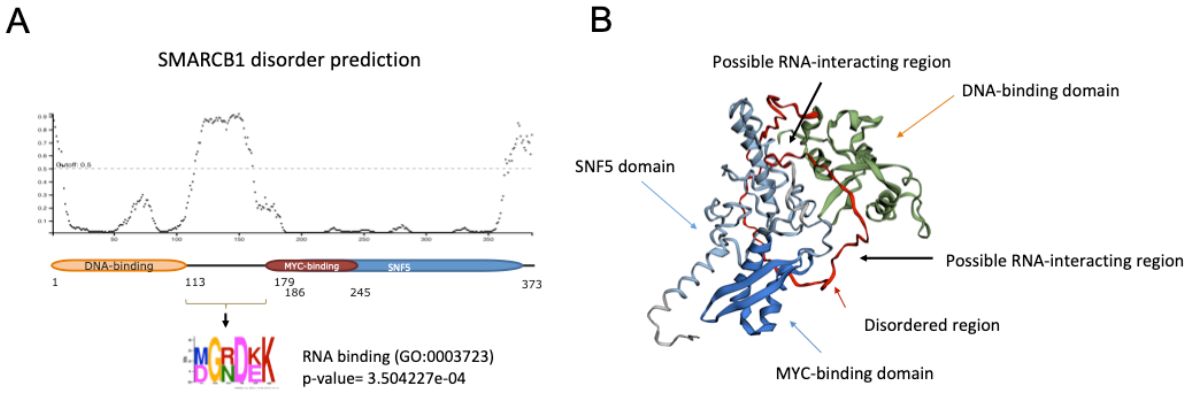

Figure R1. SMARCB1 is a potential RNA-binding protein. A) Disorder prediction of the secondary structure of SMARCB1. MEME motif identification in the LCR (amino acids 113-179) with RNA binding as the most enriched GO term. **B)** SMARCB1 3D structure model indicating the potential RNA-interacting region and the previously identified domains. The predicted disordered region is represented in red.

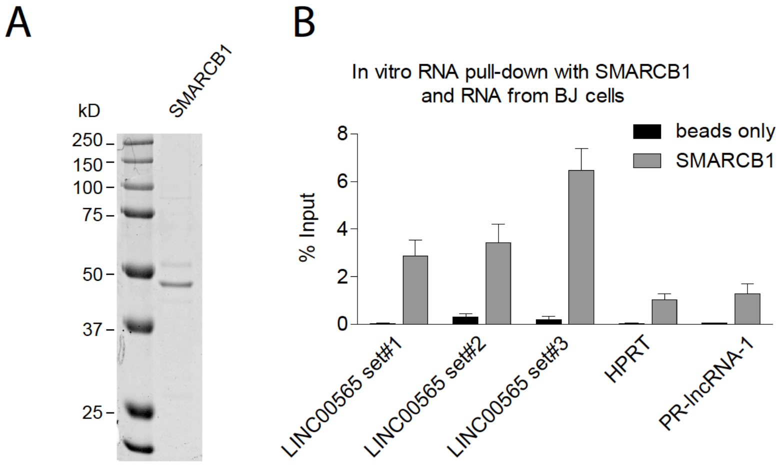

Figure R2. Interaction between purified SMARCB1 and LINC000565. (A) Coomassie stained-gel of SMARCB1 purified protein. **(B)** Enrichment of different RNAs from BJ fibroblasts determined by RT-qPCR in SMARCB1 pulldown or empty beads as control.

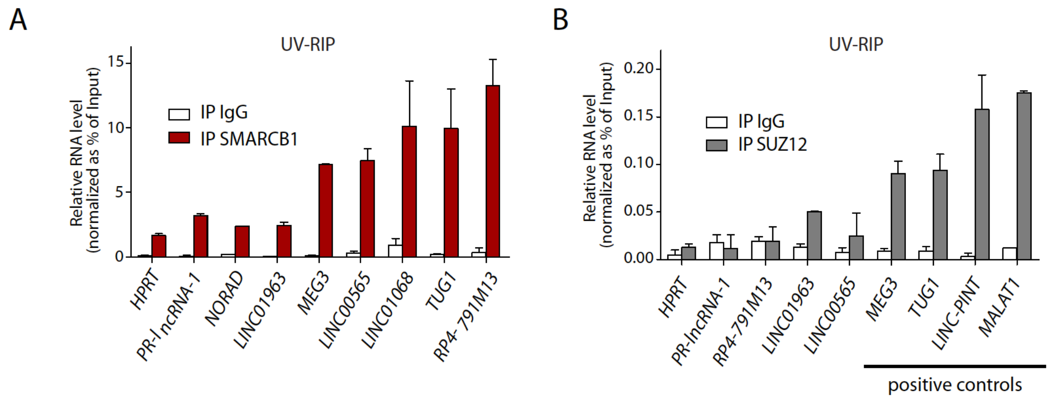

Figure R3. Specificity of the interaction between lncRNAs and SWI/SNF and PRC2 complexes evaluated by CLIP. RNAs in immunoprecipitates after UV crosslinking of SMARCB1 **(A)** and SUZ12 **(B)** proteins in proliferating BJ fibroblasts. *NORAD*, *LINC01963*, *MEG3*, *LINC00565*, *LINC01068*, *TUG1* and *RP4-791M13* had been detected as enriched in SMARCB1 RIP-seq. *MEG3*, *TUG1*, *LINC-PINT* and *MALAT1* were found interacting with PRC2 in previously published studies.

Together, these data support that SMARCB1 has the capacity to establish a direct interaction with *LINC00565* with some level of specificity. The biological specificity of the *in vivo* interaction is likely the combination of their intrinsic biochemical affinity and the physical proximity of the RNA and SWI/SNF in the nucleus.
Nevertheless, we agree with the reviewer that we cannot exclude that *in vivo* other SWI/SNF components or accessory factors might be participating in the interaction between *LINC00565* and SMARCB1, which is now added to the discussion.

For additional details related to this point please see the response to Reviewer #3.

6) The HiC data shown in Fig. 3C seem to indicate that GAS6 and LINC00565 are present in the same TAD. However, the resolution is still not sufficient to draw conclusions for specific interactions. The source of the HiC data and how they were analyzed is not stated (is it ref. 34 as stated in the text or http://promoter.bx.psu.edu as stated in figure legend, and in the latter case what is the original source of the data?). Furthermore, a 4C type experiment could strengthen this aspect of the manuscript, if interaction between the GAS9 promoter and lncRNA/enhancer can be shown in one the cell models and conditions used here.

To address this point, we performed a 3C analysis in BJ proliferating fibroblasts to study chromatin interactions between *LINC00565* locus (anchor) and *GAS6* promoter (bait) by using primer sets spanning this genomic region (Figure R4, added to manuscript as Supplementary Figs. 4D-E). These analyses confirmed with higher resolution the results observed in HiC public data, identifying an interaction between *LINC00565* and *GAS6* promoter.

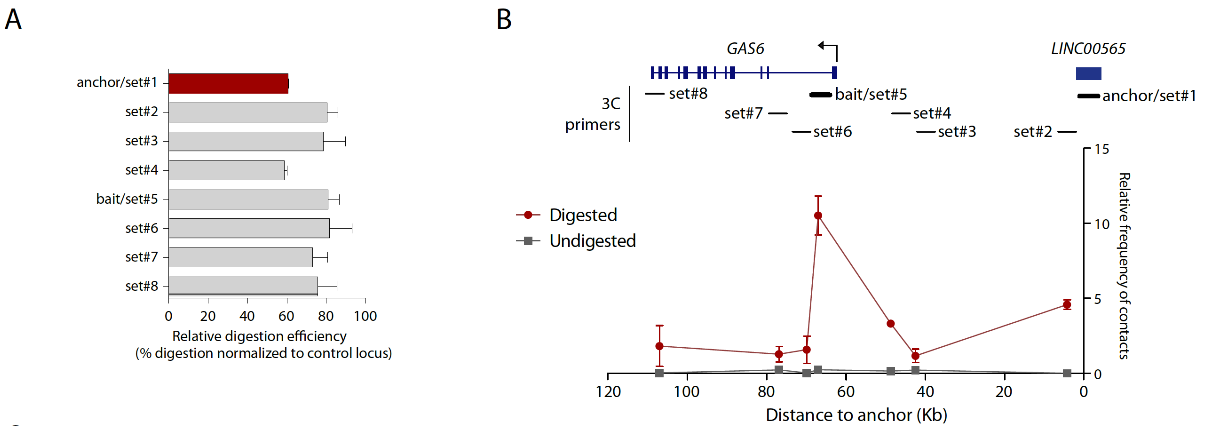

Figure R4. Detection of the interaction between GAS6 promoter and LINC00565 by 3C
A) Evaluation of digestion efficiency in BJ fibroblasts for the different primer sets spanning *LINC00565/GAS6* locus used for 3C analysis. **B)** (Upper panel) Schematic representation of primer set position. (Bottom panel) 3C assay followed by qPCR in BJ proliferating cells. Signal from HindIII-digested DNA was compared to undigested control. Data represent the average of three independent replicates.

We apologize for not stating clearly the source of the HiC these data. HiC image was generated by using the 3D genome browser [10], available at http://promoter.bx.psu.edu/hi-c. The original HiC experiment has been performed in IMR90 fibroblasts as described in [11].

7) In the Volcano plot in Fig 1B, can the authors mark the ncRNA later chosen for individual analysis by qPCR (1E)?

We now show some of the selected ncRNAs in Figure 1B.

8) The cross-referencing towards other figures in figure legends makes it unnecessarily confusing to follow the flow of information.

We removed cross-referencing.

Reviewer #2 (Remarks to the Author):

The manuscript by Grossi et al. describes an interesting study of the function of a lncRNA (LINC00565) found in a screen for lncRNAs that bind the SWI/SNF complex. The authors use SMARCB1 subunit of the SWI/SNF complex to identify lncRNAs that bind the complex and select LINC00565 as a lncRNA that shows higher level of interaction with SMARCB1 in proliferative versus senescent cells. They show that the lncRNA originates from an enhancer region and can impact the SWI/SNF-mediated regulation of a number of genes with roles in cellular proliferation/survival. The authors use a variety of techniques including ChIP-seq, RNA-seq, ChIRP and RIP studies to tease apart the mode of function of LINC00565. While this is certainly a very interesting study, several important shortcomings must be addressed prior to publication, as detailed below.
We thank the reviewer for showing his/her interest in our study.

Major points:

1) The authors need to clarify what method/kit was used to prepare the RNA-seq libraries used in this study. Without knowing the kit and the way the strand-specificity was preserved (e.g. dUTP method versus differential primer usage) it is impossible to judge the claims made in lines 181-182, since some level of "bleeding" is common with the differential primer usage methods.

RIP-seq libraries were prepared using the TruSeq Stranded Total RNA library prep Illumina kit. This protocol preserves the original RNA strand information by introducing a dUTP incorporation step in place of dTT during Second Strand Synthesis.
We now include this information in materials and methods section.
Nevertheless, we would like to note that we have further validated the sense of *LINC00565* transcript by strand-specific RT-qPCR (Supplementary Fig. 3E).

2) Line 196-198: Fig. S3H only contains GRO-Seq data for GAS6, not for the lncRNAs, which should be included.

We now include GRO-seq data of TIG3 lung fibroblasts at *LINC00565* locus as Supplementary Fig. 4B.

3) Unfortunately the knock down studies in Fig. 3E only show data for one siRNA. To ensure the

observed results are not partly/completely resulting from off-target effects, a second siRNA/siRNA mix that does not have overlap with the first knock down construct(s) should be used in parallel. This is a critical control and must be performed.

We understand reviewer's concern for off target effects due to the use of a single siRNA. In the first version of the paper we used a previously validated and published siRNA sequence [1]. Now, per reviewer's suggestion, we repeated SMARCB1 depletion using the original siRNA (siRNA#1) along with a new one (siRNA#2) and confirmed the effect on GAS6 RNA and protein levels (Figure R5, added as Figure 3E and Supplementary Fig. 4F). In addition, and as further control of the proposed model, we performed a siRNA-mediated knockdown of SMARCC1, another core component of the SWI/SNF complexes. We observed a consistent decrease in GAS6 expression levels (Supplementary Fig. 4G), reinforcing the notion that the SWI/SNF complexes control GAS6 expression.

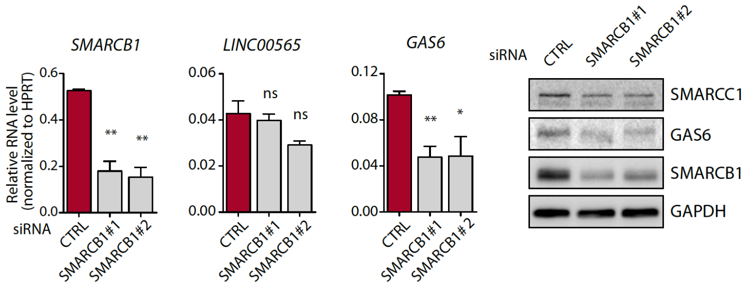

Figure R5. SMARCB1 knockdown causes GAS6 downregulation. RT-qPCR and western blot analyses of BJ proliferating cells depleted or not of SMARCB1 by siRNA double transfection. Graph shows mean ± SD of three independent experiments. Western blot picture refers to a representative experiment.

4) Can the authors comment on why in Fig. S4B, using the ASO4 knock down construct leads to a reduction in the level of LINC00565 but not a change in the level of GAS6? The authors also should show the targeting site of each of the LINC00565-targeting ASOs on this lncRNA, for example in Fig. S3.

Though very efficient in targeting nuclear-enriched RNAs, antisense oligonucleotides may have a certain level of unspecific targeting. To control for this possible issue, we used three different ASOs targeting *LINC00565* (designed and pre-tested by *Ionis Pharm. Inc*.), which consistently reduced lncRNA levels and decreased *GAS6* expression in different cell lines, such as BJ fibroblasts and H226 lung squamous cells. Only in IMR90 fibroblasts ASO#4 failed to downregulate GAS6. We believe this is a cell type-specific issue, perhaps due to the presence in these cells of an off-target sequence that compensates for *GAS6* downregulation, although a precise answer is difficult to provide. To demonstrate that this incongruence was related to cell variability, we repeated ASO-mediated depletion of *LINC00565* in an additional, unrelated cell line (A498 renal carcinoma), where the lncRNA was expressed at similar levels as in IMR90 fibroblasts. In these cells, *LINC00565* efficient knockdown reduced GAS6 levels with the 3 independent ASOs (including ASO#4) (Figure R6). ASOs targeting sites are now shown in Supplementary Fig. 3C.

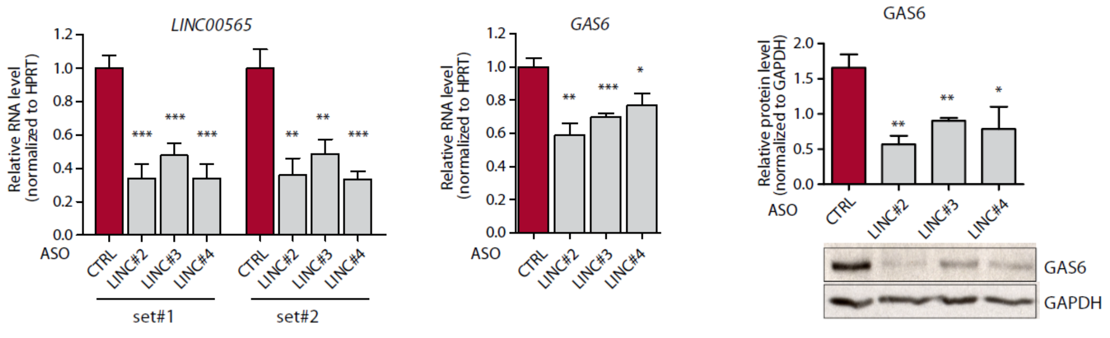

Figure R6. *LINC00565* depletion in A498 renal clear cell carcinoma. RT-qPCR analysis on the left panel, western blot analysis on the right.

5) For the data in Fig. S5C and 4A, the statistics should be recalculated against the null hypothesis that randomly selected cellular RNAs/proteins may show such a correlation, since the correlations shown are rather weak. Especially for genes upregulated in cancer, the likelihood of observing such a correlation by chance is not negligible.

The statistics of the data are presented in Figure 4A and Supplementary Figs. 6C and 9A (represented as p-values) and take into account the null hypothesis that random mRNAs might present the same level of correlation. In all the cases these p-values are extremely low (ranging from $4.5e^{-32}$ to $7.4e^{-76}$), supporting the highly significant statistical confidence of the analysis. Moreover, we respectfully disagree that the correlation coefficients (R) ranging from 0.49 to 0.62 are low, even more so when taking into consideration the high heterogeneity of cancer samples.

In addition, an unbiased search performed in The Atlas of ncRNAs in Cancer (TANRIC) (https://ibl.mdanderson.org/tanric/_design/basic/main.html) [12]. Found that most of the genes pointed out in our study, *GAS6*, *PDGFRB* and *COL1A1* included, were listed among the 50 genes most correlated with *LINC00565* expression in TCGA lung squamous carcinoma (LUSC) cohort.

6) Line 265: The authors should describe their data in a precise manner. Here, at least for one of the three ASOs, there is a correlated reduction in GAS6 level. This needs to be indicated in the text to allow the reader to accurately judge the level of correlation.

The observation of the reviewer is correct. In order to be more rigorous and also answer the following point raised by the reviewer (7) we included additional experimental replicates investigating *GAS6* gene expression upon *LINC00565* depletion in the colorectal cancer HCT116 cell line. We confirmed that in these cells the variation in *GAS6* mRNA expression is not significant. We reformulated the sentence in the text accordingly.

7) Line 272: it is not clear why the negative data for cell growth in HCT116 cells is not shown. Negative data are important and should be included in the supplementary results.

The negative data of cell growth in HCT116 cells upon *LINC00565* depletion is now included as supplementary data (Supplementary Figs. 6I-J). In these tumor cells lncRNA knockdown does not significantly affect proliferation rate or colony formation capacity.

8) The ChIP-seq data showing the impact of knock down of LINC00565 on SMARCB1 binding and H3K27ac peaks is key to the scientific value of this study, however, the paragraph describing these results (starting at line 297) is extremely poorly written and the data as described are contradictory and thus, the entirety of these studies cannot be scientifically evaluated.

We apologize for the lack of clarity. We have now improved both data presentation and description by modifying widely the text and reorganizing most of the figures, changing their order and providing them with technical details

First, it is not indicated in the text that there is a global impact on H3K27ac peaks upon LINC00565 knock down (which is shown in Fig. S6E). This must be included.

We now clearly indicate the global impact of *LINC00565* knockdown on H3K27ac.
The global analysis of H3K27ac occupancy only showed a slight effect upon *LINC00565* knockdown (p-value=0.01) (Supplementary Fig. 8B). This effect is not comparable to the reported strong global H3K27ac perturbation caused by SWI/SNF depletion [13,14]. In contrast, a stronger effect was observed when focusing just on the differential H3K27ac peaks (12097 peaks, p-value=$1.64e^{-07}$), resulting from a drop of H3K27ac in 99% of these regions (Figure 5D).

Second, the second panel of Figure S6F is missing, but is described in the legend to this figure. This data is not shown elsewhere but it's very important to include and must be included.

The data showing H3K27ac at SMARCB1 sites are included now in the new Figure 5E (see the expanded explanation below).

Third, there is a claim that the positions that showed decreased binding to SMARCB1 also have reduced H3K27ac signal, but this reduction seems to be at the same level as the global reduction in H3K27ac signals. If the authors really believe that this is the case, then, they need to show whether the H3K27ac signal has been differentially altered between the 1) global H3K27ac peak loci, 2) at SMARCB1 sites that show altered binding to this protein, and 3) SMARCB1 sites that do not show binding changes to this protein as a result of the knock down.

We understand reviewer's questions and concerns and we included further analyses to address them.

First, we showed the global H3K27ac regions separately for the two different conditions (ASO_CTRL and ASO_LINC, 49064 and 49912 sites respectively) in Supplementary Figure 8B. Then, we focused on differential H3K27ac peaks comparing ASO_CTRL and ASO_LINC ChIP data (12097 peaks), observing a stronger and more significant downregulation (Fig 5D).

We then set to investigate whether the significant decrease in acetylation was related to the reduction in SMARCB1 occupancy. For this, we analyzed H2K27ac signal at SMARCB1 sites previously identified in Figure 5C as differentially bound by this protein comparing ASO_CTRL and ASO_LINC conditions (Figure 5E, left panel). We observed a highly significant decrease in H3K27ac occupancy (p-value=1.7e-10; ED=45.83). In contrast, when we analyzed H3K27ac signal at all SMARCB1 sites (47272 regions shown in Supplementary Figure 7A), we found that the difference comparing CTRL and LINC conditions was strongly reduced (p=0.0003; ED=13.1). Importantly, when we subtracted the SMARCB1 differential peaks to the whole set of SMARCB1 regions (47272-4791) we observed an even stronger drop in the differences when comparing the two conditions (p=0.003; ED=9.98; Figure 5E, right panel). For more clarity, we have summarized these data in Table R1.

ANALYSIS of **H3K27ac** ChIP-seq **ASO_CTRL vs ASO_LINC**

Peak selection	number of peaks	p-value	ED
All H3K27ac	49064	0.01	10.21
Differential H3K27ac	12097	1.64e-07	39.82
All SMARCB1	47272	0.0003	13.1
Differential SMARCB1	4791	1.7e-10	45.83
Unchanged SMARCB1	42481	0.003	9.9

Table R1. Statistics associated to the H3K27ac ChIP-seq comparing control (ASO_CTRL) vs *LINC00565* knockdown (ASO_LINC) in the indicated subsets of peaks.

Taking into consideration these data we can conclude that the changes in H3K27ac are mainly associated to the decreased binding of SMARCB1 at specific loci. While we also detect some level of decrease in acetylation at additional loci, this is probably due to secondary effects of *LINC00565* knockdown. In summary, our data support that the main effect in histone acetylation upon *LINC00565* knockdown is linked to the reduction of SMARCB1 binding.

Forth, either Fig. 5D is incorrectly described, or the figure is incorrect. Line 303: Since the total number of peaks that showed altered binding for SMARCB1 is 4185 according to line 300, the number shown for "positions that showed a decreased binding of SMARCB1 also suffered a concomitant and significant decrease of H3K27ac signal" cannot be more than 4000 loci, so the number shown in Fig. 5D is incorrect. The authors either need to redo the meta peak analysis in

Fig. 5D for the doubly-reduced sites, or they should alter the text to indicate what the 9000+ loci correspond to.

We apologize for not stating clearly the subset of regions we used for this analysis. In the original version of Fig 5D, we analyzed H3K27ac occupancy at regions where H3K27ac mark was differentially present (12097 sites) that were also presenting a binding site for SMARCB1, even though not necessarily differentially bound (47272 regions). The intersection between these two datasets retrieved a subset of ~9000 loci. Nevertheless, we realized that this selection might be misleading and less informative of the relationship between change in SMARCB1 binding and acetylation occupancy. For this reason, we removed the graph and we substituted it with a new panel (Fig 5E), following reviewer's suggestions in his/her previous point.

Fifth: Line 300: it should be indicated what the total number of SMARCB2 and H3K27ac peaks has been in this study, in order for the reader to understand the magnitude of the change described in this line.

We now included the number of peaks found in this study, not only in this case but also for the remaining ChIP analyses related to Fig. 5 and Supplementary Fig. 8.

Sixth: The authors also need to include side by side metapeak graphs comparing the global versus the "altered subset" (e.g. the peaks described in line 300) peak level change as a result of lncRNA knockdown in order to allow the reader to judge the magnitude of the change described in this manuscript. Instead of including a single line, it may be a good idea to use a poly-line graph (one for each peak) to allow the variability in the magnitude of the change to become visible.

As suggested, we have included now the side-by-side metapeak graphs comparing both subsets peaks and the poly-line graphs in Supplementary Figures 8A-D.

Seventh: Line 306: if the rationale behind these analyses is that reduced SMARCB1 may result in reduced acetylation, then this study must be limited to the subset that shows reduced SMARCB1 binding. Thus, both figure 5D and 5E must be redone.
Finally, and importantly, for all graphs shown related to these ChIP-seq studies, it should be clearly indicated exactly what subset is being studied in each graph.

We appreciate reviewer's suggestion, and we now include a new Figure 5E analyzing acetylation exclusively at SMARCB1 differential peaks. Similarly, the chromatin state (former figure 5E) as well as the enrichment at proximal or distal loci analyses have been performed with SMARCB1 differential peak subset (Figure 5F and Supplementary Fig. 8F). In addition we kept original Figure 5C-D to show H3K27ac ChIP-seq data in an unbiased manner. We also ensured that all the necessary information in indicated in each graph.

9) Line 314: instead of cherry picking, the authors need to described the results of an unbiased comparison between the ChIP-seq and RNA-seq data: how many sites/genes did not show a change in one study but did change in the other? In what direction? How many showed changes in both sets of studies and in what directions? After this data is clearly described, the authors can proceed to pick their favorite subset for additional analysis.

We have now expanded this paragraph with more detailed description of the data.
Since we had described the role of *LINC00565* in the activation of *GAS6,* we focused on genes whose expression and chromatin status were similarly and concordantly altered. Interestingly, the gene expression analysis performed upon *LINC00565* knockdown showed that the downregulated set (|logFC|>1; B>0) was enriched in the same functional terms previously related to an epigenetic signature in AT/RT tumors dependent on SMARCB1 loss [15] (Figure R7). This supported our hypothesis that *LINC00565*-mediated changes in SMARCB1 binding lead to concordant alterations in gene expression, pointing out to a functional connection between the downregulated set and SMARCB1 impaired function.

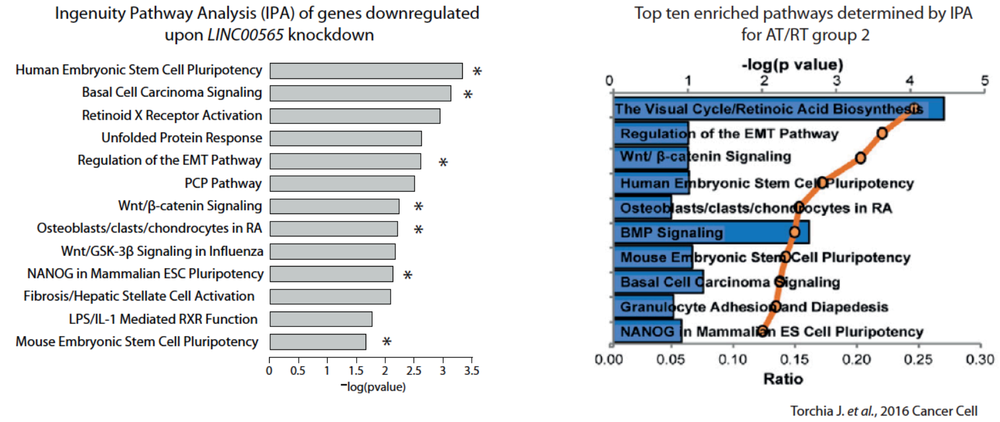

Figure R7. Functional terms enriched in genes downregulated after depletion of *LINC00565* (left) and gene signature characteristic of AT/RT epigenetic subgroup 2 (right), from which *PDFRB* was selected as therapeutic target [15]. The asterisks indicate the terms found in both datasets.

Following these premises, we found 85 genes with expression and SMARCB1 binding concordantly affected upon *LINC00565* knockdown. Out of these 85 genes, 83 (98%) were downregulated and had decreased SMARCB1 and H3K27Ac when *LINC00565* was depleted (Figure 5G and Supplementary Table 5). We believe that these include the primary targets of *LINC00565* and SMARCB1. Of note, 276 upregulated genes showed reduced SMARCB1 and H3K27ac when *LINC00565* was knocked down. Although difficult to interpret, we speculate that this is due to the accumulation of opposed transcriptional and posttranscriptional mechanisms that result in the increase of mRNA steady state levels that do not correlate with their change in epigenetic status.

10) The ChIRP studies in Fig. 5K are performed using lacZ as control. It is important to use a control which is expressed at a moderate to high level in the cells under study to determine the level of non-specific enrichment by the antisense pull down oligos. Similarly, for the Fig. 5L study, a locus that is expressed should be used to determine the level of enrichment of the captured chromatin loci.

Following the reviewer's suggestion, we repeated ChIRP assay pulling down in parallel to *LINC00565* an unrelated noncoding transcript, *CONCR*, a chromatin-enriched lncRNA with expression levels similar to *LINC00565* [16]. The pulldown of *CONCR* efficiently and specifically recovered *CONCR* RNA transcript (Figure R8A, added as Figure 5J). Nevertheless, *CONCR* did not result enriched at neither *GAS6* nor *PDGFRB* loci, while we detected its presence at its own TSS, confirming CONCR chromatin binding (Figure R8B, added as Figure 5K). These results confirmed *LINC00565* specific association to *GAS6* and *PDGFRB* genes, ruling out the possibility of false positives in our ChIRP experiment.

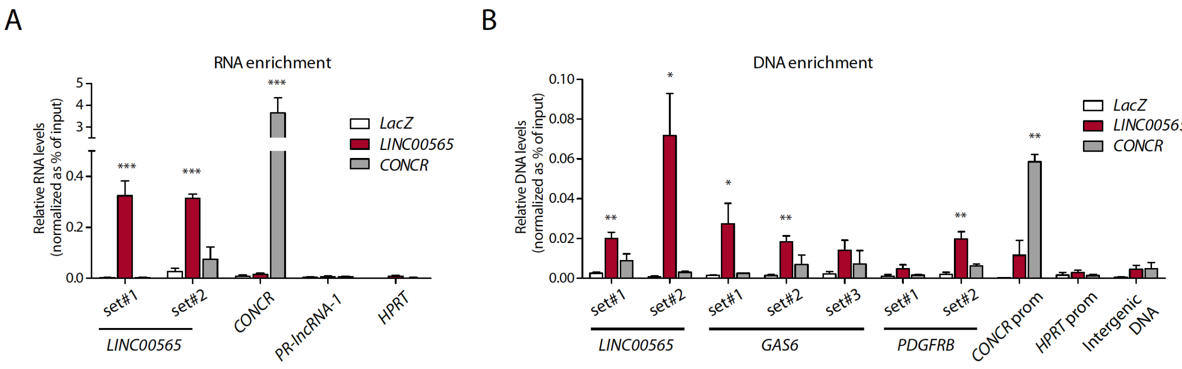

Figure R8. LINC00565 is localized at promoters of regulated genes. A) RNA enrichment in the ChIRP experiments performed with the indicated probes (LacZ, LINC00565 and CONCR) and quantified by RT-qPCR. **B)** Level of DNA enrichment in the ChIRP experiments performed with the indicated probes and quantified by qPCR.

11) Does knock down of PDGFRB at least partially recapitulate the impact of knock down of LINC00565? This is a rather simple study and should be performed, as the entire rationale for the studies detailed in the text after line 290 is the hypothesis that there must be other genes that mediate the impact of LINC00565 on proliferation. Even if knock down of PDGFRB regults in a small effect on proliferation, it will help validate the main point of this manuscript.

We appreciate the reviewer's suggestion that allowed us to validate the working model proposed in our study.

We depleted the expression of *PDGFRB* as well as *COL1A1*, another main *LINC00565* target, in lung cancer cells achieving good knockdown levels by using two different siRNAs for each gene (Figure R9, added as Supplementary Fig. 9B-D). We found that the independent downregulation of *PDGFRB* and *COL1A1* impaired cell growth, as observed by MTS proliferation assay (Figure R9C). This finding was in line with several studies reporting the roles for these two genes in different cancer types.

Please see this information expanded in the response to point 3 of Reviewer #3.

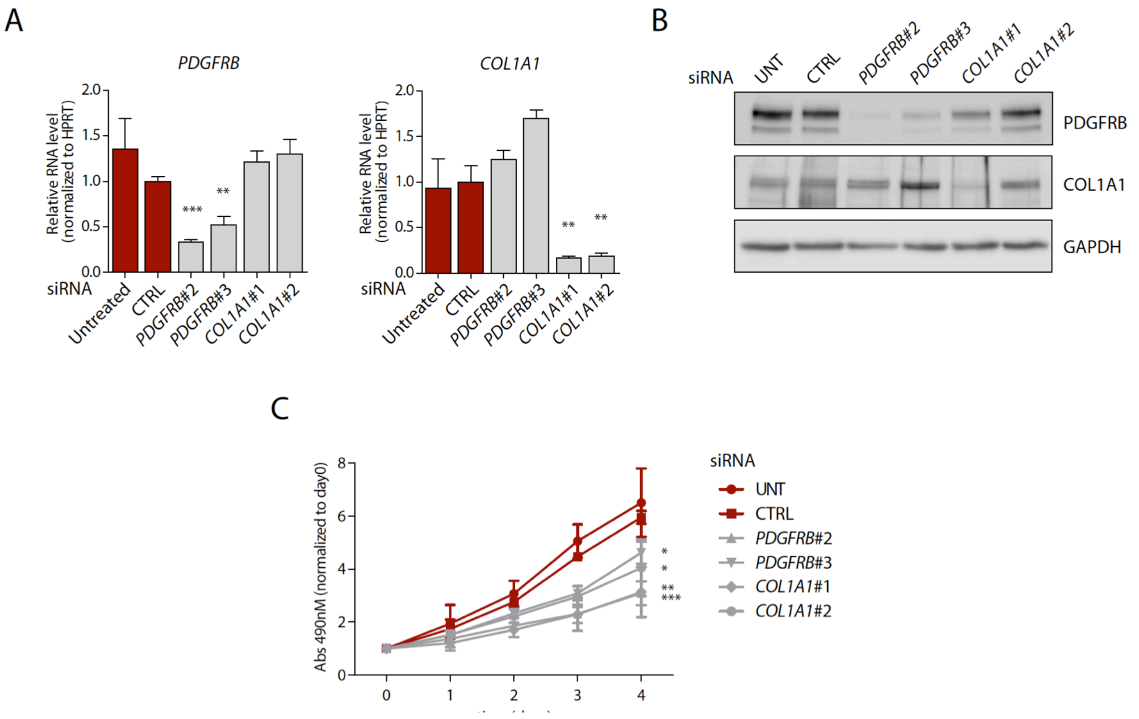

Figure R9. PDGFRB and COLA1 promote cell proliferation. Knockdown levels of PDGFB and COL1A1 achieved with two independent siRNAs in H226 cells determined by RT-qPCR **(A)**, and western blot **(B)**. **C.** Proliferation rate of H226 cells untransfected (UNT) or transfected with the indicted siRNAs.

Minor points:

1) Line 119: This sentence should be re-written, since in its current form it implies that the differential expression is playing the major role in defining the binding pattern.
The sentence has been modified

2) Line 175: the authors should comment on the annotations of this locus using a more recent version of GENCODE, e.g. V29 or 30 instead of V19. lncRNA annotations have undergone dramatic changes since V19.

We verified that the lncRNA annotation did not change in the most recent version of GENCODE (v31) compared to the one showed in our study. In both annotations, *LINC00565* corresponds to ENST00000562710.1 transcript with a length of 2331 bp. The only difference resides in the gene type annotation, as in GENCODE v19 *LINC00565* is defined as “lincRNA”, while in v31 it is reported as “lncRNA”, which is now adopted by GENCODE to unify several pre-existing subcategories of ncRNAs.
We have now updated the text accordingly.

3) Line 270: The data in Fig 4F and S5H show a reduced tumor growth rate, rather than reduced capacity of forming tumors. This should be corrected in the text.
This is corrected now.

4) Line 278: It should be clarified in this sentence that the observed effect in BJ cells is reduced proliferation.
This has been specified.

5) Are the GO terms listed in Fig. 4I positively or negatively enriched in response to the knock down of the lncRNA?

Figure 4I included both positively and negatively enriched GO terms.

6) Line 288: the authors are referring to Fig. 4G, not 5G.
This has been corrected.

7) Line 302: The signals were reduced, not lost. This should be corrected in the text.
Reviewer’s observation is correct. We modified the text accordingly.

Reviewer #3 (Remarks to the Author):

Long noncoding RNAs (lncRNAs) have traditionally been linked with gene silencing. In this manuscript, Grossi et al., explore a novel association between lncRNAs and SMARCB1- a core member of the multimeric SWI/SNF chromatin remodeling complex to regulate transcriptional activation. To explore this association, the authors employ a dynamic model of oncogene induced senescence in BJ fibroblasts, wherein the SWI/SNF complex is thought to be partly responsible for chromatin re-organization associated with the induction of senescence. The authors initially perform RNA immunoprecipitation with SMARCB1 and identify a previously uncharacterized enhancer lncRNA, LINC00565, as one of several that preferentially interact with the SWI/SNF complex in proliferating compared to senescent BJ fibroblasts. The authors confirm that SMARCB1 is mainly bound at active promoter and enhancer sites and demonstrate that SMARCB1 is bound to LINC00565. Grossi et al., show that SMARCB1 bound LINC00565 can functionally regulate the oncogene GAS6 within it’s co-located topologically associated domain (TAD), and further that this interaction is SMARCB1 dependent, at least in this cell context. Lastly, the authors demonstrate that LINC00565 promotes SMARCB1 occupancy at active proximal sites and can influence SMARCB1 regulated gene-expression programs.

Overall, the authors identify a novel enhancer lncRNA, LINC00565, and identify a functional link

with the SWI/SNF complex in regulating transcriptional activation at promoters. In general, the framing of the manuscript and data presentation is fairly clear. There are weaknesses in the manuscript, principally that it is unclear whether the data robustly support the conclusions and stated models.

We appreciate the reviewer's interest in our study.

Major points

• The chromatin complex that is being studied, SWI/SNF, is a very large complex and a concern is the potential for false positive findings of RNA interactions/function. This has previously been an issue as Prenser et al reported interaction of SMARCB1, a paper that was refuted earlier this year (both in Nature Genetics). Consequently, rigor and validation are critical concepts. The methods by which the interaction RNAs are indentified and statistically validated are rather brief and should be expanded to enhance confidence that the interactions are specific.

As the reviewer points out, SWI/SNF is a very large complex with variable conformations, thus the study of its interaction with RNA is not trivial. To be rigorous in the selection and validation of the lncRNA candidates after native SMARCB1 RIP, we have performed different analyses and controls. Reviewer #1 also had a related comment that we have addressed in detail. **Please see the response to point 5 of Reviewer #1, which we complement with additional data below.**

Per reviewer's request, we have expanded materials and methods section describing the pipeline used to select lncRNA candidates identified upon SMARCB1 RIP.

We have also included additional controls of specificity.
First, we wanted to exclude that the association between SMARCB1 and the selected lncRNAs may be due to unspecific interactions with chromatin proteins present at actively transcribed regions. For this, we performed native RIP of WDR5, a regulatory subunit of the MLL complex that facilitates H3K4me3 deposition and epigenetic activation, and which is known to bind lncRNAs that collaborate with its function [17-20]. We performed this experiment under the same conditions used for SMARCB1 RIP (Figure 1E). Despite the efficient pulldown of the known WDR5-interacting lncRNA *FOXD3-AS1* in proliferating cells [17], WDR5 RIP did not retrieve *LINC00565* or other lncRNA candidates isolated by SMARCB1 immunoprecipitation. (Figure R10, added as Figure 1E).

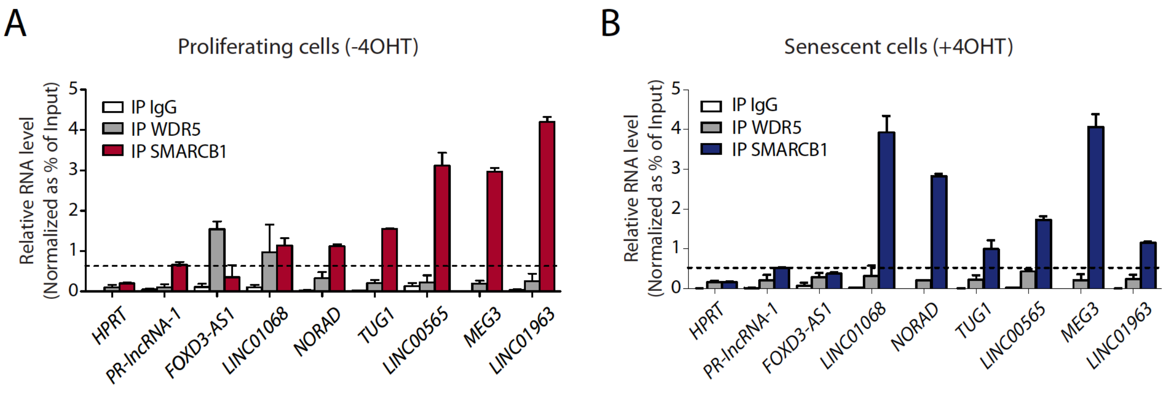

Figure R10. LINC00565 and other SMARCB1-interacting lncRNAs preferentially bind SMARCB1, but not WDR5. Native RIP experiment with the indicated antibodies in proliferating **(A)** or senescent BJ fibroblasts **(B).** *FOXD3-AS1* is a reported WDR5-interacting lncRNA, while *HPRT* and *PR-lncRNA-1* are shown as negative controls of both WDR5 and SMARCB1 RIP.

As further control we performed CLIP of the chromatin complex PRC2, known to be a prevalent binder of lncRNAs [21]. Also in this case, we were only able to detect enrichment of the positive controls but not most of the SMARCB1-interacting *LINC00565* or negative controls (Figure R3B, added to manuscript as Supplementary Fig. 1J).

These new data argue against the interaction between SWI/SNF and *LINC00565* being a false positive, which together with the body of work presented including (i) specific *in vitro* binding

between *LINC00565* and SMARCB1 (Figure R2), (ii) identification of a potential RNA-binding domain on SMARCB1 (Figure R1) and (iii) *LINC00565*-dependent effect on SMARCB1 binding to chromatin (Figure 5), support the role of the lncRNA in regulating SWI/SNF activity.

• With respect to this, the authors claim that the absence SMARCB1 is required for the interactions of LINC00565 and GAS6 and show that the interactions are absent in SMARCB1 deficient AT/RT cells. Subsequently, the authors show that the interactions between LINC00565 and GAS6 are context-dependent. Consequently, it is not the clear extent to which the model is accurate. To rigorously test the role of SMARCB1 in mediating these interactions, the authors could perform a rescue experiment to re-express SMARCB1 in AT/RT cells and test whether the LINC00565 and GAS6 interactions are restored.

We thank the reviewer for this suggestion that helps a more precise definition of our proposed model. As suggested, we have reintroduced SMARCB1 in CHLA-06 AT/RT cells using an inducible expression vector activated by doxycycline treatment (DOX) (Figure R11A). We then assessed the expression of SMARCB1-*LINC00565* target genes (*GAS6*, *PDGFRB* and *COL1A1*) in CTRL or *LINC00565* knockdown conditions (Figure R11B). We observed that the studied genes are induced when SMARCB1 is expressed in the cells, confirming that their expression depends on the protein (Figure R11B). However, this effect seems to be independent of *LINC00565.* This indicates that the re-introduction of SMARCB1 is not sufficient to establish SMARCB1-*LINC00565* regulation at these loci. We believe that an appropriate pre-existing chromatin conformation is required in order to favor the *LINC00565*-dependent gene regulatory mechanism. Similarly to HCT116, CHLA-06 cells do not have this favorable conformation, and since it is known that the reintroduction of SMARCB1 is not sufficient to reprogram the chromatin contacts[14], it is unable to bring co-regulated loci in physical proximity.

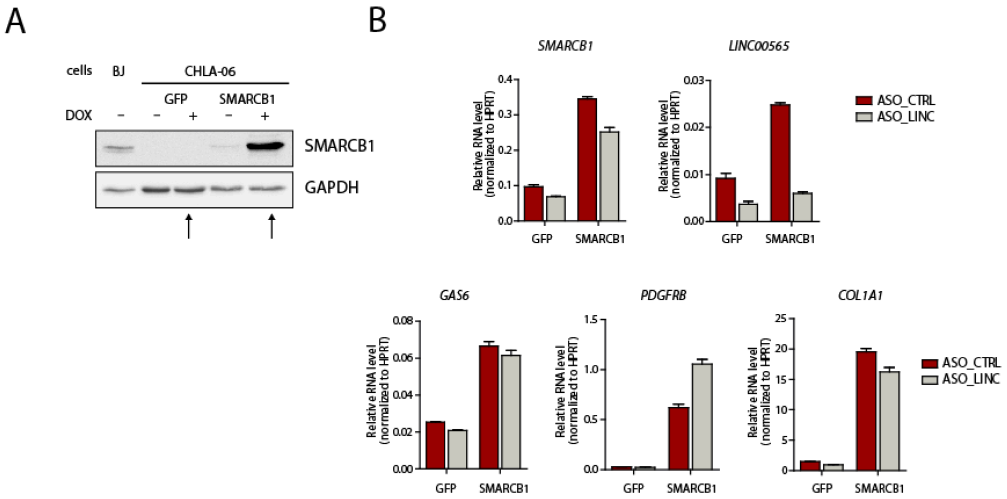

Figure R11. SMARCB1 expression in AT/RT cells is not sufficient to establish the regulation by SMARCB1-*LINC00565* of specific loci. A) SMARCB1 protein levels in BJ cells and CHL-06 cells transformed with control vector (GFP) or SMARCB1 vector +/- doxycycline treatment. The arrows indicate the experimental conditions used for the experiments shown in **(B). B)** Levels of the

.• In more detail, of the 1644 genes differentially regulated by LINC00565 knockdown, only 85 also showed LINC00565-dependent changes in SMARCB1 and chromatin status. Of those 85 genes, the researchers mention GAS6, PDGFRB, and COL1A1 as being part of "a LINC00565 and SWI/SNF-dependent oncogenic hub." (line 327). It is not clear how these three genes show that LINC00565 and SWI/SNF form an oncogenic hub. Showing data or discussing other work to support the importance of these genes +/- SMARCB1 and/or in other cited cancer contexts would have the potential to strengthen this conclusion.

The reviewer raises a pertinent question. With the expression "*LINC00565* and SWI/SNF-dependent oncogenic hub" we want to stress the concept that *LINC00565,* together with SMARCB1, are common upstream regulators of several genes that promote the highly proliferative phenotype of cancer cells, including *GAS6*, *PDGFRB* and *COL1A1*.
Several evidences support this idea:

(i) We show that the expression level of these genes is dependent of *LINC00565:* their mRNA levels are reduced when *LINC00565* is knocked down in cancer cells (Figure 5G), while they are increased upon upregulation of *LINC00565* by CRISPRa (Figure 6B). Moreover, we detect direct association of *LINC00565* to *GAS6* and *PDGFRB* regions by ChIRP (Figure R8 added as 5J-K).

(ii) These genes are regulated by SMARCB1, as shown by ChIP-seq, and this regulation is *LINC00565*-dependent, since *LINC00565* inhibition results in the decreased binding of SMARCB1 to their gene loci (Figures 5G-I).

(iii) The expression of *LINC00565* is positively correlated with the expression of several genes of the signature, including *GAS6*, *PDGFRB*, *PHLDB1*, *SPARC*, *COL4A1* and *COL1A1* in the TCGA lung squamous carcinoma (LUSC) cohort (Figures 4A, 6A and Supplementary Fig. 9A), which is consistent with their co-regulation by the lncRNA in patient samples.

We also made attempts to further address the functional connection between SMARCB1, *LINC00565,* and their co-regulated genes in the patients. For this, we tried to relate *SMARCB1* mutational status with the expression of this gene signature. Unfortunately, the percentage of tumors with *SMARCB1* reported mutations in TCGA datasets is very low (0.91% of LUSC, 0.25% of BRCA, 0.51% of LUAD, 0.36% of PRAD, 0.33% of KIRK or 2.34% of COAD), which impeded statistical analysis by comparing +/-SMARCB1 tumors within cohorts. Furthermore, correlating SMARCB1 mutational status to changes in gene expression is not trivial, as evident in AT/RTs where every mutational event drives distinct epigenetic patterns depending on the developmental stage in which they occur[15]. Nevertheless, we found that the expression of at least some of the *LINC00565* targets are highly dependent on SMARCB1 mutational status. In fact, both *PDGFRB* and *COL1A1* are reported to be misregulated in different rare cancer types directly driven by mutations in SWI/SNF components, such as atypical and malignant rhabdoid tumors (AT/RTs and MRTs, caused by SMARCB1 loss)[15,22] as well as small cell carcinoma of the ovary, hypercalcemic type (SCCOHT, due to concomitant SMARCA4 and SMARCA2 loss)[23].

(iv) We now show experimental data that corroborate the proposed model. We have performed depletion of *GAS6*, *PDGFRB* and *COL1A1* independently or in combination in lung squamous cancer cells and assessed their proliferation capacity. The proliferation of the cells is decreased when each of these genes is knocked down independently, while the simultaneous knockdown in combination with that of *GAS6,* results in an additive effect (Figure R12, added as Figure 6C-E). This observation is consistent with these genes being part of a network downstream of *LINC00565,* since the inhibition of *LINC00565* alone has a more pronounced effect that each of the three other genes individually (Figure 4D).

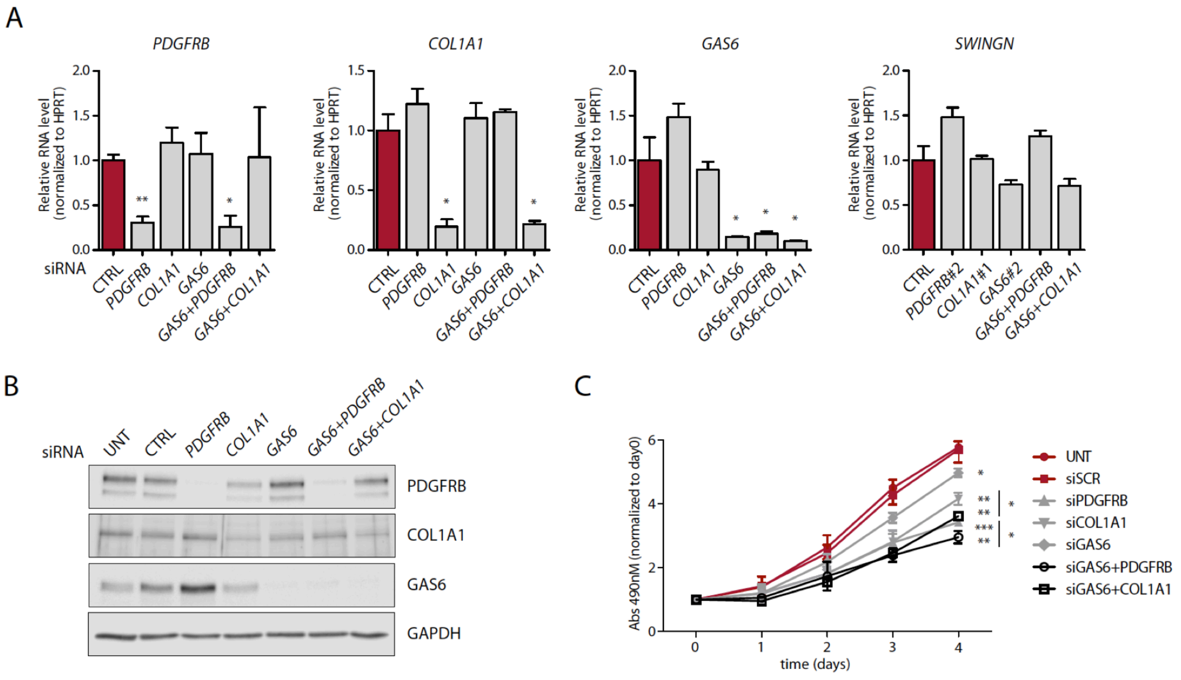

Figure R12. *GAS6, PDGFRB* and *COL1A1* contribute to the highly proliferative phenotype of H226 lung cancer cells. Knockdown levels of *PDGFB, COL1A1* and *GAS6* achieved in H226 cells determined by RT-qPCR **(A)** and western blot **(B). C.** Proliferation rate measured by MTS of H226 cells untransfected (UNT) or transfected with the indicated siRNAs.

(v) Abundant evidence already exists of the pro-oncogenic role of these genes. Besides the well-recognized oncogenic nature of *GAS6,* which we already discussed, several previously published studies indicate an oncogenic activity for *PDGFRB* and *COL1A1*:

Platelet derived growth factor receptor beta (*PDGFRB*) is a tyrosine kinase receptor activated upon PDGF binding. PDGFRB activation induces autophosphorylation and triggers intracellular signaling cascades that are crucial for evoking cellular responses such as migration and proliferation [24-26]. Due to its importance in tumor growth, several PDGF/PDGFR antagonists have been developed and few of them appeared to be successful in clinical trials, in particular for prostate cancer treatment [27].

Very recently, the effect of the PDGFRB inhibitor CP-673451 has been investigated, revealing that impairment of PDGFRB activity is sufficient to induce apoptosis in lung cancer cells and sensitizes them to cisplatin [28].

Collagen type I alpha 1 chain (*COL1A1*) encodes the major component of type I collagen. *COL1A1* overexpression has been functionally linked to breast, gastric, and colorectal cancer development, where it serves as oncoprotein by promoting metastasis [29-31]. In lung cancer, *COL1A1* levels have been recently found upregulated, though no functional analyses have been yet performed to assess its role in this tumor type. Our new data show for the first time to our knowledge, that *COL1A1* depletion affects cell proliferation of lung cancer cells.

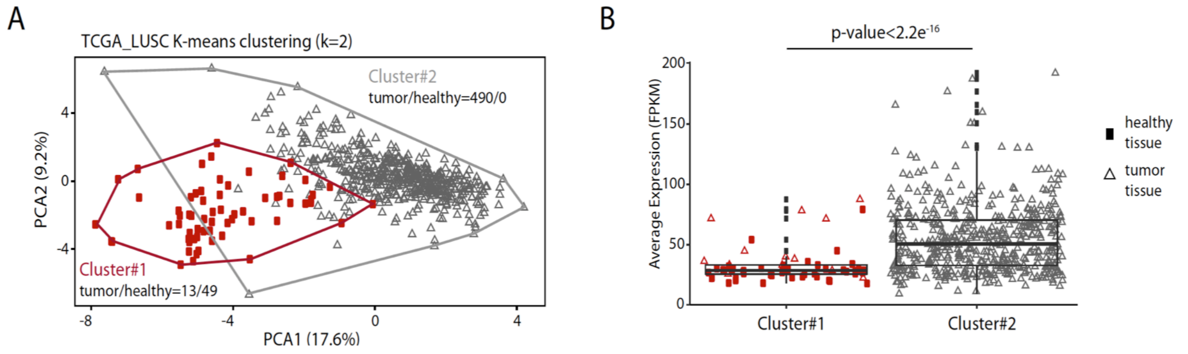

Figure R13. *LINC00565* oncogenic network predicts cancer status TCGA LUSC cohort. (A) K-means clustering of 552 samples according to the expression of the *LINC00565* gene signature **(B)** Average expression of the gene signature in each LUSC sample grouped according to cluster division (cluster#1 depicted in red; cluster#2 in grey). Different shapes reflect tumor vs healthy status. Statistics was calculated by Student's t-test

(vi) Finally, we decided to explore the potential clinical relevance of the SMARCB1 and *LINC00565*-activated gene network. To this aim, we selected the 27 top genes (i.e. the ones with the most affected expression after *LINC00565* KD) out of the 85 genes co-regulated by *LINC00565* and SMARCB1, and computed their expression across the LUSC cohort of TCGA, which includes 503 tumors and 49 samples of adjacent healthy tissue. Principal Component Analysis (PCA) and K-means clustering based on the expression of this signature separated the samples into two clusters (Figures R13A added as Figure 6F). Notably, cluster#1 contained all of the healthy tissues plus only 13 tumors, while cluster#2 included 490 tumors but not healthy tissue samples (Figure R13B, added as Figure 6F), suggesting a relationship between the transformed status and the expression of the SMARCB1-*LINC00565* signature. In agreement with *LINC00565* pro-proliferative role associated with gene activation, the expression of the signature was elevated in the tumor samples conforming cluster#2 as compared to cluster#1 samples, mostly composed of normal tissue samples (Figure R13B, added as Figure 6B).

• The work presented in Figure 2, the finding that SMARCB1 is present at both active enhancers and promoters is not novel. The cited papers show that SMARCB1 containing complexes BAF and PBAF have been shown to occupy active enhancers and promoters (Wang X et al., 2017, Nakyama et al., 2018).

We agree with the reviewer that the finding of SMARCB1 association to active promoters and enhancers had been already published in the two recent papers mentioned above, which came out during the progress of this project. However generating and validating quality ChIP-seq data of endogenous SMARCB1 in human fibroblasts is of relevance for the purpose of our study, since it is required in order to investigate consistent lncRNA- and SMARCB1-dependent gene regulatory effects in the same cell context.

Still, we believe that the ChIP-seq data generated might be useful to the scientific community for different reasons:

(i) The above-mentioned papers focused their findings related to SMARCB1-mediated activation of enhancers [14,32] and bivalent promoters [14] on rhabdoid tumor cells where the expression of SMARCB1 was restored by infection of lentiviral vectors encoding this SWI/SNF subunit. Although the authors demonstrated that the overexpressed SMARCB1 in principle behaves as the endogenous protein, it might present undetected differences. Moreover, although the authors corroborated some of their findings in other cell lines, the alteration of enhancer and promoter status was only reported using rhabdoid tumor cells, at least at the best of our knowledge. We believe that having these results confirmed with the endogenous protein in a non-tumoral cell line as human BJ fibroblasts might add value and consistency to those previous findings.

(ii) Beyond the finding of the presence of SMARCB1 at active chromatin regions, the SMARCB1 ChIP-seq data generated by us might be useful to other researchers.

In contrast to other SWI/SNF components, such as SMARCA4, very few SMARCB1 ChIP-seq datasets are available. SMARCB1 ChIP is a quite challenging experiment that requires optimization, which would benefit from the comparison to published data. Unfortunately, we found that the quality of ChIP-seq data for this particular SWI/SNF subunit was extremely low (see ChIP-seq data of [1]). Even the public ENCODE ChIP-seq data for SMARCB1 have been generated by using a santacruz antibody now discontinued (INI1 antibody sc-16189; ChIP-seq data generated by Snyder lab in the context of ENCODE consortium).
For these reasons, we believe that the results described in Figure 2 are an interesting resource that might contribute to prompt SWI/SNF research.

Minor points

• There are several grammatical/spelling issues
The text has been thoroughly revised

• The sentence that starts on line 136 is six lines long and quite difficult to understand.
We have edited this sentence for clarity.

• In Figure 2C, it would be helpful to see the SMARCB1 ChIP-Seq heatmap along-with H3K4me2, H3K4me3 and H3K27ac.
We have added SMARCB1 heatmap to the figure.

• The authors should cite Weissmiller et al., as they also have recently published SMARCB1 ChIP-Seq datasets.
In their Nature Comm manuscript, Weissmiller A.M. et al. [33] performed ChIP-seq analysis of MYC in rhabdoid tumor cells engineered to express inducible forms of EGFP, SMARCB1, or OmoMYC. They labeled MYC ChIP-seq tracks in SMARCB1-overexpressing conditions as “SNF5”, which might be misleading. However they didn’t publish SMARCB1 ChIP-seq, reason why we didn’t cite this study.

• The co-immunoprecipitation experiments performed in S1E are unconvincing and are of poor quality. They should either be removed from the figure panel or should be repeated for clearer results.

We agree that the quality of these blots is not optimal.
However we would like to note that the conditions used for these co-immunoprecipitations have been optimized for the detection of associated RNA. We are in fact able to obtain nicer co-immunoprecipitation blots changing the buffers, etc., but they wouldn’t correspond with the same experimental conditions used in the RIP experiments, which is the point that we want to stress with this figure. We apologize by the poor esthetics of the figure but it is an honest western blot that we believe illustrates the co-immunoprecipitation of the proteins shown.

• The differences seen in the tumor volumes in Figure 4F upon knockdown of LINC00565 in immunodeficient mice are at best modest and don’t add much to the manuscript.

We agree with the reviewer to consider the differences in mice tumor volumes modest. However, they represent a statistical significant effect, and the potential relevance should not be excluded. *LINC00565* depletion was achieved by transient transfection with a single ASO dose. It is reasonable to believe that a more persistent ASO treatment might result in a more pronounced decrease of tumor volume with a potential therapeutic effect.

BIBLIOGRAPHY

1 Prensner, J. R. *et al.* The long noncoding RNA SChLAP1 promotes aggressive prostate cancer and antagonizes the SWI/SNF complex. *Nat Genet* **45**, 1392-1398, doi:10.1038/ng.2771 (2013).

2 Raab, J. R. *et al.* SWI/SNF remains localized to chromatin in the presence of SCHLAP1. *Nat Genet* **51**, 26-29, doi:10.1038/s41588-018-0272-z (2019).

3 Jegu, T. *et al.* Xist RNA antagonizes the SWI/SNF chromatin remodeler BRG1 on the inactive X chromosome. *Nat Struct Mol Biol* **26**, 96-109, doi:10.1038/s41594-018-0176-8 (2019).

4 Flynn, R. A. *et al.* 7SK-BAF axis controls pervasive transcription at enhancers. *Nat Struct Mol Biol* **23**, 231-238, doi:10.1038/nsmb.3176 (2016).

5 Engreitz, J. M. *et al.* The Xist lncRNA exploits three-dimensional genome architecture to spread across the X chromosome. *Science* **341**, 1237973, doi:10.1126/science.1237973 (2013).

6 Schertzer, M. D. *et al.* lncRNA-Induced Spread of Polycomb Controlled by Genome Architecture, RNA Abundance, and CpG Island DNA. *Mol Cell*, doi:10.1016/j.molcel.2019.05.028 (2019).

7 Mumbach, M. R. *et al.* HiChIRP reveals RNA-associated chromosome conformation. *Nature methods* **16**, 489-492, doi:10.1038/s41592-019-0407-x (2019).

8 Atianand, M. K. *et al.* A Long Noncoding RNA lincRNA-EPS Acts as a Transcriptional Brake to Restrain Inflammation. *Cell* **165**, 1672-1685, doi:10.1016/j.cell.2016.05.075 (2016).

9 Lobley, A. E., Nugent, T., Orengo, C. A. & Jones, D. T. FFPred: an integrated feature-based function prediction server for vertebrate proteomes. *Nucleic Acids Res* **36**, W297-302, doi:10.1093/nar/gkn193 (2008).

10 Wang, Y. *et al.* The 3D Genome Browser: a web-based browser for visualizing 3D genome organization and long-range chromatin interactions. *Genome Biol* **19**, 151, doi:10.1186/s13059-018-1519-9 (2018).

11 Lieberman-Aiden, E. *et al.* Comprehensive mapping of long-range interactions reveals folding principles of the human genome. *Science* **326**, 289-293, doi:10.1126/science.1181369 (2009).

12 Li, J. *et al.* TANRIC: An Interactive Open Platform to Explore the Function of lncRNAs in Cancer. *Cancer Res* **75**, 3728-3737, doi:10.1158/0008-5472.CAN-15-0273 (2015).

13 Alver, B. H. *et al.* The SWI/SNF chromatin remodelling complex is required for maintenance of lineage specific enhancers. *Nat Commun* **8**, 14648, doi:10.1038/ncomms14648 (2017).

14 Nakayama, R. T. *et al.* SMARCB1 is required for widespread BAF complex-mediated activation of enhancers and bivalent promoters. *Nat Genet* **49**, 1613-1623, doi:10.1038/ng.3958 (2017).

15 Torchia, J. *et al.* Integrated (epi)-Genomic Analyses Identify Subgroup-Specific Therapeutic Targets in CNS Rhabdoid Tumors. *Cancer Cell* **30**, 891-908, doi:10.1016/j.ccell.2016.11.003 (2016).

16 Marchese , G. E., Marín-Béjar O, Bharti SK, Raimondi I, González J, Martínez-Herrera DJ, Athie A, Amadoz A, Brosh RM and Huarte M. A long noncoding RNA regulates sister chromatid cohesion. *Molecular cell* **In Press** (2016).

17 Subhash, S. *et al.* H3K4me2 and WDR5 enriched chromatin interacting long non-coding RNAs maintain transcriptionally competent chromatin at divergent transcriptional units. *Nucleic Acids Res* **46**, 9384-9400, doi:10.1093/nar/gky635 (2018).

18 Wang, K. C. *et al.* A long noncoding RNA maintains active chromatin to coordinate homeotic gene expression. *Nature* **472**, 120-124, doi:10.1038/nature09819 (2011).

19 Gomez, J. A. *et al.* The NeST long ncRNA controls microbial susceptibility and epigenetic activation of the interferon-gamma locus. *Cell* **152**, 743-754, doi:10.1016/j.cell.2013.01.015 (2013).

20 Yang, Y. W. *et al.* Essential role of lncRNA binding for WDR5 maintenance of active chromatin and embryonic stem cell pluripotency. *eLife* **3**, e02046, doi:10.7554/eLife.02046 (2014).

21 Marchese, F. P. & Huarte, M. Long non-coding RNAs and chromatin modifiers: their place in the epigenetic code. *Epigenetics : official journal of the DNA Methylation Society* **9**, 21-26, doi:10.4161/epi.27472 (2014).

22 Muscat, A. *et al.* Low-Dose Histone Deacetylase Inhibitor Treatment Leads to Tumor Growth Arrest and Multi-Lineage Differentiation of Malignant Rhabdoid Tumors. *Clin Cancer Res* **22**, 3560-3570, doi:10.1158/1078-0432.CCR-15-2260 (2016).
23 Lang, J. D. *et al.* Ponatinib Shows Potent Antitumor Activity in Small Cell Carcinoma of the Ovary Hypercalcemic Type (SCCOHT) through Multikinase Inhibition. *Clin Cancer Res* **24**, 1932-1943, doi:10.1158/1078-0432.CCR-17-1928 (2018).
24 Kazlauskas, A. Receptor tyrosine kinases and their targets. *Curr Opin Genet Dev* **4**, 5-14 (1994).
25 Heldin, C. H., Ostman, A. & Ronnstrand, L. Signal transduction via platelet-derived growth factor receptors. *Biochim Biophys Acta* **1378**, F79-113, doi:10.1016/s0304-419x(98)00015-8 (1998).
26 Kazlauskas, A. PDGFs and their receptors. *Gene* **614**, 1-7, doi:10.1016/j.gene.2017.03.003 (2017).
27 George, D. Targeting PDGF receptors in cancer--rationales and proof of concept clinical trials. *Adv Exp Med Biol* **532**, 141-151, doi:10.1007/978-1-4615-0081-0_12 (2003).
28 Yang, Y. *et al.* Inhibition of PDGFR by CP-673451 induces apoptosis and increases cisplatin cytotoxicity in NSCLC cells via inhibiting the Nrf2-mediated defense mechanism. *Toxicol Lett* **295**, 88-98, doi:10.1016/j.toxlet.2018.05.033 (2018).
29 Jansen, M. P. *et al.* Molecular classification of tamoxifen-resistant breast carcinomas by gene expression profiling. *J Clin Oncol* **23**, 732-740, doi:10.1200/JCO.2005.05.145 (2005).
30 Yasui, W. *et al.* Molecular-pathological prognostic factors of gastric cancer: a review. *Gastric Cancer* **8**, 86-94, doi:10.1007/s10120-005-0320-0 (2005).
31 Zhang, Z., Wang, Y., Zhang, J., Zhong, J. & Yang, R. COL1A1 promotes metastasis in colorectal cancer by regulating the WNT/PCP pathway. *Mol Med Rep* **17**, 5037-5042, doi:10.3892/mmr.2018.8533 (2018).
32 Wang, X. *et al.* SMARCB1-mediated SWI/SNF complex function is essential for enhancer regulation. *Nat Genet* **49**, 289-295, doi:10.1038/ng.3746 (2017).
33 Weissmiller, A. M. *et al.* Inhibition of MYC by the SMARCB1 tumor suppressor. *Nature communications* **10**, 2014, doi:10.1038/s41467-019-10022-5 (2019).

Reviewers' comments:

Reviewer #1 (Remarks to the Author):

Grossi et al. have a provided a timely and substantial update on their manuscript "A lncRNA-SWI/SNF complex crosstalk controls transcriptional activation at specific promoter regions". In the rebuttal letter they address the issues raised by the other reviewers and me. I.e., the authors now provide further biochemical and genomic evidence for a functional interaction of SMARCB1 and the newly coined SWINGN lincRNA. Restructuring of the manuscript and inclusion of the new data panels helped to improve it.
One area of concern remains in the conclusions for a direct "in trans activity" of SWINGN. The use of ASOs to suppress or CRISPRa to increase SWIGN levels, respectively, could be attributed to indirect effects. Knockdown of GAS6 seems to argue that at least for PDGFR and COLA1 the effect is not dependent on GAS6. The ChIRP-qPCR experiments in Fig. 5 seem to argue for a localization of SWINGN to GAS6 and PDGFR. ChIRP is known to be prone to technical artifacts. However, A ChIRP-Seq or a similar method could give an unbiased result for global binding of SWINGN. SWINGN seems to be low (<10) copy number RNA according to the authors own quantification. While it is beyond the scope of this paper to address this experimentally, it remains puzzling how a low copy RNA like SWINGN exerts specific effects at the selected SMARCB1 loci. Is there a spatiotemporal, nuclear structural component that involves long range interactions between interacting regions- can a modified in situ Hi-C be applied to address this concern? Is SWINGN one of several RNAs that could have a similar effect on the affected gene set by regulating SMARCB1, as the initial RIP experiments in Fig. 1 suggests? The authors should implement these few suggestions to reflect the complex body of the literature regarding these questions, as there is a danger of overstatement here..

Reviewer #2 (Remarks to the Author):

The revised version of the manuscript is strongly improved and many of the concerns and issues have been addressed. While there are still some problems remaining, the overall conclusion of the manuscript is now supported by the presented data.

Reviewer #3 (Remarks to the Author):

The authors have sought to address each of my concerns. While new data is useful in some places, one of the newly reported findings amplifies my original central concern about whether the data robustly support the conclusions and stated models.

My first major point was the possibility of non-specific interactions between a large chromatin remodeling complex and non-coding RNAs. The authors do a nice job with this by showing that neither WDR5 nor PRC2 chromatin proteins/complexes pull down LINC00565/SWINGN – only the SWI/SNF complex pulls it down. This new data provides enhanced support for the specificity of the interaction.

The challenge comes regarding my next comment:
With respect to this, the authors claim that the absence SMARCB1 is required for the interactions of LINC00565 and GAS6 and show that the interactions are absent in SMARCB1 deficient AT/RT cells. Subsequently, the authors show that the interactions between LINC00565 and GAS6 are context-dependent. Consequently, it is not the clear extent to which the model is accurate. To rigorously test the role of SMARCB1 in mediating these interactions, the authors could perform a rescue experiment to re-express SMARCB1 in AT/RT cells and test whether the LINC00565 and GAS6 interactions are restored.

The response from the authors is:
"We thank the reviewer for this suggestion that helps a more precise definition of our proposed model. As suggested, we have reintroduced SMARCB1 in CHLA-06 AT/RT cells using an inducible expression vector activated by doxycycline treatment (DOX) (Figure R11A). We then assessed the expression of SMARCB1-LINC00565 target genes (GAS6, PDGFRB and COL1A1) in CTRL or LINC00565 knockdown conditions (Figure R11B). We observed that the studied genes are induced when SMARCB1 is expressed in the cells, confirming that their expression depends on the protein (Figure R11B). However, this effect seems to be independent of LINC00565." (emphasis added). Indeed, the figure provided for reviewers, R11, shows this. The expression of SMARCB1 leads to the induction of GAS6, PDGFRB and COL1A1. Based upon the model advanced by the manuscript, this induction should be dependent upon LINC00565/SWINGN. But the data shows that knockdown of LINC00565/SWINGN has no effect. Not only is this a problem for the model but this new data is not presented and is indirectly explained away in the revised manuscript. The original manuscript cited the example of the CHLA-06 AT/RT cell line (SMARCB1-deficient) with a goal of expanding the original finding from a single cell context (BJ fibroblasts) to a cancer-relevant context. The original manuscript reported that depletion of LINC00565/SWINGN had no effect upon GAS6 in CHLA-06 cells and concluded that this demonstrated that SMARCB1 is required for this relationship. However, the authors have now directly tested this and find that SMARCB1 is capable of inducing these genes but that it does so in a LINC00565/SWINGN independent manner. The revised manuscript does not objectively reflect the new data. It now reads "Notably, GAS6 mRNA expression remained unchanged upon SWINGN downregulation, suggesting that SMARCB1 (and intact SWI/SNF) is required for SWINGN regulatory effect on GAS6 (Figure 3H), although a pre-existing appropriate chromatin conformation is also probably needed to establish the correct regulatory interactions. These data support the idea that SWINGN and SWI/SNF cooperate to regulate the expression of GAS6."

Thus, the same original conclusion is made, despite the fact that the new data shows it to be unsupported. The new data, which seems well done but produces a result inconsistent with the model, has not been included,and a non-specific statement of "appropriate chromatin conformation is also probably needed". This is not rigorous and is not an objective presentation of the data.

The evidence indicates that in BJ fibroblasts that both SMARCB1 and LINC00565/SWINGN are required for regulation of GAS6 but in a cell line cancer model derived from SMARCB1 inactivation that this relationship is not present. That relationship may be present in the H226 lung cell line, but is not present in HCT116 colon cell line. Later it is stated "We found 85 genes presenting concordantly altered expression, SMARCB1 and H3K27ac binding upon SWINGN knockdown (Figure 5G and Supplementary Table 5). Of note, 276 genes did not show a change in gene expression concordant with SMARCB1 altered binding when SWINGN was depleted. Although difficult to interpret, we speculate that this may be due to the accumulation of opposed transcriptional and posttranscriptional mechanisms that result in an increase of mRNA steady state levels that do not reflect their epigenetic status." Thus, data from 85 genes consistent with the model is focused upon but data from 276 genes are inconsistent. In the discussion, the authors state "The presence of SMARCB1, ensuring the formation of intact SWI/SNF complexes, as well as an appropriate chromatin conformation allowing the physical proximity between SWINGN and the regulated loci, are necessary for this regulatory mechanism." But the data provided to reviewers demonstrates that SMARCB1 can regulate the target loci independent of LINC00565/SWINGN.

The responses to the minor points are all fine.

In the end, the authors have some seemingly real associations between a chromatin complex and an lncRNA, but these associations are quite context dependent and thus the broad conclusions seem overstated, including the omission of key contradictory data and objective discussion. It is thus difficult to have confidence that the data robustly supports the conclusions and stated models.

Reviewers' comments:

Reviewer #1 (Remarks to the Author):

Grossi et al. have a provided a timely and substantial update on their manuscript “A lncRNA-SWI/SNF complex crosstalk controls transcriptional activation at specific promoter regions”. In the rebuttal letter they address the issues raised by the other reviewers and me. I.e., the authors now provide further biochemical and genomic evidence for a functional interaction of SMARCB1 and the newly coined SWINGN lincRNA. Restructuring of the manuscript and inclusion of the new data panels helped to improve it.

One area of concern remains in the conclusions for a direct “in trans activity” of SWINGN. The use of ASOs to suppress or CRISPRa to increase SWIGN levels, respectively, could be attributed to indirect effects. Knockdown of GAS6 seems to argue that at least for PDGFR and COLA1 the effect is not dependent on GAS6. The ChIRP-qPCR experiments in Fig. 5 seem to argue for a localization of SWINGN to GAS6 and PDGFR. ChIRP is known to be prone to technical artifacts. However, A ChIRP-Seq or a similar method could give an unbiased result for global binding of SWINGN. SWINGN seems to be low (<10) copy number RNA according to the authors own quantification. While it is beyond the scope of this paper to address this experimentally, it remains puzzling how a low copy RNA like SWINGN exerts specific effects at the selected SMARCB1 loci. Is there a spatiotemporal, nuclear structural component that involves long range interactions between interacting regions- can a modified in situ Hi-C be
applied to address this concern? Is SWINGN one of several RNAs that could have a similar effect on the affected gene set by regulating SMARCB1, as the initial RIP experiments in Fig. 1 suggests? The authors should implement these few suggestions to reflect the complex body of the literature regarding these questions, as there is a danger of overstatement here.

We thank the reviewer for considering the revised version of our manuscript substantially improved.

We agree with him/her that the exact mechanism by which *SWINGN* regulates different distant genes is not fully elucidated.
However, we would like to point out that we are reluctant to apply a classical definition of “in trans activity” to this particular lncRNA. In fact, we believe that *SWINGN*-mediated transcriptional control of distant DNA loci is allowed by a precise spatial chromatin organization around *SWINGN* enhancer region that brings together multiple distant genomic regions.
This “*cis-trans*” model is well supported by a number of papers that recently developed different ligation-free methods to capture long-range as well as multiple/concomitant DNA interactions in the nucleus [1-3].
For instance, Beagrie et al. coupled Genome Architecture Mapping (GAM) and Cryo-FISH to investigate multi-enhancer contacts and found that transcribed enhancer regions are more prone to engage multiple interactions (“triplets”) with distant genomic regions. This finding has been expanded by Quinodoz et al., thanks to the development of SPRITE method, which highlights how transcribed genomic regions are able to form multiple interactions (3-14 concomitant interactions) not only with distant loci (>100Mb) on the same chromosome but also inter-chromosomal contacts. Even though these novel techniques would allow a more precise mapping of *SWINGN* locus 3D interactions compared to ligation-based 3C/HiC methods, we believe that applying them would be beyond the scope of our paper for their high cost in terms of time and resources (SPRITE technique requires ~1.5 billion sequencing reads from each sample for optimal coverage).

In this context of spatial proximity where stochastic alternative contacts can be established with multiple loci, even a few molecules of lncRNA are reasonably sufficient to drive transcriptional activation mediated by the presence of SMARCB1.
This hypothesis is supported by a number of publications reporting that low-abundant lncRNAs are able to contact multiple loci, such as *lincRNA-EPS* (~10 copies/cell) and *Airn* (~8 copies/cell), as we briefly described in our previous rebuttal letter [4,5]. These different studies pointed out that additional features besides RNA abundance contribute to lncRNA-mediated function, as the presence of convenient chromatin architecture and their association to RNA-binding proteins (HNRNP-L and PRCs, respectively).
Taking into account this generally accepted model, we confidently list *SWINGN* in this group of lncRNAs with a mechanism of function in between the classical "*cis-trans*" division. We now expand the manuscript discussion to discuss this topic.

Even though we can only speculate that other SMARCB1-interacting lncRNAs might function similarly, we provide robust data demonstrating a direct effect of *SWINGN* on at least some of its targets. In fact, although we are aware of possible technical artifacts in ChIRP-qPCR method, we introduced multiple controls to ensure the specificity of *SWINGN* binding to particular DNA regions, as correctly suggested by two the referees.

Reviewer #2 (Remarks to the Author):

The revised version of the manuscript is strongly improved and many of the concerns and issues have been addressed. While there are still some problems remaining, the overall conclusion of the manuscript is now supported by the presented data.

We thank the reviewer for this positive assessment. We greatly appreciated his/her insightful comments and suggestions that remarkably improved our manuscript.

Reviewer #3 (Remarks to the Author):

The authors have sought to address each of my concerns. While new data is useful in some places, one of the newly reported findings amplifies my original central concern about whether the data robustly support the conclusions and stated models.

We thank the reviewer for appreciating the extensive work carried out in order to address the concerns of the three reviewers. However, although we acknowledge that we didn't discuss some of the new results sufficiently, we respectfully disagree that the new data presented do not support the proposed model. Please see our reasoning below.

My first major point was the possibility of non-specific interactions between a large chromatin remodeling complex and non-coding RNAs. The authors do a nice job with this by showing that neither WDR5 nor PRC2 chromatin proteins/complexes pull down LINC00565/SWINGN – only the SWI/SNF complex pulls it down. This new data provides enhanced support for the specificity of the interaction.

The challenge comes regarding my next comment:
With respect to this, the authors claim that the absence SMARCB1 is required for the interactions of LINC00565 and GAS6 and show that the interactions are absent in SMARCB1 deficient AT/RT cells. Subsequently, the authors show that the interactions between LINC00565 and GAS6 are context-dependent. Consequently, it is not the clear extent to which the model is accurate. To

rigorously test the role of SMARCB1 in mediating these interactions, the authors could perform a rescue experiment to re-express SMARCB1 in AT/RT cells and test whether the LINC00565 and GAS6 interactions are restored.

The response from the authors is:
"We thank the reviewer for this suggestion that helps a more precise definition of our proposed model. As suggested, we have reintroduced SMARCB1 in CHLA-06 AT/RT cells using an inducible expression vector activated by doxycycline treatment (DOX) (Figure R11A). We then assessed the expression of SMARCB1-LINC00565 target genes (GAS6, PDGFRB and COL1A1) in CTRL or LINC00565 knockdown conditions (Figure R11B). We observed that the studied genes are induced when SMARCB1 is expressed in the cells, confirming that their expression depends on the protein (Figure R11B). However, this effect seems to be independent of LINC00565." (emphasis added). Indeed, the figure provided for reviewers, R11, shows this. The expression of SMARCB1 leads to the induction of GAS6, PDGFRB and COL1A1. Based upon the model advanced by the manuscript, this induction should be dependent upon LINC00565/SWINGN. But the data shows that knockdown of LINC00565/SWINGN has no effect. Not only is this a problem for the model but this new data is not presented and is indirectly explained away in the revised manuscript. The original manuscript cited the example of the CHLA-06 AT/RT cell line (SMARCB1-deficient) with a goal of expanding the original finding from a single cell context (BJ fibroblasts) to a cancer-relevant context. The original manuscript reported that depletion of LINC00565/SWINGN had no effect upon GAS6 in CHLA-06 cells and concluded that this demonstrated that SMARCB1 is required for this relationship. However, the authors have now directly tested this and find that SMARCB1 is capable of inducing these genes but that it does so in a LINC00565/SWINGN independent manner. The revised manuscript does not objectively reflect the new data. It now reads "Notably, GAS6 mRNA expression remained unchanged upon SWINGN downregulation, suggesting that SMARCB1 (and intact SWI/SNF) is required for SWINGN regulatory effect on GAS6 (Figure 3H), although a pre-existing appropriate chromatin conformation is also probably needed to establish the correct regulatory interactions. These data support the idea that SWINGN and SWI/SNF cooperate to regulate the expression of GAS6."

Thus, the same original conclusion is made, despite the fact that the new data shows it to be unsupported. The new data, which seems well done but produces a result inconsistent with the model, has not been included,and a non-specific statement of "appropriate chromatin conformation is also probably needed". This is not rigorous and is not an objective presentation of the data.

We understand the reviewer's concern and apologize for not fully integrating the new results into the manuscript. We have tried to solve it, and believe that our model takes into consideration all the available data:

We show that in non-tumor cells (BJ foreskin and IMR90 fetal lung fibroblasts) as well as in cancer cell lines (H226 lung squamous and A498 clear renal carcinoma cells [see first rebuttal letter, R6]) the regulation of *GAS6* and other target genes is dependent on *SWINGN*-SMARCB1 axis. Multiple experimental evidences prove that not only the lncRNA, but also SMARCB1 (and other subunits of SWI/SNF complex) are necessary for the full activation of the target genes (see Figures 3E-F, 4B-C, 4G-H, 6B, Supplementary Figures 4G, 5B and 6E). This is consistent with the role proposed for *SWINGN*-SMARCB1, which enhances transcriptional activation of specific gene loci.

In addition, we did a great effort in order to investigate this regulatory mechanism in multiple cell models, not only including the aforementioned cell lines, but also colorectal and AT/RT cancer

cells, where we found that SWINGN-SMARCB1 dependency is absent. In HCT116 colorectal cells *SWINGN* levels are lower than in other cell lines, leading us to the initial hypothesis that when *SWINGN* is lowly expressed, the topological connection between the two chromatin regions cannot be formed and the regulatory hub is disrupted. Although this hypothesis is in agreement with public ChIA-PET data, showing lack of interactions between the enhancer and *GAS6* loci in this cell line (ENCODE data – GSM970210), it cannot be concluded which one is cause or consequence (the high expression of *SWINGN-GAS6* or the physical contact of the loci). Nonetheless, these data raised the possibility that *SWINGN* and SMARCB1 expression are not sufficient for *SWINGN*-SMARCB1 regulatory crosstalk to occur unless the proper chromatin conformation is present.

To determine that, we used AT/RT cell line, testing the requirement of SMARCB1 in a natural genetic model. Now, thanks to the reviewer's suggestion, we have additional information to better understand the role of *SWINGN*. The enforced expression of SMARCB1 in AT/RT cell line indeed results in an activation of target genes, however this activation cannot be modulated by *SWINGN.* We interpret that this experimental setting provides high amounts of SMARCB1, so SWI/SNF can bind and induce the expression of permissive genes. However, the lack of the proper chromatin topology impedes the fine regulation by *SWINGN*. This idea is fully consistent with the work by Nakayama *et al.*, which shows that SMARCB1 overexpression in AT/RT cells is not sufficient to globally reprogram their chromatin conformation, as shown by Hi-C experiments[6].

In other words, we don't claim that the expression of *SWINGN* and SMARCB1 per se are sufficient to drive transcriptional activation, which perhaps wasn't clearly stated in our manuscript. We now have included these new data and are more careful with the wording of our proposed model.

The evidence indicates that in BJ fibroblasts that both SMARCB1 and LINC00565/SWINGN are required for regulation of GAS6 but in a cell line cancer model derived from SMARCB1 inactivation that this relationship is not present. That relationship may be present in the H226 lung cell line, but is not present in HCT116 colon cell line. Later it is stated "We found 85 genes presenting concordantly altered expression, SMARCB1 and H3K27ac binding upon SWINGN knockdown (Figure 5G and Supplementary Table 5). Of note, 276 genes did not show a change in gene expression concordant with SMARCB1 altered binding when SWINGN was depleted. Although difficult to interpret, we speculate that this may be due to the accumulation of opposed transcriptional and posttranscriptional mechanisms that result in an increase of mRNA steady state levels that do not reflect their epigenetic status." Thus, data from 85 genes consistent with the model is focused upon but data from 276 genes are inconsistent.

As pointed out by the reviewer and stated in our manuscript, we focused on the study of the 85 genes with consistent chromatin and gene expression changes. When selecting these genes, we were agnostic of the direction of these changes, which resulted to be overwhelmingly in the same direction as *GAS6* for the three variables analyzed; i.e. the knockdown of *SWINGN* results in the decrease of SMARCB1 and H3K27Ac occupancy as well as decreased expression. Unfortunately we could not draw any conclusions on the remaining genes. Although their chromatin is affected mainly in the same manner (the knockdown of *SWINGN* causes a decrease of SMARCB1 and H3K27Ac occupancy), their steady-state mRNA levels are increased. Since the literature does not support transcriptional activation linked to a decrease of promoter H3K27Ac mark, we speculate that the mRNA steady-state levels are further regulated post-transcriptionally.
Nevertheless, our conclusions are limited to the loci that we were able to verify experimentally, including *GAS6* and several others, which are part of the pro-oncogenic gene signature in lung squamous cancer.

In the discussion, the authors state "The presence of SMARCB1, ensuring the formation of intact SWI/SNF complexes, as well as an appropriate chromatin conformation allowing the physical proximity between SWINGN and the regulated loci, are necessary for this regulatory mechanism." But the data provided to reviewers demonstrates that SMARCB1 can regulate the target loci independent of LINC00565/SWINGN.

We apologize for not using the language with sufficient precision. As explained above, the enforced expression of SMARCB1 in AT/RT cells can lead to the activation of genes, however it is probably not sufficient to re-establish the enhancer-promoter chromatin contacts necessary for the regulation by *SWINGN*-SMARCB1 axis.

The responses to the minor points are all fine.

In the end, the authors have some seemingly real associations between a chromatin complex and an lncRNA, but these associations are quite context dependent and thus the broad conclusions seem overstated, including the omission of key contradictory data and objective discussion. It is thus difficult to have confidence that the data robustly supports the conclusions and stated models.

We have now toned down some of our conclusions and modified the discussion to reflect more accurately the data provided in our study. We hope the reviewer appreciates that although limited to *SWINGN,* our observations could be of relevance to other gene loci where interactions between SMARCB1 and *cis*-produced lncRNAs take place.

1 Beagrie, R. A. *et al.* Complex multi-enhancer contacts captured by genome architecture mapping. *Nature* **543**, 519-524, doi:10.1038/nature21411 (2017).

2 Quinodoz, S. A. *et al.* Higher-Order Inter-chromosomal Hubs Shape 3D Genome Organization in the Nucleus. *Cell* **174**, 744-757 e724, doi:10.1016/j.cell.2018.05.024 (2018).

3 Marti-Renom, M. A. *et al.* Challenges and guidelines toward 4D nucleome data and model standards. *Nat Genet* **50**, 1352-1358, doi:10.1038/s41588-018-0236-3 (2018).

4 Atianand, M. K. *et al.* A Long Noncoding RNA lincRNA-EPS Acts as a Transcriptional Brake to Restrain Inflammation. *Cell* **165**, 1672-1685, doi:10.1016/j.cell.2016.05.075 (2016).

5 Schertzer, M. D. *et al.* lncRNA-Induced Spread of Polycomb Controlled by Genome Architecture, RNA Abundance, and CpG Island DNA. *Mol Cell* **75**, 523-537 e510, doi:10.1016/j.molcel.2019.05.028 (2019).

6 Nakayama, R. T. *et al.* SMARCB1 is required for widespread BAF complex-mediated activation of enhancers and bivalent promoters. *Nat Genet* **49**, 1613-1623, doi:10.1038/ng.3958 (2017).

REVIEWERS' COMMENTS:

Reviewer #1 (Remarks to the Author):

The authors have softened their conclusions regarding some of the more problematic experiments (e.g. ChIRP-qPCR) and added some more language responding to our points of concern how a low expressed lncRNA can act in a "cis/trans" capacity (suggesting it acts in cis but the loci come together locally, no data) They cite literature for other lncRNAs doing that (not convincing) and cite that SPRITE-Seq and other methods that can suggest such interactions, they provide no new data for their specific lncRNA.
While the authors do not provide new experimental data to support their hypothesis that "SWINGN-mediated transcriptional control of distant DNA loci is achieved by a precise spatial chromatin organization around SWINGN enhancer region", they have changed the language of the manuscript to discuss this points of concern and cite relevant literature. The hypothesis is neither proven nor disproven at this point in my opinion, but as the authors do present novel and interesting findings about SMARCB1, SWINGN and its pro-ocogenic network, this is a solid manuscript that falls short of exciting new news but is sufficiently incremental to warrant accepting the manuscript.

Reviewer #3 (Remarks to the Author):

While there continue to be complexity in understanding this genetic regulatory mechanism, the revised conclusions are in general now supported by the data and importantly provide a more balanced evaluation of the data and conclusions. I have no further critiques.
